# Soil-MobiNet: A Convolutional Neural Network Model Base Soil Classification to Determine Soil Morphology and Its Geospatial Location

**DOI:** 10.3390/s23156709

**Published:** 2023-07-27

**Authors:** Emmanuel Kwabena Gyasi, Swarnalatha Purushotham

**Affiliations:** School of Computer Science and Engineering, VIT University, Vellore 632014, India; emmanuel.kwabena@vit.ac.in

**Keywords:** Munsell color chart, precision agriculture, urvara and usara, smartphone, sensors, soil morphology, depthwise pointwise convolution, VITSoil dataset, artificial intelligence, geospatial location

## Abstract

Scholars have classified soil to understand its complex and diverse characteristics. The current trend of precision agricultural technology demands a change in conventional soil identification methods. For example, soil color observed using Munsell color charts is subjective and lacks consistency among observers. Soil classification is essential for soil management and sustainable land utilization, thereby facilitating communication between different groups, such as farmers and pedologists. Misclassified soil can mislead processes; for example, it can hinder fertilizer delivery, affecting crop yield. On the other hand, deep learning approaches have facilitated computer vision technology, where machine-learning algorithms trained for image recognition, comparison, and pattern identification can classify soil better than or equal to human eyes. Moreover, the learning algorithm can contrast the current observation with previously examined data. In this regard, this study implements a convolutional neural network (CNN) model called Soil-MobiNet to classify soils. The Soil-MobiNet model implements the same pointwise and depthwise convolutions of the MobileNet, except the model uses the weight of the pointwise and depthwise separable convolutions plus an additional three dense layers for feature extraction. The model classified the Vellore Institute of Technology Soil (VITSoil) dataset, which is made up of 4864 soil images belonging to nine categories. The VITSoil dataset samples for Soil-MobiNet classification were collected over the Indian states and it is made up of nine major Indian soil types prepared by experts in soil science. With a training and validation accuracy of 98.47% and an average testing accuracy of 93%, Soil-MobiNet showed outstanding performance in categorizing the VITSoil dataset. In particular, the proposed Soil-MobiNet model can be used for real-time soil classification on mobile phones since the proposed system is small and portable.

## 1. Introduction

The field of study known as “soil science” focuses particularly on the traits and characteristics of different types of soils. Due to its overlap with several disciplines, including agronomy, geology, and biology, it is considered an interdisciplinary study. To comprehend the physical, chemical, and biotic elements that contribute to soil function and how they impact the environment, soil science has advanced over time. The contributions that soil science research makes to many facets of our existence demonstrate how important this field is. With the advances in modern technologies, scholars have used information technologies to obtain, process, and analyze multisource data with a high spatiotemporal resolution for decision making and operations in crop production management [1]. Precision agricultural technology is the current trend demanding the development of improved soil identification methods [2]. In particular, scholars have attempted to classify soil to understand its complex and diverse characteristics. 

The accurate classification and understanding of soil morphology and its geospatial location are crucial for various fields, including agriculture, land management, urban planning, and environmental monitoring. Traditionally, soil classification has relied on manual techniques and field surveys conducted by experts, which can be time consuming, labor intensive, and subject to human error. However, recent advancements in technology, particularly in the field of machine learning, offer promising solutions to automate and streamline this process. In recent years, convolutional neural networks (CNNs) have demonstrated remarkable performance in various image recognition tasks, such as object detection, facial recognition, and medical imaging. CNNs are well-suited for analyzing complex spatial patterns in data, making them a suitable choice for soil classification based on soil morphology. Moreover, the widespread adoption of smartphones with powerful computational capabilities provides an opportunity to leverage CNN models for on-the-go soil classification. 

This paper presents “Soil-MobiNet”, a novel convolutional neural network model designed for soil classification to determine soil morphology and its geospatial location. The objective of Soil-MobiNet is to enable accurate and real-time classification of soil types using smartphone devices, thus empowering farmers, researchers, and land managers with a portable and accessible tool for soil analysis. The proposed Soil-MobiNet model leverages the rich imaging capabilities of smartphones, including high-resolution cameras, location data, and computational power, to capture soil images and analyze them using advanced deep learning techniques. By integrating soil morphology analysis with geospatial information, Soil-MobiNet aims to provide a comprehensive understanding of soil characteristics and their spatial distribution, which can facilitate informed decision making in various domains. The key contributions of this research can be summarized as follows:

- Development of a convolutional neural network model specifically tailored for soil classification, considering the unique characteristics and complexities of soil morphology analysis.
- Integration of geospatial information with soil classification, enabling the determination of the precise location of different soil types and their spatial distribution.
- Optimization of the Soil-MobiNet model to ensure real-time inference on resource-constrained smartphone devices, without compromising classification accuracy.
- Validation of the Soil-MobiNet model through extensive experiments and comparative analysis with existing soil classification methods, demonstrating its effectiveness and practicality for on-the-go soil analysis.

The remainder of this paper is organized as follows: In Section 2, we provide an overview of related work in the field of soil classification and explore the existing techniques and challenges. Section 3 presents the methodology behind Soil-MobiNet, detailing the model architecture, dataset and data preprocessing used, and training process. Experimental results and analysis are discussed in Section 4. The implementation of the model on a smartphone is in Section 5. Finally, Section 6 concludes the paper, highlighting the contributions, limitations, and potential future research directions of Soil-MobiNet.

By harnessing the power of convolutional neural networks and the ubiquity of smartphones, Soil-MobiNet opens new possibilities for efficient, accurate, and accessible soil classification. It holds the potential to revolutionize soil analysis practices, enabling stakeholders to make informed decisions about soil management, crop selection, land use planning, and environmental preservation, all while leveraging the convenience and portability of smartphones.

## 2. Related Works

Various relief features, climatic realms, landforms, and vegetation have contributed to the development of numerous soil types, particularly in India. In ancient times, around the 16th century AD, Indian soils were classified primarily into two categories: “Urvara” and “Usara”, implying fertile and sterile, respectively [3]. Over time, the soil has been classified based on its characteristic features such as moisture content, texture, color, and slope of the land. In particular, the soil has been identified based on texture as sandy, clayey, silty, and loam. Moreover, the soil has been identified in terms of colors such as red, yellow, and black. Currently, the Indian Council of Agricultural Research (ICAR) classifies Indian soil based on its character and nature, following the United States Department of Agriculture (USDA) soil taxonomy [4].

Based on composition, genesis, color, and location, the ICAR classified Indian soils as (i) alluvial soils, (ii) arid soils, (iii) black soils, (iv) forest soils, (v) laterite soils, (vi) peaty soils, (vii) red and yellow soils, and (viii) saline soils. These soils exhibit unique characteristics and historical antecedents. For instance, Khadar and Bhangar are two distinct types of alluvial soil found in the upper and middle Ganga plains in India. In particular, Khadar is annually deposited by floods and is rich in fine silt, whereas Bhangar is deposited far from the floodplain, representing a system of older alluvium. Khadar and Bhangar comprise calcareous concentrations (Kankars), primarily clayey and loamy, in the Brahmaputra Valley and the lower and middle Ganga plains.

Soil classification is essential for soil management and sustainable land utilization [5], which can help communication between different groups such as farmers and pedologists. However, misclassified soil can mislead processes; for instance, it can hinder fertilizer delivery, affecting crop yield. Several researchers have investigated ways to identify soil types and estimate their properties, as the human eye can be unreliable for color determination [6].

The *Munsell Soil Color Book* comprises color charts that evaluate soil types in a particular place. This book is used in the field to conduct soil color evaluations. The soil classification system developed around the Munsell color system is the conventional method for assigning soil types [7]. However, this method has shown accuracy problems when identifying the color of soil specimens using Munsell charts [8,9]. These problems are related to the three main factors affecting the psychophysical characteristics of color: illumination conditions, sample characteristics, and the observer’s sensitivities, knowledge, experience, and color vision. Therefore, the soil color observed using Munsell color charts (MCC) is subjective and lacks consistency among observers.

Baumann et al. [10] outlined the strong relationship between soil color and other essential soil properties and characteristics, such as soil organic matter content, mineral composition, land suitability, soil fertility, soil drainage class, and soil moisture. According to Thompson et al. [11] and Pendleton and Nickerson [7], the conventional method determines the soil color by comparing it with MCCs. MCCs allow users to identify soil colors varying from red to blue. These charts also help identify the humus and iron content in the soil [12]. MCCs define soil color based on three-color dimensions: hue, value, and chroma, which indicate the dominant wavelength, lightness, and saturation, respectively.

Nevertheless, the primary limitations of using MCCs include (a) environmental conditions (e.g., lighting conditions and moisture content [13]) and (b) user sensitivity (e.g., subjectivity and color blindness). Ibanez-Asensio et al. [14] considered only visible-wavelength light to estimate soil characteristics; the proposed method was effective in some ways. Visible near-infrared spectroscopy was used to classify soil types and predict their properties. Nonetheless, this technique required a spectrometer or visible near-infrared light source, and the process was cumbersome [15]. Furthermore, a complex algorithm is required to process the data, making traditional systems unsuitable for field detection.

Therefore, methods based on digital cameras, such as the proximal sensing of soil, have been developed. In particular, digital cameras were used to differentiate soil colors, and the RGB signals obtained were subsequently transformed into a standard color space through calculations [16]. Viscarra Rossel et al. [17] consider that the current developing course of proximal soil sensors is because of the surge of soil data for applications such as precision agriculture and dynamic models for monitoring environmental changes.

García et al. [18] discussed the recent on-site use of mobile phones to determine specific analyte concentrations from single-use chemically reactive membranes by considering how the hue changes from blue to magenta. The results indicated that mobile phones could be a solution for the increasing demand for objective soil color data approaches. Nevertheless, calorimetrically using mobile phones has not been tested to determine whether the contained color gamut can be compared to MCC. Therefore, contrary to dichotomous color choices, mobile phones differentiate between diverse reddish, brownish, and yellowish hues, from dark to light, and of variable intensity. The use of a smartphone app connecting a camera to perform image analysis and server-side processing for soil carbon estimation was demonstrated by Aitkenhead, M. J et al. [19].

The study used soil color as an essential indicator; the authors claimed that the method could be used to characterize, classify, and identify soil. According to Stiglitz et al. [20], Moonrungsee et al. [21], and Gomez-Robledo et al. [22], the use of mobile phone cameras to measure soil color is a promising alternative technique for classifying soil based on color. Similarly, Aitkenhead et al. [23] explained the use of smartphone-connected color sensors to conduct soil classification based on the soil color measured by these sensors. This method is convenient in terms of mobility. Unfortunately, the approach is camera-specific, requiring the calibration and testing of numerous camera sensors for individuals, which is unpractical. Moreover, control of lighting conditions at any given time is not known during the use of the app, increasing the likelihood of inconsistencies. Several studies, such as Gómez-Robledo, L. et al. [22], used smartphone cameras to measure soil color; the cameras were restrained to a controlled light source (i.e., controlled illumination condition) in the laboratory, whereas the approach followed by Stiglitz et al. [20] required a separate sensor. 

A recent study shows that machine learning and deep learning models can automate soil classification. Deep learning CNNs learn spatial and spectral information from high-resolution remote sensing data, improving accuracy and efficiency. Traditional machine learning techniques such as decision trees, random forests, support vector machines (SVM), k-nearest neighbors (k-NN), and naive Bayes have been extensively used to classify soil based on various input characteristics. Bhargavi et al. [24] identified agricultural soils using naive Bayes data mining. The naive Bayes classifier outperforms the Bayesian classifier. Kovacevic et al. [25] used a support vector machine to classify soil types based on profile sample’s chemical and physical attributes. Comparing logistic regression, multinomial naive Bayes, and SVM (linear and Gaussian) classification performance, researchers found that linear support vector machines could accurately automate soil classification. Linear SVM outperformed naïve Bayes with 57.61% accuracy. Barman et al. [26] used SVM to classify soils. Maniyath et al. [27] also classified soil by using k-nearest neighbor. Seybold et al. [28] estimated cation exchange capacity from organic C, clay, sediment, and soil pH using linear regression models. They initially sorted all data into exact soil-type groupings based on specified criteria. The stratification-obtained model parameters are related to the division of soil categories. Pham et al. [29] classified soil types using Adaboost models based on tree algorithm models. Pham collected 440 soil samples in total.

Considering the above literature review, we can conclude that significant efforts have been focused on soil identification. However, the previous approaches have limitations that motivate the development of improved soil identification methods that exhibit higher accuracy, precision, and efficiency than conventional methods. In this regard, deep learning based on computer vision technology is a promising alternative. Veres et al. [30] were the first to apply deep learning techniques to soil spectroscopy, where a 1D CNN proved notably effective for the estimation of some of the LUCAS soil properties. Liu et al. [31] used a 1D CNN with a distinct architecture to predict the clay content of the mineral soil samples of the LUCAS SSL and evaluated its suitability for transfer learning by fine tuning it for the organic soil samples and an airborne hyperspectral image. Padarian et al. [32] applied a 2D CNN to the LUCAS database, transforming the original spectra into 2D spectrograms. Transfer learning was also employed to localize the global model in both references, utilizing distinct techniques. Finally, Riese and Keller [33] classified the texture of each soil sample using the German soil textural classes by employing a second 1D CNN on the same dataset. N.L. Tsakiridis et al. [34] created and investigated the use of a one-dimensional convolutional neural network (1D CNN) to simultaneously predict ten physicochemical properties of the LUCAS SSL. Using a U-Net network model, Jiang et al. [35] classified 2400 soil samples into four classes. Jiang drew 2400 soil samples from 160 soil profile images of four soil orders (Alfisols, Entisols, Inceptisols, and Mollisols) that were collected in the Inner Mongolia and Liaoning regions of northern China. In a study by Azizi et al. [36], the InceptionV4, VGG16, and Resnet50 models were used to categorize six types of soil aggregates. To classify soil, Inazumi et al. [37] proposed a CNN model using 1060 images of clay, sand, and gravel. For simplification, he classified the soils as clay (D50 14 0.008 mm), sand (D50 14 0.7 mm), and gravel (D50 14 4 mm), with the water content set to zero. In clay, sand, and gravel, the particulate sizes were modified by sieving, placed in a clear plastic cup as a deviation from previous research, and obtained an accuracy of 86%. Zhong et al. [38] proposed Resnet and VGG16 CNN models for soil classification using the LUCAS soil dataset, categorized into four classes. Their model achieved relatively good accuracy by leveraging the rich spatial information encoded in the images. Barkataki et al. [39] also classified soil types from GPR B scans using deep learning techniques. 

Traditional soil classification methods frequently rely on labor-intensive and time-consuming field surveys and laboratory analyses, which can be expensive and limited in their spatial coverage. Few deep learning-based methods used for soil classifications have large model sizes, making their implementation on resource-constrained devices challenging. In addition to attaining a comparatively low accuracy percentage, they classify a few soil classes or categories. There is a need to develop a lightweight deep-learning model that can classify relatively large soil classes and, at the same time, strike a balance between model efficiency and accuracy and can also be implemented on a smartphone for real-time soil classification.

## 3. Materials and Methods

This section contains six subsections, including model architecture, model architecture components, data, data preprocessing, model training, and evaluation metrics.

### 3.1. Model Architecture

Soil-MobiNet is a condensed CNN model for soil classification derived from MobileNet. It is a convolutional neural network model using depthwise separable convolution as its basic unit, developed by Google. The architecture of Soil-MobiNet relies on the same depthwise separable convolutional design of the MobileNet model, also derived from Inception models [40], with an addition of three dense layers [41] following the depthwise and pointwise convolutions and the elimination of the last 1000 layers of the MobileNet model with neurons. Depthwise and pointwise convolutions constitute each depthwise separable convolution layer.

**Figure 1:**
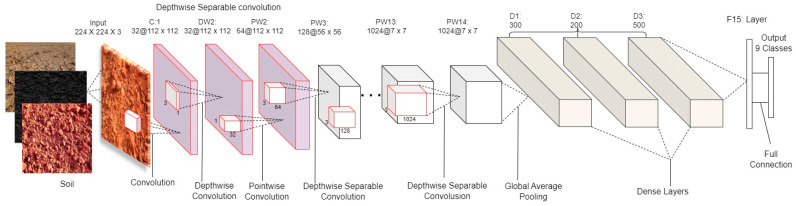
Soil-MobiNet Architecture.

MobileNet contains 28 layers if the depthwise and pointwise convolutions are counted separately. The width-multiplier hyper-parameter can be adjusted to reduce the number of parameters in a standard MobileNet, which has a standard 4.2 million parameters [42]. The input image was a 224 × 224 RGB channel. The architecture relies on a lightweight deep neural network such that the Mobile-net model can run in mobile applications. Therefore, Soil-MobiNet has a substantially lower number of parameters than current systems such as [43] and others [44]. Figure 1 shows a diagram of the Soil-MobiNet architecture.

### 3.2. Model’s Architecture Components

**Figure 2:**
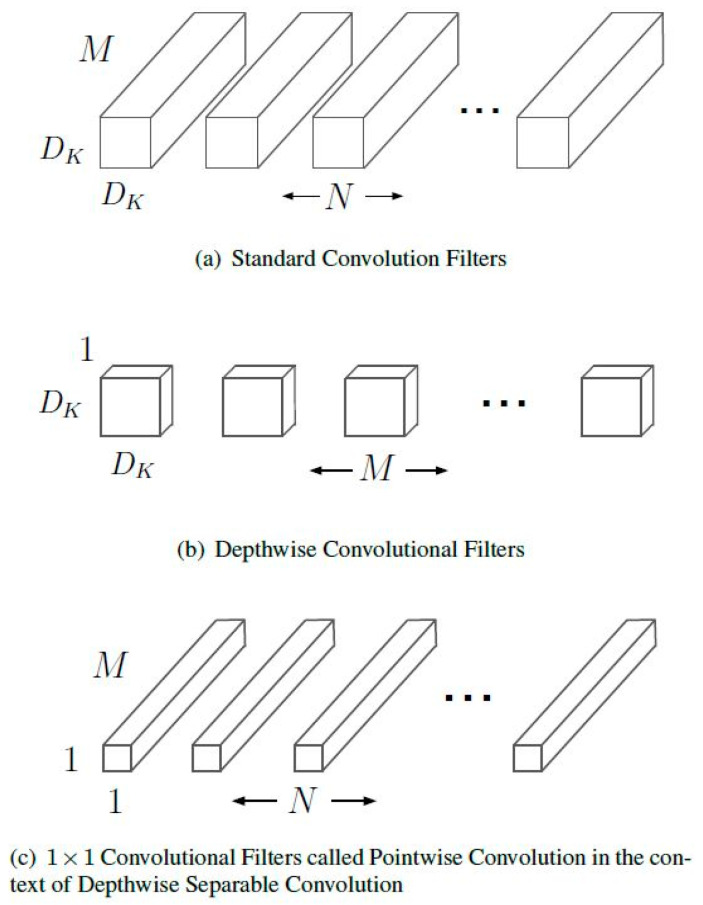
The standard convolutional filters in (**a**) are replaced by two layers: depthwise convolution in (**b**) and pointwise convolution in (**c**) to build a depthwise separable filter.

The MobileNet model is built on depthwise separable convolutions, a method of factorized convolutions that divides a normal convolution into a depthwise convolution and a 1×1 convolution known as a pointwise convolution. Depthwise convolution in MobileNets applies a single filter to each input channel. The depthwise convolution’s outputs are then combined using a 1×1 convolution by the pointwise convolution. In one step, a standard convolution of both filters combines the inputs into a new set of outputs [42]. This is divided into two layers by the depthwise separable convolution, one for filtering and one for merging. This factorization results in significant reductions in computation and model size. Figure 2 demonstrates the factorization of a normal convolution into a depthwise convolution and a pointwise convolution.

A standard convolutional layer takes a $D_{F}*D_{F}*M$ feature map *F* as input and produces a $D_{F}*D_{F}*N$ feature map *G*, with $D_{G}\;$ being the breadth of a square output feature map and spatial height, $D_{F}$ as the height of a square input feature map and spatial width. The number of output depth (output channels) is denoted by *N*, and *M* is the number of input depth (input channels). Convolution kernel *K* of size $D_{K}*D_{K}*M*N$ is used as a parameter for the standard convolutional layer, where $D_{K}$ is the spatial dimension of the kernel’s anticipated square shape. *M* is the number of input depth (input channels), and *N* is the number of output channels as mentioned earlier [42]. The output feature map for standard convolution is computed under the assumptions of stride one and padding as:

(1) $$G_{k,l,m}=\sum_{i,j,m}K_{i,j,m,n}\;*\;F_{k+i-1,l+j-1,m}\;\;\;\;\;\;\;\;$$

The cost of computation for standard convolution is:

(2) $$D_{K}*D_{K}*M*N*D_{F}*D_{F}\;\;\;\;\;\;\;\;\;\;$$

where the computational cost is multiplicatively dependent on the *M* input channels, *N* output channels, $D_{K}*D_{K}$ kernel size, and $D_{F}*D_{F}$ feature map size.

MobileNet models address each of these ideas and their connections. It begins by using depthwise separable convolutions to sever the connection between the quantity of output channels and the size of the kernel. Depending on the convolutional kernels, standard convolution operations have the impact of filtering features and merging features to produce a new representation. The filtration and combination phases can be split into two sections using factorized convolutions known as depthwise separable convolutions to significantly reduce computation costs. A depthwise separable convolution is made up of two layers: depthwise and pointwise convolutions. We use depthwise convolutions to apply a singular filter to each input depth (input channel). The result of the depthwise layer is then linearly combined using pointwise convolution, a regular 1×1 convolution. Batchnorm and ReLU nonlinearities are utilized in both levels of MobileNets.

For depthwise convolution with one filter per input channel, input depth is expressed as:

(3) $${\hat{G}}_{k,l,m}=\sum_{i,j,m}{\hat{K}}_{i,j,m,n}\;*F_{k+i-1,l+j-1,m}$$

where $\hat{K}$ is the depthwise convolutional kernel of dimension $D_{K}*D_{K}*M$ where the $m_{th}$ filter in $\hat{K}$ is applied to the $m_{th}$ channel in *F* to create the $m_{th}$ channel of the filtered output feature map $\hat{G}$. 

The computational cost of depthwise convolution is:

(4) $$D_{K}*D_{K}*M*D_{F}*D_{F}\;\;\;\;\;\;\;\;\;\;\;$$

In contrast to conventional convolution, depthwise convolution is exceedingly effective. However, it does not combine input channels to generate additional features; it just filters the input channels. To generate these additional features, a second layer that computes a linear combination of the results of depthwise convolution via 1×1 convolution is required. Depthwise separable convolution is the result of combining depthwise convolution with 1×1 (pointwise) convolution.

Depthwise separable convolutions cost:

(5) $$D_{K}*D_{K}*M*D_{F}*D_{F}+M*N*D_{F}*D_{F}\;\;\;\;\;\;\;\;\;\;$$

Thus, the summation of the depthwise and 1×1 pointwise convolutions. 

Convolution can be expressed as a two-step filtering and combining method, which results in a computation reduction of:

(6) $$\frac{D_{K}*D_{K}*M*D_{F}*D_{F}+M*N*D_{F}*D_{F}}{D_{K}*D_{K}*M*D_{F}*D_{F}}=\frac{1}{N}+\frac{1}{{D^{2}}_{K}}\;\;\;\;\;\;$$

Therefore, Soil-MobiNet makes use of the weight of this computation reduction and the weight of each of the soil image features extracted by the three additional dense layers introduced. 

This can be expressed as follows:

(7) $$\sum w\left(\frac{D_{K}*D_{K}*M*D_{F}*D_{F}+M*N*D_{F}*D_{F}}{D_{K}*D_{K}*M*D_{F}*D_{F}}=\frac{1}{N}+\frac{1}{{D^{2}}_{K}}\right)+w\left(D_{1}+D_{2}+D_{3}\right)\;\;\;\;\;\;$$

**Figure 3:**
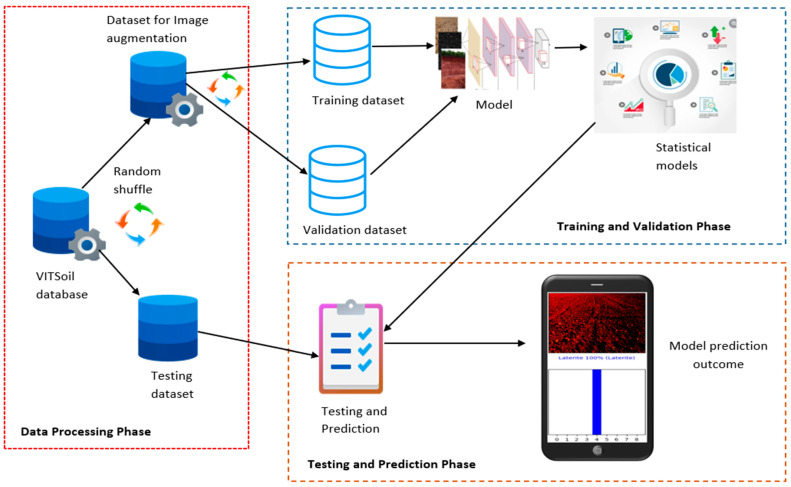
The framework of the modeling.

Figure 3 shows the framework of the modeling, which is divided into the data processing phase, training and validation phase, and testing and prediction phase. The data is randomly partitioned into two datasets, the test dataset and the dataset for image augmentation, during the data processing phase. The training and validation phase illustrates the training and validation of the model with the augmented training and validation dataset soil images. The last phase is the testing and prediction of the model.

### 3.3. Data

Data labeled by experts must be accessible to train any neural network in the supervised learning framework to perform classification. Thus, the primary step is to find adequate training samples [45,46]. Furthermore, building a larger dataset for training and testing improves classification accuracy significantly. Therefore, the size of the labeled datasets is crucial for the CNNs to function effectively and attain high performance. For instance, ImageNet [47] is the most well-known and extensive data collection platform, with over 10 million annotated pictures suitable for numerous image classification algorithms.

**Figure 4:**
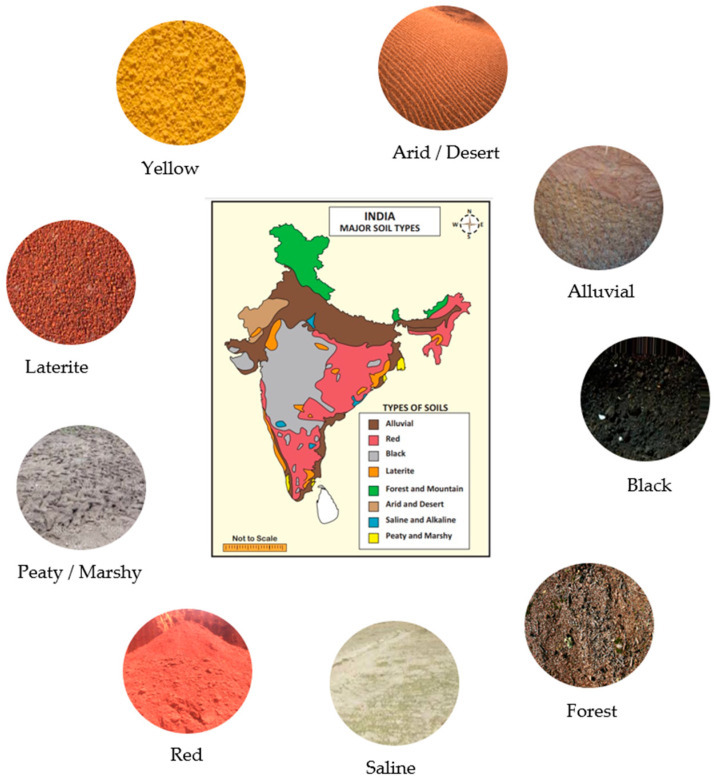
Map of India’s major soil types.

The Vellore Institute of Technology University soil dataset (VITSoil) contains nine distinct types of soil, that is, 4864 unique images with 224 × 224 pixels. The dataset comprises alluvial soil (*AL*), arid or desert soil (*AD*), black or regur soil (*BL*), forest soil (*FR*), laterite soil (*LA*), peaty or marshy soil (*PM*), saline soil (*SA*), red soil (*RE*), and yellow soil (*YE*). The images were captured from their various geographical locations regarding the soil map of India [48]. The labels were established by experienced professors of the soil science department at VIT University, India. Figure 4 shows the map of the geographical locations of the diverse soils.

For agricultural purposes, these are the primary soils classified by the National Bureau of Soil Survey and Land Use Planning (NBSS&LUP), a subsidiary of the Indian Council of Agricultural Research (ICAR) [48]. While the ICAR’s soil classification joins both red and yellow soils as one, the VITSoil dataset separates them for each to be uniquely recognized as they are very distinct. Table 1 summarizes these Indian soil morphologies and their geospatial location based on ICAR-NBSS&LUP criteria.

**Figure 5:**
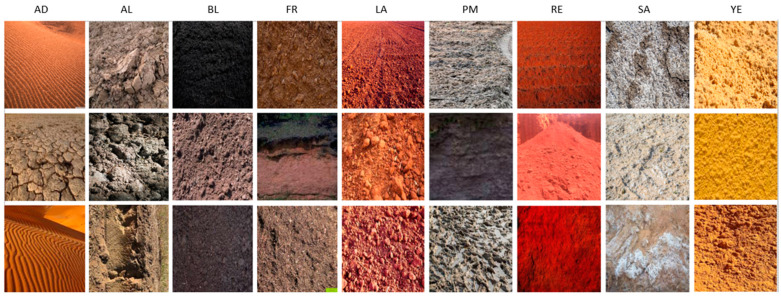
Samples of the nine categories of the VITSoil dataset, in columns from left; (*AD*): arid/desert soil, (*AL*): alluvial soil, (*BL*): black soil, (*FR*): forest soil, (*LA*): laterite soil, (*PM*): peaty/marshy soil, (*RE*): red soil, (*SA*): saline soil, (*YE*): yellow soil.

Figure 5 depicts representative samples of the nine VITSoil dataset categories. (*AD*): arid/desert soil, (*AL*): alluvial soil, (*BL*): black soil, (*FR*): forest soil, (*LA*): laterite soil, (*PM*): peaty/marshy soil, (*RE*): red soil, (*SA*): saline soil, (*YE*): yellow soil.

To prevent image overlap, the images were labeled by experienced professors of the soil science department at VIT University, India. Table 2 lists details of the VITSoil dataset.

### 3.4. Data Preprocessing

Finding a large number of correctly labeled images is essential to develop neural network models. Image data augmentation is a technique used to artificially increase the quantity of data in a dataset using a varied version of the dataset’s images. Numerous studies have shown the advantages of training a deep learning neural network model using a large dataset. That is, a larger dataset allows the development of improved models.

Several versions of images are created using augmentation approaches by increasing the capacity of the fit models to generalize what they have learned to new images. In the course of the research, we used Keras’ deep learning neural network library which includes the image data generator class, allowing us to fit models with image data augmentation. This image data generator class supports a wide range of pixel scaling techniques and approaches. To perform image zoom, shift, flip, and rotation, we frequently use the zoom-range, width-shift-range, height-shift-range, horizontal-flip, and rotation-range arguments. We reserved 360 samples from the 4864 VITSoil dataset at random for testing and then implemented the augmentation processes on the outstanding 4504 soil samples to create a unique test dataset that was not used for modeling. The augmentation process increased the 4504 soil samples to 37,869, resulting in a substantially large soil-image dataset for the experiments.

A neural network console software application developed by Sony Network Communication Inc. was used to process the images to ensure that the dataset had the same image size and format. This software resizes the images belonging to nine classes into 224 × 224-pixel RGB images and converts them to PNG format. To avoid overfitting the model, we randomly partitioned the 37,869 image samples into two parts: 70% training set and 30% validation set. The concept is to have three sets of data: one used to train the model (train), one used for validation purposes, such as hyperparameter tuning and model selection, and one used to perform a final model verification (test).

### 3.5. Model Training

Soil-MobiNet, similar to other CNN models, takes a soiled image as an input in the form of pixels and assigns significance (learnable-weights and biases) to various features of the image to distinguish one from the other. Soils have several textures that can be used to describe their appearance. However, some soil characteristics or properties, such as color (shade), are challenging to discern from one another because they sometimes look similar; for example, peaty soil and black soil; red soil and yellow soil. Therefore, directly using FC and convolutional layers to extract features from images would not provide high accuracy [48].

To address this limitation, we built a Soil-MobiNet based on three dense layers. Different filter sets are used to capture textures such as edges, spots, and patterns for an individual convolutional. To obtain a desired response for a particular pattern or texture, each filter was trained. For the same soil image, the feature maps of the convolutional layers presented various activation effects. Soil-MobiNet has internal structures meant to run on two-dimensional soil images and, hence, preserves the spatial relationships ascertained by the model.

**Figure 6:**
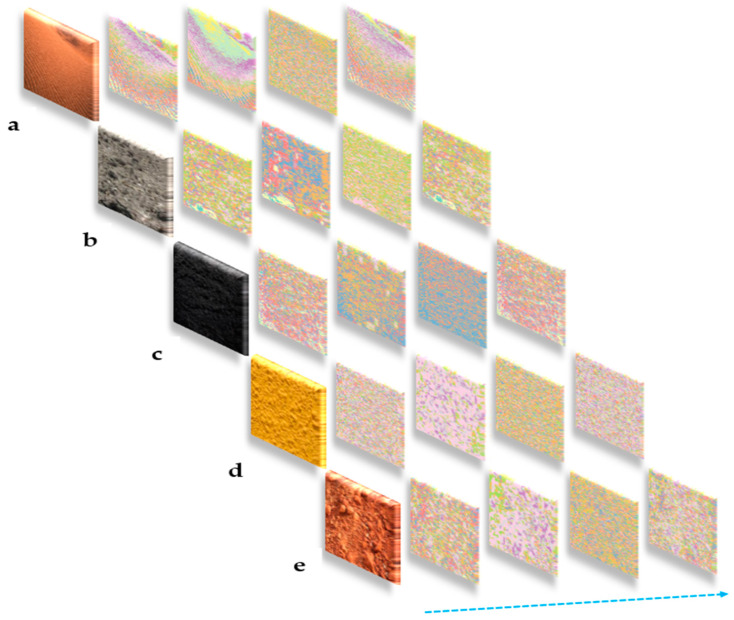
Feature maps of some VITSoil images as captured by the first convolutional layer of the model. From the top left; (**a**) arid soil, (**b**) alluvial soil, (**c**) black soil, (**d**) yellow soil, (**e**) laterite soil, with each having its feature maps on the right side.

The soil-type features that the Soil-MobiNet model identifies can be specifically found using the two-dimensional filters that the model learned. Additionally, the activation maps produced by the convolutional layers of Soil-MobiNet can be used to comprehend the precise features identified for a specific input soil image. The learned filters in neural networks are simple weights. Figure 6 shows samples of some of the soil images and their feature maps as captured by the model’s first convolutional layer. In particular, the training was not regularized by weight decay, and no dropouts were introduced. Table 3 and Table 4 outline the learning environment and values of the parameters used to train the model.

### 3.6. Evaluation Metric

We tested the efficacy of the model performance using the test dataset. In particular, we used the machine learning package Scikit-Learn’s syntax to construct the classification report: “from sklearn. metrics import classification report”. However, we describe the mathematical foundations of these metrics using four procedures to determine whether the predictions are accurate or inaccurate.

Accuracy, precision, and recall are computed as the evaluation metrics in this study to thoroughly assess the proposed method’s classification performance for diverse types of soil images.

True Negative (*tn*): Implies the case was negative and predicted negative.

True Positive (*tp*): Implies the case was positive and predicted positive.

False Negative (*fn*): Implies the case was positive but predicted negative.

False Positive (*fp*): Implies the case was negative but predicted positive.

Precision shows what percentage of the predictions are correct, that is, the ability of the Soil-MobiNet model not to label an instance positive that is negative. Precision is defined for each class as the ratio of a truly positive to the sum of a truly positive and false positive. 

Precision is calculated as follows:

(8) $$Precision\;(Pr)=\frac{tp}{\left(tp+fp\right)}\;\;\;\;\;\;\;\;$$

A recall is defined for each class as the ratio of true positives to the sum of true positives and false negatives. That is, what percentage of the positive cases has the model identified? Recall implies the fraction of positives that are correctly identified, which can be calculated as follows:

(9) $$Recall\;(Re)=\frac{tp}{tp+fn}\;\;\;\;\;\;\;\;\;\;\;\;\;\;\;\;$$

The *F*1 score is calculated as follows:

(10) $$F1-Score\left(\beta \right)=\frac{\left(1+\beta^{2}\right)tp}{\left(1+\beta^{2}\right)tp+\beta^{2}fp+fn}\;\;,\;\;$$

where β is set to 1.

Accuracy is calculated as follows:

(11) $$Accuracy\;(Acc)=\frac{\sum_{c}^{c}{tp}_{c}}{N},\;\;\;\;\;\;\;\;\;$$

Furthermore, support is the number of actual occurrences of the class in the specified dataset.

Macro average is calculated as follows:

(12) $$\beta_{macro}=\frac{1}{q}\sum_{\lambda =1}^{q}B\left({tp}_{\lambda },\;{fp}_{\lambda },\;{tn}_{\lambda },\;{fn}_{\lambda }\right)$$

Micro average or the weighted average is calculated as follows:

(13) $$\beta_{micro}=B\left(\sum_{\lambda =1}^{q}{tp}_{\lambda },\;\sum_{\lambda =1}^{q}{fp}_{\lambda },\;\sum_{\lambda =1}^{q}{tn}_{\lambda },\;\sum_{\lambda =1}^{q}{fn}_{\lambda }\right),\;\;$$

where $L=\lambda_{j},\;\left(j=1,\;\ldots ,\;q\right)$ is the set of labels, and B(tp, tn, fp, fn) is calculated based on the number of tp, tn, fp, and fn, respectively. Let ${tp}_{\lambda },\;{fp}_{\lambda },\;{tn}_{\lambda },$ and ${fn}_{\lambda }$ represent the number of tp, fp, tn, and fn after binary evaluation for a label *λ*.

## 4. Results

This section presents the acquisition of the results, which are in the form of tables, graphs, and images, and the discussion of the results.

### 4.1. Acquisition of Results

**Figure 7:**
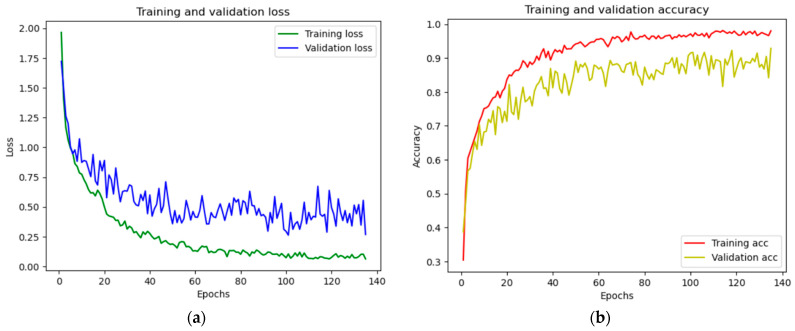
(**a**,**b**). Training and Validation Loss and Training and Validation Accuracy Graph. NB: Validation loss is a metric used to evaluate how well a deep learning model performed on the validation set. In other words, it is the loss calculated on the validation set when the data is divided into the train, validation, and test sets, whilst the validation set is a part of the dataset set aside to validate the model’s performance. The accuracy calculated on the dataset is not used for training but used during the training process to validate the generalizability of the model or for “early stopping”, known as validation accuracy.

From S1_Soil-MobiNet codes (Supplementary Material), our Soil-MobiNet model exhibited a training and validation accuracy of 98.47% and a training and validation loss of 0.0469, representing 4.69% after 4 h of training and validation. Figure 7a,b show a graph of training and validation loss (a) and training and validation accuracy (b).

**Figure 8:**
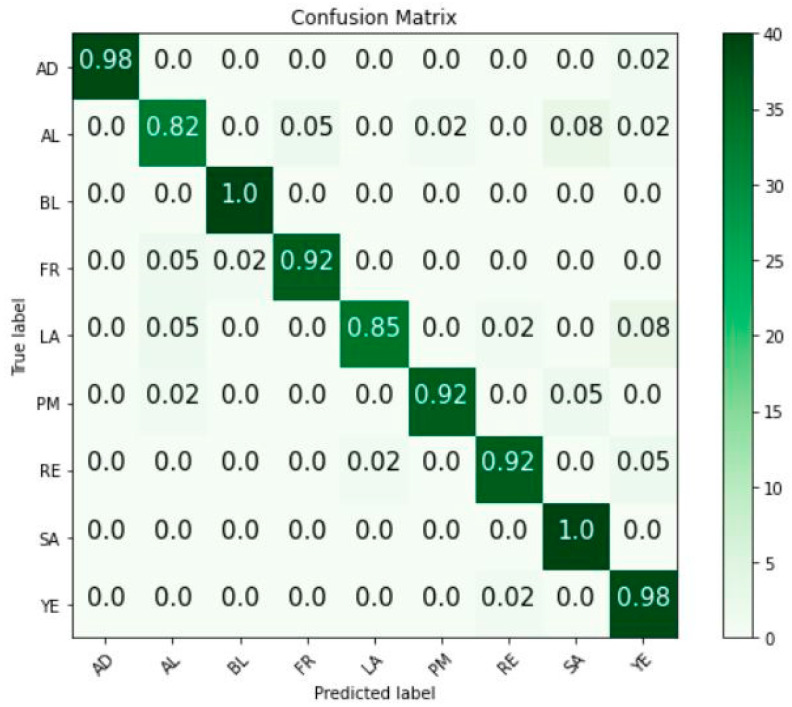
Confusion Matrix.

**Figure 9:**
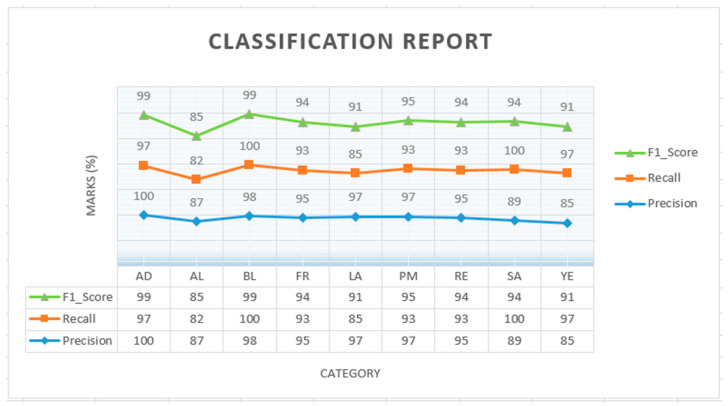
Graph of performance evaluation of categories in the VITSoil dataset.

Figure 8 illustrates the Soil-MobiNet model’s confusion matrix for the predicted classes. The confusion matrix describes the performance of our Soil-MobiNet model on the test data and demonstrates where the model can make accurate soil predictions alongside how the Soil-MobiNet model gets confused when making soil predictions. For instance, at some point, the model got confused in predicting the soil type of *SA* instead of *AL*. It is understandable since it is easy to mix the soil type of *AL* and *SA* due to their similarity in texture and color. The salt content of the soil type of *SA* is the only variance that soil science experts can use, in most cases, to distinguish between them. Figure 9 depicts a graph of the performance evaluation of categories in the VITSoil dataset, and Table 5 displays the VITSoil dataset’s performance evaluation measures.

### 4.2. Discussion of Results

In Figure 7, the training and validation accuracy graph of the Soil-MobiNet model for classifying the VITSoil dataset reveals how well the model performed overtime on the training and validation datasets. The 98.47% accuracy represents the percentage of correctly predicted labels relative to the total number of samples. During the early phases of training, both training and validation accuracy consistently increased, indicating that the model was learning and enhancing its ability to classify the data. The training accuracy tends to increase over time and ultimately reaches a plateau, whereas the validation accuracy also increases. This demonstrates how well the model suits the training data, as there was no indication that the model overfits the data. The training and validation loss graph reveals the model’s efficacy in terms of its ability to minimize the loss function during training. As the objective was to minimize this value, the loss represents the difference between the predicted and actual labels. Both training and validation loss was initially high because the model had not yet learned to make precise predictions. As training progressed, the loss values decreased as the model adjusted its internal parameters to closely match the training data. 

Figure 9 and Table 5 show a classification report which is a summary of the performance of a classification model, presented as a graph and a table, providing various metrics for each class in the dataset. They offer a detailed analysis of the model’s precision, recall, and F1 score for each class. The effectiveness of a model is wholly evaluated by examining both precision and recall and that of the F1 score. A graph of the performance assessment of the Soil-MobiNet model in categorizing soils from the VITSoil dataset is shown in Figure 9. In particular, the soil type of *AL* exhibited 82% recall but 87% precision, and the soil type of *YE* was 97% recall but 85% precision. The accuracies of the remaining soil classifications ranged from 89% to 100%. Table 5 is the weighted average or macro-average and the micro-average values of the classification report of the Soil-MobiNet model in classifying the VITSoil dataset. The weighted average considers the contribution of each class based on its support, while the macro-average treats all classes equally. The weighted average or macro-average values of the classification report provide an overall performance measure for the model. From the table, the Soil-MobiNet average score of 93% demonstrates the high model performance in classifying the nine soil classes. These results are good indicators of how well the proposed Soil-MobiNet model addresses soil classification, demonstrating great generalization, eliminating overfitting and underfitting during training, resilience, and accurately classifying the soil. The reliable dataset and the novel model we gathered and built resulted in a more accurate model. Thus, the Soil-MobiNet model was effective and efficient because only 135 epochs were required for a batch size of 27, substantially reducing costs and resources for computation such as time, energy, and memory. 

Figure 8 is the confusion matrix of the Soil-MobiNet model in classifying the VITSoil dataset. The confusion matrix shows where our model gets confused in classifying the VITSoil dataset. By analyzing the values in the confusion matrix and the derived metrics, we gain insights into the strengths and weaknesses of the model’s predictions, such as whether it tends to have more false positives or false negatives. For instance, confusing the soil type of *RE* and *YE* is understandable because they are mostly found mixed up in a particular location and, as a result, ICAR has even put them together as one soil. In the same way, confusion about the soil type *AL* and *SA* is not unusual since they are very identical in structure, texture, and color. The only difference that soil science experts can sometimes use to distinguish them is the salt content in *SA* soil. The non-zero off-diagonal elements (0.02, 0.05, and 0.08) in the confusion matrix represent the percentage of the few misclassifications of the model.

## 5. Implementation of Soil-MobiNet Model on Smartphone

The implementation of the Soil-MobiNet model on smartphones opens new possibilities for real-time monitoring, precision agriculture, and environmental studies, among other applications. It brings the benefits of accurate soil classification, morphology analysis, and geospatial location determination to the hands of users in a portable and efficient manner. The implementation process considered the preprocessing steps, input/output formats, and interaction with smartphone sensors for capturing geospatial information.

**Figure 10:**
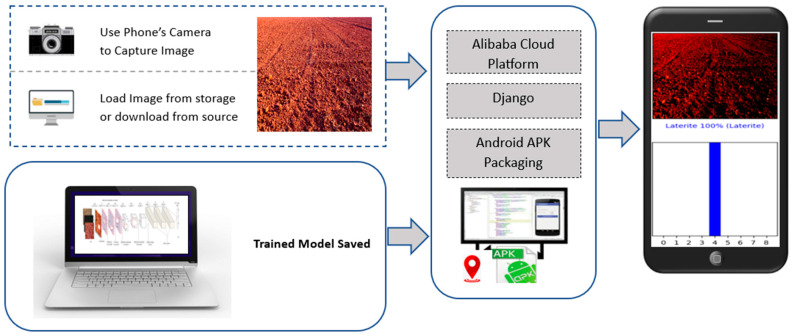
The framework of the Implementation of the Model on Smartphones.

After training and optimizing the Soil-MobiNet model, the saved model is then converted into a TensorFlow Lite format using the TensorFlow Lite converter. The TensorFlow Lite Converter is a command-line tool or a Python API that converts models from various TensorFlow formats (such as saved model, frozen graph, or checkpoint) to the TensorFlow Lite format. The model is then compiled into an Android application and deployed onto smartphones. The interface design allows users to choose between using their smartphone’s camera to capture real-time images of soil or selecting the soil image from their storage space. Before capturing the soil image, the user must enable the camera’s location settings to obtain the geospatial location information. The model on the back end of the app analyzes the image and predicts the type of soil taken or loaded from memory in real time. Figure 10 shows the steps to deploy the Soil-MobiNet model on the Android phone. Although the model can process and run successfully on the user’s smartphone because of its light weight and no need for a cloud server and internet connection, the cloud platform, which will require the use of an internet connection, will help save the downloaded soil image and soil type that the model predicted into the server’s database.

**Figure 11:**
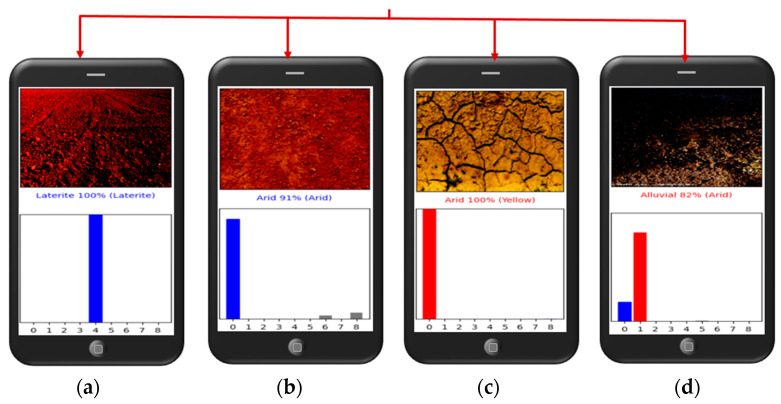
Four possible predictions anticipated.

Figure 11 shows the four possible predictions anticipated. (a) Blue bars in the predicted soil type indicate a 100% certainty that the model correctly identified the soil type as laterite soil. (b) Estimated soil type with grey and blue bar charts indicating 91% confidence that the model identified the soil type as arid soil and 9% as two other different soils. (c) Predicted soil type with a red bar chart indicating 100% certainty that the model mistook the genuine soil type for an arid one, whereas the actual soil type is yellow. (d) Predicted soil type with blue and red bar charts indicating 82% confidence in the model identifying the soil type as alluvial and 18% as arid soil, although the actual soil is not alluvial.

**Figure 12:**
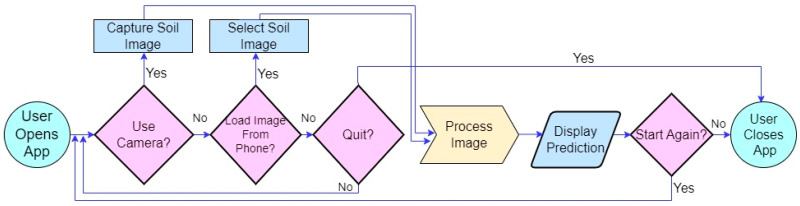
Flowchart of the soil App user interface design.

Similarly, Figure 12 shows the smartphone app user interface-design flowchart of the Soil-MobiNet model’s predictions on a smartphone.

Deep learning CNNs use features, such as texture, shape, patterns, and color, extracted from an image in the form of pixels and then assign weights and biases. Because the model has learned the features of the soil images under various lighting conditions and has provided exceptionally accurate findings, we expect factors, such as illumination conditions and the effect of white balance associated with the type of smartphone camera used, to have little or no impact on the predictions.

Unlike some traditional methods of soil identification, only color analysis is performed on images [16]. Although more beneficial to people, such as farmers, field workers, and pedologists, who are widely separated and frequently engage in soil activities, the developed app can be used by anyone interested in knowing the type of soil encountered. The uniqueness of this method of soil identification is that it does not require an expert user with prior knowledge of the subject. Unlike other traditional methods where experiments are conducted under controlled illumination conditions or in a closed environment, this method is not affected by illumination conditions. Moreover, the proposed approach is independent of the observer’s sensitivity, knowledge, experience, or color vision—qualities contrasting with most traditional methods such as the MCCs. 

## 6. Conclusions and Prospects

In this paper, we introduced Soil-MobiNet, a convolutional neural network model specifically designed for soil classification to determine soil morphology and its geospatial location. Leveraging the power of smartphones, Soil-MobiNet offers a portable and accessible solution for real-time soil analysis, empowering farmers, researchers, and land managers with valuable insights into soil characteristics. The development of Soil-MobiNet addresses the limitations of traditional manual techniques by automating the soil classification process. By analyzing soil images captured through smartphone cameras, the model effectively extracts complex spatial patterns and identifies different soil types based on their morphology. The integration of geospatial information further enhances the understanding of soil distribution and provides accurate location data, contributing to informed decision making. The results of our experiments and comparative analysis demonstrate the effectiveness and practicality of Soil-MobiNet. The model exhibits high accuracy in soil classification, outperforming existing methods and showcasing its potential for widespread adoption. Having a testing accuracy of 93% on average, and a training and validation accuracy of 98.47%, Soil-MobiNet showed outstanding performance in categorizing the VITSoil dataset. The model showed a few misclassifications between soil types of *RE* and *LA* and between soil types of *RE* and *YE*. Nevertheless, the proposed solution is practical because the soil pairings are nearly non-exclusive in terms of texture, structure, and color.

Although this is the first time classifying soils into nine categories as far as our memory serves us, and upon verification from the literature, the model’s 98.47% attained accuracy on nine classified soil classes supersedes several existing research on soil classification that only classified soils into a minimum of three and a maximum of seven categories. We believe that, with more training, the model can achieve an ideal accuracy of approximately 99%; future work will focus on this regard. Appendix A is a table of some soil classification performances with the latest technological approaches in the literature. Moreover, the optimization of Soil-MobiNet enables real-time inference on resource-constrained smartphone devices, ensuring that soil analysis can be conducted anytime and anywhere. 

The implications of Soil-MobiNet are significant across various domains. In agriculture, the accurate classification of soil types can help farmers make informed decisions about crop selection, fertilization strategies, and irrigation management. This leads to improved yields, reduced resource waste, and enhanced sustainability. In land management and urban planning, Soil-MobiNet aids in understanding soil properties for construction projects, identifying areas prone to erosion or contamination, and facilitating informed decisions regarding land use and zoning. The integration of geospatial information with soil classification offers additional benefits. By mapping the spatial distribution of different soil types, Soil-MobiNet contributes to comprehensive soil surveys and inventories. This information can guide land management practices, facilitate targeted soil conservation measures, and support environmental monitoring efforts. Furthermore, the geospatial data generated by Soil-MobiNet can be integrated with existing geographic information systems (GIS) and remote sensing technologies to create detailed soil maps and enhance the accuracy of land resource assessments. 

Despite the success and potential of Soil-MobiNet, there are a few limitations that should be acknowledged. Firstly, expanding the dataset used for training Soil-MobiNet with diverse soil samples from different regions can improve its generalizability and robustness. Future research can focus on increasing the soil classes. Although the model has been trained on soil images of varying light intensities and has proved to be resilient, once the model’s performance among other factors is dependent on the quality of soil images captured through smartphone cameras, factors such as lighting conditions, image resolution, and camera capabilities may occasionally impact the accuracy of soil classification. 

In conclusion, Soil-MobiNet represents a significant advancement in soil classification and analysis by harnessing the capabilities of convolutional neural networks and the ubiquity of smartphones. By providing an accessible, accurate, and real-time solution, Soil-MobiNet enables stakeholders to make informed decisions regarding soil management, land use planning, and environmental conservation. This research opens new avenues for the application of deep learning techniques in the field of soil science and paves the way for further advancements in mobile-based soil analysis. As technology continues to evolve, we envision a future where soil classification and analysis become seamlessly integrated into everyday smartphone applications, facilitating sustainable practices and ensuring the health of our ecosystems.

## Figures and Tables

**Table 1:** Summary of Indian Soil Morphology and its Geospatial Location.

Types	Main Distribution Area	Soil Characteristics	Texture	Color
Alluvial Soil	In the northern plains and river valleys, it is common. They are typically found in deltas and estuaries in peninsular India. Plains of the Indus-Ganga-Brahmaputra, Narmada-Tapi, Gujarat, Punjab, Haryana, Uttar Pradesh, Bihar, and Jharkhand, among others.	Organic materials, humus, and lime are all present. The soil is quite fruitful. They are depositional soils that are carried and deposited by rivers, streams, and other bodies of water. From west to east, the amount of sand in the land diminishes. Khadar refers to new alluvium, whereas Bhangar refers to ancient alluvium. Potash and lime are abundant, while phosphorus and nitrogen are scarce. Wheat, rice, maize, sugarcane, legumes, oilseeds, and other crops are mostly grown.	Sandy to silty loam or clay	Light Grey to Ash Grey
Red and Yellow Soil	Mostly found in low-rainfall environments. Orissa, Chhattisgarh, and the southern regions of the middle Ganga plain make up the eastern and southern parts of the Deccan plateau. The omnibus group is another name for this group.	Porous, friable structure. Lack of lime, kankar (impure calcium carbonate), and contains Ferric oxide. Deficient in Phosphate, Lime, Manganese, Nitrogen, Humus, and Potash. The lower layer is reddish-yellow or yellow. Wheat, cotton, pulses, tobacco, oilseeds, potato, etc., are cultivated.	Sandy to clay and loamy	Red
Black/Regur Soil	Black dirt covers the majority of the Deccan. Maharashtra, Madhya Pradesh, Gujarat, Andhra Pradesh, Tamil Nadu, Krishna Valleys, and the Godavari are all part of the Deccan plateau.	Soil that has matured, when wet, expands and becomes sticky, and when dry, it shrinks. When the black dirt dries, it creates broad fractures, which makes it self-plowing. Calcium, potassium, Iron, lime, aluminum, and magnesium are all abundant. Nitrogen, phosphorus, and organic matter are all in short supply. The ideal soil for growing cotton, rice, and other crops.	Clayey	Deep black to light black
Arid/Desert Soil	Arid and semi-arid conditions were observed. Western Rajasthan, North Gujarat, and Southern Punjab are all home to this species.	High salt content, a lack of moisture, and a high level of humus, kankar, or impure calcium carbonate all limit water entry. Phosphate is normal, while nitrogen is inadequate. Wind activities are primarily responsible for the deposition of this material.	Sandy	Red to Brown
Laterite Soil	The hills of Karnataka, Kerala, Tamil Nadu, Madhya Pradesh, Assam, and Orissa are home to this species. In locations where the temperature is high and there is a lot of rain. The name is derived from the Latin word "Later," which means "Brick."	As a result of excessive leaching. The soil will be leached of lime and silica. Bacteria will swiftly extract organic materials from the soil due to the high temperature, while trees and other plants will quickly consume hummus. As a result, the humus concentration is low. Iron and aluminum are abundant, while nitrogen, potash, potassium, lime, and humus are in little supply. When wet, they become extremely soft; yet when dry, they become extremely rigid. Rice, ragi, sugarcane, and cashew nuts are the most often grown crops.	Vary	Red
Saline Soil	Western Gujarat, the eastern coast deltas, and the Sunderban districts of West Bengal, Punjab, and Haryana are the most common locations. They may be found in dry and semi-arid climates, as well as in wet and marshy environments. Usara soils are another name for them.	Because saline soils have higher levels of salt, potassium, and magnesium, they are sterile and cannot support vegetative development. They have greater salt levels due to the dry climate and poor drainage. They are nitrogen and calcium deficient.	Sandy to Loamy	Dark Gray
Peaty/Marshy Soil	The northern section of Bihar, the southern half of Uttaranchal, and the coastal parts of West Bengal, Orissa, and Tamil Nadu are all places with a lot of rain and high humidity.	Vegetation growth is quite limited. The soil becomes alkaline when it contains a substantial amount of dead organic matter/humus. The earth is dark and heavy.	Vary	Black
Forest Soil	They occur in woodland regions when there is sufficient rainfall. In the Himalayas’ snow-covered regions.	The structure and texture of the soils vary depending on the mountain environment in which they are generated. On the valley sides, they are loamy and silty; while on the top slopes, they are coarse-grained, denuded, acidic, and low in humus. The soil in the lower valleys is nutrient-dense. Loamy and Silt.	Loamy and Silty Coarse-Grained	Light Brown

**Table 2:** Details of the VITSoil dataset.

Soil Name	Symbol	Initial Quantity	Augmented Quantity	Description
Arid/Desert	*AD*	489	3587	Deposited primarily by wind activities.
Alluvial	*AL*	433	3524	This is depositional soil transported by streams, rivers, etc.
Black/Regur	*BL*	579	3718	Mature soil with a high-water retention capacity.
Laterite	*LA*	561	3793	Created as a result of high leaching.
Peaty/Marshy	*PM*	579	3773	The growth of vegetation is very less. Heavy soil with black color.
Red	*RE*	502	3775	Porous, friable structure.
Saline	*SA*	586	3640	They are infertile and do not support any vegetative growth.
Yellow	*YE*	483	3747	Porous, friable structure. The lower layer is reddish-yellow or yellow.
Forest	*FR*	652	3808	Based on the mountain environment where they were produced, they vary in structure and texture.
	Total	4864	33,365	

Note: The abbreviations in the category are used to represent the types of soils.

**Table 3:** Training Environment.

Computer	Description
Model	Dell Inspiron 15
Processor	Intel(R) Core (TM) i7-7500U; CPU @ 2.70 GHz–2.90 GHz
RAM	12 GB
System	64-bit operating system
Windows	Windows 10 Education

**Table 4:** Values of the Parameters in the Proposed Soil-MobiNet Architecture.

Parameters				LR Scheduler and Parameters	
Proposed Model Name	Soil-MobiNet	Optimization	ADAM	Learning Rate	0.0001
Dataset	VITSoil	Framework	TensorFlow.Keras	Epoch	135
Used Software	Jupyter Notebook	Loss Type	Categorical Cross-entropy	Steps Per Epoch	44
Image Size	224 × 224	Activation function	ReLu Softmax	Avg. Epoch time	108 s
Batch Size	27				

**Table 5:** Performance evaluation measures for the VITSoil dataset.

$P_{macro}$	$R_{macro}$	$P_{micro}$	$R_{micro}$	Average
0.94	0.93	0.94	0.93	0.93

NB: $P_{macro}$ (precision macro average); $P_{micro}$ (precision micro average); $R_{macro}$ (recall macro average); $R_{micro}$ (recall micro average)

## Data Availability

The dataset will be made available upon reasonable request.

## Supplementary Materials

The following supporting information can be downloaded at: https://www.mdpi.com/article/10.3390/s23156709/s1, Supplementary Document S1_Soil-MobiNet_Codes.

## Appendix A

**Table A1:** Table of some soil classification performance with the latest technological approaches in the literature.

S. No.	Author (Year)	Image Modality	Datasets (Quantity)	Method (Model)	No. of Classes	Outcome (%)
1	Ours (2023)	VITSoil dataset	4864	Soil-MobiNet	9	Accuracy: 98.47% Precision: 94% Recall: 93%
2	Azizi et al., (2020) [36]	Soil Aggregates	Not specified	InceptionV4 VggNet16 ResNet50	6	Accuracy: 95.83%; 97.12%; 98.72% Precision: N/S Recall: N/S
3	Padarian et al., (2018) [32]	LUCAS Soil dataset	19,037	CNN	6	Accuracy: 87% Precision: N/S Recall: N/S
4	Inazumi et al., (2020) [37]	Sieved Laboratory soil	1060	CNN	3	Accuracy: 86% Precision: 77% Recall: N/S
5	Riese et al., (2019) [33]	LUCAS Soil dataset	16,076	CNN ResNet CoodNet	4	Accuracy: 71%, 72%, 73% Precision: 56%, 56%, 62% Recall: N/S
6	Jiang et al., (2021) [35]	Field Soil horizon	160	U-Net	3	Accuracy: 86% Precision: 82% Recall: N/S 83%
7	Barkataki et al., (2021) [39]	Synthetic GPR Soil Data	700	CNN	7	Accuracy: 97% Precision: 85% Recall: 92%
8	Zhong et al., (2021) [38]	LUCAS Soil dataset	17,939	LucasResNet16 LucasVGGNet16	4	Accuracy: 80.3%, 85.3% Precision: 74.9%, 76% Recall: N/S
