# Supplementary material for: Soil-MobiNet: A Convolutional Neural Network Model Base Soil Classification to Determine Soil Morphology and Its Geospatial Location
Source: Sensors (Basel). 2023 Jul 27;23(15):6709. doi: 10.3390/s23156709 (PMC10422283; doi:10.3390/s23156709)

In [17]:
```
import numpy as np
from tqdm import tqdm
import os
import cv2
import shutil
import random
import time
import matplotlib.pyplot as plt
%matplotlib inline
import tensorflow as tf
from tensorflow import keras
from keras import backend as K
from tensorflow.keras.layers import Dense, Dropout, Activation, Flatten,SpatialDropout
from tensorflow.keras.optimizers import Adam
from tensorflow.keras.metrics import categorical_crossentropy
from tensorflow.keras.preprocessing.image import ImageDataGenerator
from tensorflow.keras.preprocessing import image
from tensorflow.keras.models import Model
from tensorflow.keras.applications import imagenet_utils
from tensorflow. keras.applications import MobileNet
from tensorflow.keras.applications.mobilenet import preprocess_input
from IPython.display import Image
from sklearn.metrics import confusion_matrix
import itertools
import datetime
import time
```

In [18]:
```
mobile = tf.keras.applications.mobilenet.MobileNet()
```

In [19]:
```
mobile.summary()
```

```
Model: "mobilenet_1.00_224"
_________________________________________________________________
 Layer (type)                Output Shape              Param #   
=================================================================
 input_1 (InputLayer)        [(None, 224, 224, 3)]     0         
                                                                 
 conv1 (Conv2D)              (None, 112, 112, 32)      864       
                                                                 
 conv1_bn (BatchNormalizatio  (None, 112, 112, 32)     128       
 n)                                                              
                                                                 
 conv1_relu (ReLU)           (None, 112, 112, 32)      0         
                                                                 
 conv_dw_1 (DepthwiseConv2D)  (None, 112, 112, 32)     288       
                                                                 
 conv_dw_1_bn (BatchNormaliz  (None, 112, 112, 32)     128       
 ation)                                                          
                                                                 
 conv_dw_1_relu (ReLU)       (None, 112, 112, 32)      0         
                                                                 
 conv_pw_1 (Conv2D)          (None, 112, 112, 64)      2048      
                                                                 
 conv_pw_1_bn (BatchNormaliz  (None, 112, 112, 64)     256       
 ation)                                                          
                                                                 
 conv_pw_1_relu (ReLU)       (None, 112, 112, 64)      0         
                                                                 
 conv_pad_2 (ZeroPadding2D)  (None, 113, 113, 64)      0         
                                                                 
 conv_dw_2 (DepthwiseConv2D)  (None, 56, 56, 64)       576       
                                                                 
 conv_dw_2_bn (BatchNormaliz  (None, 56, 56, 64)       256       
 ation)                                                          
                                                                 
 conv_dw_2_relu (ReLU)       (None, 56, 56, 64)        0         
                                                                 
 conv_pw_2 (Conv2D)          (None, 56, 56, 128)       8192      
                                                                 
 conv_pw_2_bn (BatchNormaliz  (None, 56, 56, 128)      512       
 ation)                                                          
                                                                 
 conv_pw_2_relu (ReLU)       (None, 56, 56, 128)       0         
                                                                 
 conv_dw_3 (DepthwiseConv2D)  (None, 56, 56, 128)      1152      
                                                                 
 conv_dw_3_bn (BatchNormaliz  (None, 56, 56, 128)      512       
 ation)                                                          
                                                                 
 conv_dw_3_relu (ReLU)       (None, 56, 56, 128)       0         
                                                                 
 conv_pw_3 (Conv2D)          (None, 56, 56, 128)       16384     
                                                                 
 conv_pw_3_bn (BatchNormaliz  (None, 56, 56, 128)      512       
 ation)                                                          
                                                                 
 conv_pw_3_relu (ReLU)       (None, 56, 56, 128)       0         
                                                                 
 conv_pad_4 (ZeroPadding2D)  (None, 57, 57, 128)       0         
                                                                 
 conv_dw_4 (DepthwiseConv2D)  (None, 28, 28, 128)      1152      
```

```
conv_dw_4_bn (BatchNormaliz  (None, 28, 28, 128)      512
ation)

conv_dw_4_relu (ReLU)       (None, 28, 28, 128)      0

conv_pw_4 (Conv2D)          (None, 28, 28, 256)      32768

conv_pw_4_bn (BatchNormaliz  (None, 28, 28, 256)      1024
ation)

conv_pw_4_relu (ReLU)       (None, 28, 28, 256)      0

conv_dw_5 (DepthwiseConv2D)  (None, 28, 28, 256)      2304

conv_dw_5_bn (BatchNormaliz  (None, 28, 28, 256)      1024
ation)

conv_dw_5_relu (ReLU)       (None, 28, 28, 256)      0

conv_pw_5 (Conv2D)          (None, 28, 28, 256)      65536

conv_pw_5_bn (BatchNormaliz  (None, 28, 28, 256)      1024
ation)

conv_pw_5_relu (ReLU)       (None, 28, 28, 256)      0

conv_pad_6 (ZeroPadding2D)  (None, 29, 29, 256)      0

conv_dw_6 (DepthwiseConv2D)  (None, 14, 14, 256)      2304

conv_dw_6_bn (BatchNormaliz  (None, 14, 14, 256)      1024
ation)

conv_dw_6_relu (ReLU)       (None, 14, 14, 256)      0

conv_pw_6 (Conv2D)          (None, 14, 14, 512)      131072

conv_pw_6_bn (BatchNormaliz  (None, 14, 14, 512)      2048
ation)

conv_pw_6_relu (ReLU)       (None, 14, 14, 512)      0

conv_dw_7 (DepthwiseConv2D)  (None, 14, 14, 512)      4608

conv_dw_7_bn (BatchNormaliz  (None, 14, 14, 512)      2048
ation)

conv_dw_7_relu (ReLU)       (None, 14, 14, 512)      0

conv_pw_7 (Conv2D)          (None, 14, 14, 512)      262144

conv_pw_7_bn (BatchNormaliz  (None, 14, 14, 512)      2048
ation)

conv_pw_7_relu (ReLU)       (None, 14, 14, 512)      0

conv_dw_8 (DepthwiseConv2D)  (None, 14, 14, 512)      4608

conv_dw_8_bn (BatchNormaliz  (None, 14, 14, 512)      2048
```

```
ation)

conv_dw_8_relu (ReLU)       (None, 14, 14, 512)       0

conv_pw_8 (Conv2D)          (None, 14, 14, 512)       262144

conv_pw_8_bn (BatchNormaliz  (None, 14, 14, 512)      2048
ation)

conv_pw_8_relu (ReLU)       (None, 14, 14, 512)       0

conv_dw_9 (DepthwiseConv2D)  (None, 14, 14, 512)      4608

conv_dw_9_bn (BatchNormaliz  (None, 14, 14, 512)      2048
ation)

conv_dw_9_relu (ReLU)       (None, 14, 14, 512)       0

conv_pw_9 (Conv2D)          (None, 14, 14, 512)       262144

conv_pw_9_bn (BatchNormaliz  (None, 14, 14, 512)      2048
ation)

conv_pw_9_relu (ReLU)       (None, 14, 14, 512)       0

conv_dw_10 (DepthwiseConv2D  (None, 14, 14, 512)      4608
)

conv_dw_10_bn (BatchNormali  (None, 14, 14, 512)      2048
zation)

conv_dw_10_relu (ReLU)      (None, 14, 14, 512)       0

conv_pw_10 (Conv2D)         (None, 14, 14, 512)       262144

conv_pw_10_bn (BatchNormali  (None, 14, 14, 512)      2048
zation)

conv_pw_10_relu (ReLU)      (None, 14, 14, 512)       0

conv_dw_11 (DepthwiseConv2D  (None, 14, 14, 512)      4608
)

conv_dw_11_bn (BatchNormali  (None, 14, 14, 512)      2048
zation)

conv_dw_11_relu (ReLU)      (None, 14, 14, 512)       0

conv_pw_11 (Conv2D)         (None, 14, 14, 512)       262144

conv_pw_11_bn (BatchNormali  (None, 14, 14, 512)      2048
zation)

conv_pw_11_relu (ReLU)      (None, 14, 14, 512)       0

conv_pad_12 (ZeroPadding2D)  (None, 15, 15, 512)      0

conv_dw_12 (DepthwiseConv2D  (None, 7, 7, 512)        4608
)
```

```
 conv_dw_12_bn (BatchNormali  (None, 7, 7, 512)        2048
 zation)

 conv_dw_12_relu (ReLU)      (None, 7, 7, 512)         0

 conv_pw_12 (Conv2D)         (None, 7, 7, 1024)        524288

 conv_pw_12_bn (BatchNormali  (None, 7, 7, 1024)       4096
 zation)

 conv_pw_12_relu (ReLU)      (None, 7, 7, 1024)        0

 conv_dw_13 (DepthwiseConv2D  (None, 7, 7, 1024)       9216
 )

 conv_dw_13_bn (BatchNormali  (None, 7, 7, 1024)       4096
 zation)

 conv_dw_13_relu (ReLU)      (None, 7, 7, 1024)        0

 conv_pw_13 (Conv2D)         (None, 7, 7, 1024)        1048576

 conv_pw_13_bn (BatchNormali  (None, 7, 7, 1024)       4096
 zation)

 conv_pw_13_relu (ReLU)      (None, 7, 7, 1024)        0

 global_average_pooling2d (G  (None, 1, 1, 1024)       0
 lobalAveragePooling2D)

 dropout (Dropout)           (None, 1, 1, 1024)        0

 conv_preds (Conv2D)         (None, 1, 1, 1000)        1025000

 reshape_2 (Reshape)         (None, 1000)              0

 predictions (Activation)    (None, 1000)              0

=================================================================
Total params: 4,253,864
Trainable params: 4,231,976
Non-trainable params: 21,888
_________________________________________________________________
```

In [20]:

```
def prepare_image(file):
    img_path = ''
    img = image.load_img(img_path + file, target_size=(224, 224))
    img_array = image.img_to_array(img)
    img_array_expanded_dims = np.expand_dims(img_array, axis=0)
    return tf.keras.applications.mobilenet.preprocess_input(img_array_expanded_dims)
```

In [21]:

```
num_of_train_samples = 26508
num_of_valid_samples = 11361
num_of_test_samples = 360
```

In [22]:

```
#Show sample image
Image(filename='C:/Users/GyasiEmmanuelKwabena/Desktop/SOILNET/train/RE/RE_372.png', wi
```

Out[22]:

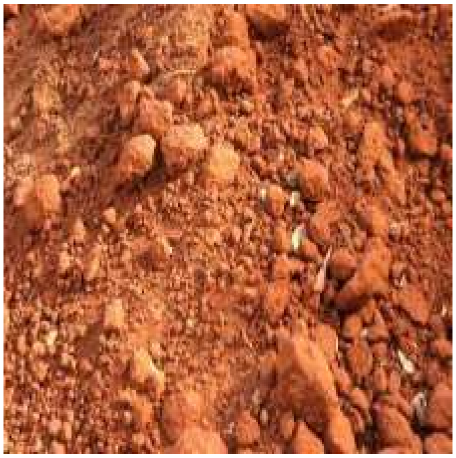

In [23]:

```
train_path = 'C:/Users/GyasiEmmanuelKwabena/Desktop/SOILNET/train'
valid_path = 'C:/Users/GyasiEmmanuelKwabena/Desktop/SOILNET/valid'
test_path = 'C:/Users/GyasiEmmanuelKwabena/Desktop/SOILNET/test'


train_batches = ImageDataGenerator(preprocessing_function=tf.keras.applications.mobile
    directory=train_path, target_size=(224,224), batch_size=22)
valid_batches = ImageDataGenerator(preprocessing_function=tf.keras.applications.mobile
    directory=valid_path, target_size=(224,224), batch_size=22)
test_batches = ImageDataGenerator(preprocessing_function=tf.keras.applications.mobilen
    directory=test_path, target_size=(224,224),batch_size=360, shuffle=False)

batchX, batchy = train_batches.next()
print('Batch shape=%s, min=%.3f, max=%.3f' % (batchX.shape, batchX.min(), batchX.max(
```

```
Found 26508 images belonging to 9 classes.
Found 11361 images belonging to 9 classes.
Found 360 images belonging to 9 classes.
Batch shape=(22, 224, 224, 3), min=-1.000, max=1.000
```

In [24]:

```
# Modify the model: Mobilenet

base_model=MobileNet(weights='imagenet',include_top=False) #imports the mobilenet mode

x=base_model.output
x=GlobalAveragePooling2D()(x)
x=Dense(300,activation='relu')(x) # 300 we add dense layers so that the model can lear
x=Dense(200,activation='relu')(x) # 200 dense layer 2
x=Dense(512,activation='relu')(x) # 512 dense layer 3
preds=Dense(9,activation='softmax')(x) #final layer with softmax activation
```

```
WARNING:tensorflow:`input_shape` is undefined or non-square, or `rows` is not in [12
8, 160, 192, 224]. Weights for input shape (224, 224) will be loaded as the default.
```

In [25]:

```
for i,layer in enumerate(base_model.layers):
    print(i,layer.name)
```

```
0 input_2
1 conv1
2 conv1_bn
3 conv1_relu
4 conv_dw_1
5 conv_dw_1_bn
6 conv_dw_1_relu
7 conv_pw_1
8 conv_pw_1_bn
9 conv_pw_1_relu
10 conv_pad_2
11 conv_dw_2
12 conv_dw_2_bn
13 conv_dw_2_relu
14 conv_pw_2
15 conv_pw_2_bn
16 conv_pw_2_relu
17 conv_dw_3
18 conv_dw_3_bn
19 conv_dw_3_relu
20 conv_pw_3
21 conv_pw_3_bn
22 conv_pw_3_relu
23 conv_pad_4
24 conv_dw_4
25 conv_dw_4_bn
26 conv_dw_4_relu
27 conv_pw_4
28 conv_pw_4_bn
29 conv_pw_4_relu
30 conv_dw_5
31 conv_dw_5_bn
32 conv_dw_5_relu
33 conv_pw_5
34 conv_pw_5_bn
35 conv_pw_5_relu
36 conv_pad_6
37 conv_dw_6
38 conv_dw_6_bn
39 conv_dw_6_relu
40 conv_pw_6
41 conv_pw_6_bn
42 conv_pw_6_relu
43 conv_dw_7
44 conv_dw_7_bn
45 conv_dw_7_relu
46 conv_pw_7
47 conv_pw_7_bn
48 conv_pw_7_relu
49 conv_dw_8
50 conv_dw_8_bn
51 conv_dw_8_relu
52 conv_pw_8
53 conv_pw_8_bn
54 conv_pw_8_relu
55 conv_dw_9
56 conv_dw_9_bn
57 conv_dw_9_relu
58 conv_pw_9
59 conv_pw_9_bn
```

```
60 conv_pw_9_relu
61 conv_dw_10
62 conv_dw_10_bn
63 conv_dw_10_relu
64 conv_pw_10
65 conv_pw_10_bn
66 conv_pw_10_relu
67 conv_dw_11
68 conv_dw_11_bn
69 conv_dw_11_relu
70 conv_pw_11
71 conv_pw_11_bn
72 conv_pw_11_relu
73 conv_pad_12
74 conv_dw_12
75 conv_dw_12_bn
76 conv_dw_12_relu
77 conv_pw_12
78 conv_pw_12_bn
79 conv_pw_12_relu
80 conv_dw_13
81 conv_dw_13_bn
82 conv_dw_13_relu
83 conv_pw_13
84 conv_pw_13_bn
85 conv_pw_13_relu
```

In [26]:
```
#we want to set the first 20 layers of the network to be non-trainable
for layer in base_model.layers[:20]:
    layer.trainable=False
```

In [27]:
```
model=Model(inputs=base_model.input,outputs=preds)
```

In [28]:
```
model.summary()
```

```
Model: "model"
_________________________________________________________________
 Layer (type)                Output Shape              Param #   
=================================================================
 input_2 (InputLayer)        [(None, None, None, 3)]   0         
                                                                 
 conv1 (Conv2D)              (None, None, None, 32)    864       
                                                                 
 conv1_bn (BatchNormalizatio  (None, None, None, 32)   128       
 n)                                                              
                                                                 
 conv1_relu (ReLU)           (None, None, None, 32)    0         
                                                                 
 conv_dw_1 (DepthwiseConv2D)  (None, None, None, 32)   288       
                                                                 
 conv_dw_1_bn (BatchNormaliz  (None, None, None, 32)   128       
 ation)                                                          
                                                                 
 conv_dw_1_relu (ReLU)       (None, None, None, 32)    0         
                                                                 
 conv_pw_1 (Conv2D)          (None, None, None, 64)    2048      
                                                                 
 conv_pw_1_bn (BatchNormaliz  (None, None, None, 64)   256       
 ation)                                                          
                                                                 
 conv_pw_1_relu (ReLU)       (None, None, None, 64)    0         
                                                                 
 conv_pad_2 (ZeroPadding2D)  (None, None, None, 64)    0         
                                                                 
 conv_dw_2 (DepthwiseConv2D)  (None, None, None, 64)   576       
                                                                 
 conv_dw_2_bn (BatchNormaliz  (None, None, None, 64)   256       
 ation)                                                          
                                                                 
 conv_dw_2_relu (ReLU)       (None, None, None, 64)    0         
                                                                 
 conv_pw_2 (Conv2D)          (None, None, None, 128)   8192      
                                                                 
 conv_pw_2_bn (BatchNormaliz  (None, None, None, 128)  512       
 ation)                                                          
                                                                 
 conv_pw_2_relu (ReLU)       (None, None, None, 128)   0         
                                                                 
 conv_dw_3 (DepthwiseConv2D)  (None, None, None, 128)  1152      
                                                                 
 conv_dw_3_bn (BatchNormaliz  (None, None, None, 128)  512       
 ation)                                                          
                                                                 
 conv_dw_3_relu (ReLU)       (None, None, None, 128)   0         
                                                                 
 conv_pw_3 (Conv2D)          (None, None, None, 128)   16384     
                                                                 
 conv_pw_3_bn (BatchNormaliz  (None, None, None, 128)  512       
 ation)                                                          
                                                                 
 conv_pw_3_relu (ReLU)       (None, None, None, 128)   0         
                                                                 
 conv_pad_4 (ZeroPadding2D)  (None, None, None, 128)   0         
                                                                 
 conv_dw_4 (DepthwiseConv2D)  (None, None, None, 128)  1152      
```

```
conv_dw_4_bn (BatchNormaliz  (None, None, None, 128)  512
ation)

conv_dw_4_relu (ReLU)       (None, None, None, 128)   0

conv_pw_4 (Conv2D)          (None, None, None, 256)   32768

conv_pw_4_bn (BatchNormaliz  (None, None, None, 256)  1024
ation)

conv_pw_4_relu (ReLU)       (None, None, None, 256)   0

conv_dw_5 (DepthwiseConv2D)  (None, None, None, 256)  2304

conv_dw_5_bn (BatchNormaliz  (None, None, None, 256)  1024
ation)

conv_dw_5_relu (ReLU)       (None, None, None, 256)   0

conv_pw_5 (Conv2D)          (None, None, None, 256)   65536

conv_pw_5_bn (BatchNormaliz  (None, None, None, 256)  1024
ation)

conv_pw_5_relu (ReLU)       (None, None, None, 256)   0

conv_pad_6 (ZeroPadding2D)  (None, None, None, 256)   0

conv_dw_6 (DepthwiseConv2D)  (None, None, None, 256)  2304

conv_dw_6_bn (BatchNormaliz  (None, None, None, 256)  1024
ation)

conv_dw_6_relu (ReLU)       (None, None, None, 256)   0

conv_pw_6 (Conv2D)          (None, None, None, 512)   131072

conv_pw_6_bn (BatchNormaliz  (None, None, None, 512)  2048
ation)

conv_pw_6_relu (ReLU)       (None, None, None, 512)   0

conv_dw_7 (DepthwiseConv2D)  (None, None, None, 512)  4608

conv_dw_7_bn (BatchNormaliz  (None, None, None, 512)  2048
ation)

conv_dw_7_relu (ReLU)       (None, None, None, 512)   0

conv_pw_7 (Conv2D)          (None, None, None, 512)   262144

conv_pw_7_bn (BatchNormaliz  (None, None, None, 512)  2048
ation)

conv_pw_7_relu (ReLU)       (None, None, None, 512)   0

conv_dw_8 (DepthwiseConv2D)  (None, None, None, 512)  4608

conv_dw_8_bn (BatchNormaliz  (None, None, None, 512)  2048
```

```
ation)

conv_dw_8_relu (ReLU)       (None, None, None, 512)   0

conv_pw_8 (Conv2D)          (None, None, None, 512)   262144

conv_pw_8_bn (BatchNormaliz  (None, None, None, 512)  2048
ation)

conv_pw_8_relu (ReLU)       (None, None, None, 512)   0

conv_dw_9 (DepthwiseConv2D)  (None, None, None, 512)  4608

conv_dw_9_bn (BatchNormaliz  (None, None, None, 512)  2048
ation)

conv_dw_9_relu (ReLU)       (None, None, None, 512)   0

conv_pw_9 (Conv2D)          (None, None, None, 512)   262144

conv_pw_9_bn (BatchNormaliz  (None, None, None, 512)  2048
ation)

conv_pw_9_relu (ReLU)       (None, None, None, 512)   0

conv_dw_10 (DepthwiseConv2D  (None, None, None, 512)  4608
)

conv_dw_10_bn (BatchNormali  (None, None, None, 512)  2048
zation)

conv_dw_10_relu (ReLU)      (None, None, None, 512)   0

conv_pw_10 (Conv2D)         (None, None, None, 512)   262144

conv_pw_10_bn (BatchNormali  (None, None, None, 512)  2048
zation)

conv_pw_10_relu (ReLU)      (None, None, None, 512)   0

conv_dw_11 (DepthwiseConv2D  (None, None, None, 512)  4608
)

conv_dw_11_bn (BatchNormali  (None, None, None, 512)  2048
zation)

conv_dw_11_relu (ReLU)      (None, None, None, 512)   0

conv_pw_11 (Conv2D)         (None, None, None, 512)   262144

conv_pw_11_bn (BatchNormali  (None, None, None, 512)  2048
zation)

conv_pw_11_relu (ReLU)      (None, None, None, 512)   0

conv_pad_12 (ZeroPadding2D)  (None, None, None, 512)  0

conv_dw_12 (DepthwiseConv2D  (None, None, None, 512)  4608
)
```

```
 conv_dw_12_bn (BatchNormali  (None, None, None, 512)  2048
 zation)

 conv_dw_12_relu (ReLU)      (None, None, None, 512)   0

 conv_pw_12 (Conv2D)         (None, None, None, 1024)  524288

 conv_pw_12_bn (BatchNormali  (None, None, None, 1024)  4096
 zation)

 conv_pw_12_relu (ReLU)      (None, None, None, 1024)  0

 conv_dw_13 (DepthwiseConv2D  (None, None, None, 1024)  9216
 )

 conv_dw_13_bn (BatchNormali  (None, None, None, 1024)  4096
 zation)

 conv_dw_13_relu (ReLU)      (None, None, None, 1024)  0

 conv_pw_13 (Conv2D)         (None, None, None, 1024)  1048576

 conv_pw_13_bn (BatchNormali  (None, None, None, 1024)  4096
 zation)

 conv_pw_13_relu (ReLU)      (None, None, None, 1024)  0

 global_average_pooling2d_1   (None, 1024)             0
 (GlobalAveragePooling2D)

 dense (Dense)               (None, 300)               307500

 dense_1 (Dense)             (None, 200)               60200

 dense_2 (Dense)             (None, 512)               102912

 dense_3 (Dense)             (None, 9)                 4617

=================================================================
Total params: 3,704,093
Trainable params: 3,668,189
Non-trainable params: 35,904
_________________________________________________________________
```

In [29]:

```python
train_datagen=ImageDataGenerator(preprocessing_function=preprocess_input) #included ir

train_generator=train_datagen.flow_from_directory(train_path,
                                                 target_size=(224, 224),
                                                 color_mode='rgb',
                                                 batch_size=27,
                                                 class_mode='categorical',
                                                 shuffle=True)



test_datagen = ImageDataGenerator()


validation_generator = test_datagen.flow_from_directory(valid_path,
                                                        target_size=(224, 224),
                                                        color_mode='rgb',
```

```
                                                        batch_size=27,
                                                        class_mode='categorical',
                                                        shuffle=True)
```

```
Found 26508 images belonging to 9 classes.
Found 11361 images belonging to 9 classes.
```

In [30]:
```
#Images Classes with index
print(train_generator.class_indices)
```

```
{'AD': 0, 'AL': 1, 'BL': 2, 'FR': 3, 'LA': 4, 'PM': 5, 'RE': 6, 'SA': 7, 'YE': 8}
```

In [31]:
```
model.compile(optimizer=Adam(learning_rate=0.0001), loss='categorical_crossentropy', m
```

In [32]:
```
start = datetime.datetime.now()

history = model.fit_generator(generator=train_generator,
            steps_per_epoch=len(train_batches)//train_generator.batch_size,
            validation_data=valid_batches,
            validation_steps=len(valid_batches)//valid_batches.batch_size,
            epochs=135,
            verbose=2
            )

end= datetime.datetime.now()
elapsed= end-start
print ('Time: ', elapsed)
```

```
C:\Users\GyasiEmmanuelKwabena\AppData\Local\Temp\ipykernel_1972\700373186.py:3: UserW
arning: `Model.fit_generator` is deprecated and will be removed in a future version.
Please use `Model.fit`, which supports generators.
  history = model.fit_generator(generator=train_generator,
```

```
Epoch 1/135
44/44 - 115s - loss: 1.9643 - accuracy: 0.3039 - val_loss: 1.7217 - val_accuracy: 0.3
874 - 115s/epoch - 3s/step
Epoch 2/135
44/44 - 112s - loss: 1.4124 - accuracy: 0.5059 - val_loss: 1.5005 - val_accuracy: 0.4
723 - 112s/epoch - 3s/step
Epoch 3/135
44/44 - 111s - loss: 1.1662 - accuracy: 0.6044 - val_loss: 1.2622 - val_accuracy: 0.5
672 - 111s/epoch - 3s/step
Epoch 4/135
44/44 - 111s - loss: 1.0615 - accuracy: 0.6246 - val_loss: 1.2001 - val_accuracy: 0.5
751 - 111s/epoch - 3s/step
Epoch 5/135
44/44 - 107s - loss: 0.9944 - accuracy: 0.6448 - val_loss: 1.0084 - val_accuracy: 0.6
186 - 107s/epoch - 2s/step
Epoch 6/135
44/44 - 108s - loss: 0.9571 - accuracy: 0.6650 - val_loss: 0.9442 - val_accuracy: 0.6
542 - 108s/epoch - 2s/step
Epoch 7/135
44/44 - 108s - loss: 0.8624 - accuracy: 0.6853 - val_loss: 0.9788 - val_accuracy: 0.6
304 - 108s/epoch - 2s/step
Epoch 8/135
44/44 - 113s - loss: 0.8390 - accuracy: 0.7130 - val_loss: 0.8818 - val_accuracy: 0.7
036 - 113s/epoch - 3s/step
Epoch 9/135
44/44 - 107s - loss: 0.7848 - accuracy: 0.7290 - val_loss: 1.0707 - val_accuracy: 0.6
423 - 107s/epoch - 2s/step
Epoch 10/135
44/44 - 107s - loss: 0.7739 - accuracy: 0.7508 - val_loss: 0.8730 - val_accuracy: 0.6
818 - 107s/epoch - 2s/step
Epoch 11/135
44/44 - 107s - loss: 0.7287 - accuracy: 0.7534 - val_loss: 0.8889 - val_accuracy: 0.6
838 - 107s/epoch - 2s/step
Epoch 12/135
44/44 - 108s - loss: 0.6927 - accuracy: 0.7584 - val_loss: 0.8824 - val_accuracy: 0.7
194 - 108s/epoch - 2s/step
Epoch 13/135
44/44 - 108s - loss: 0.6444 - accuracy: 0.7719 - val_loss: 0.8186 - val_accuracy: 0.7
095 - 108s/epoch - 2s/step
Epoch 14/135
44/44 - 107s - loss: 0.6160 - accuracy: 0.7828 - val_loss: 0.7536 - val_accuracy: 0.7
451 - 107s/epoch - 2s/step
Epoch 15/135
44/44 - 108s - loss: 0.6192 - accuracy: 0.7854 - val_loss: 0.9394 - val_accuracy: 0.6
739 - 108s/epoch - 2s/step
Epoch 16/135
44/44 - 108s - loss: 0.5918 - accuracy: 0.8022 - val_loss: 0.7183 - val_accuracy: 0.7
569 - 108s/epoch - 2s/step
Epoch 17/135
44/44 - 107s - loss: 0.6400 - accuracy: 0.7826 - val_loss: 0.6831 - val_accuracy: 0.7
510 - 107s/epoch - 2s/step
Epoch 18/135
44/44 - 108s - loss: 0.6112 - accuracy: 0.8022 - val_loss: 0.8861 - val_accuracy: 0.7
095 - 108s/epoch - 2s/step
Epoch 19/135
44/44 - 107s - loss: 0.5661 - accuracy: 0.8106 - val_loss: 0.8022 - val_accuracy: 0.7
431 - 107s/epoch - 2s/step
Epoch 20/135
44/44 - 108s - loss: 0.4987 - accuracy: 0.8367 - val_loss: 0.8896 - val_accuracy: 0.7
134 - 108s/epoch - 2s/step
```

```
Epoch 21/135
44/44 - 108s - loss: 0.4393 - accuracy: 0.8502 - val_loss: 0.5767 - val_accuracy: 0.8
221 - 108s/epoch - 2s/step
Epoch 22/135
44/44 - 107s - loss: 0.4240 - accuracy: 0.8476 - val_loss: 0.7681 - val_accuracy: 0.7
411 - 107s/epoch - 2s/step
Epoch 23/135
44/44 - 108s - loss: 0.4182 - accuracy: 0.8586 - val_loss: 0.7298 - val_accuracy: 0.7
332 - 108s/epoch - 2s/step
Epoch 24/135
44/44 - 108s - loss: 0.4127 - accuracy: 0.8645 - val_loss: 0.6072 - val_accuracy: 0.7
846 - 108s/epoch - 2s/step
Epoch 25/135
44/44 - 107s - loss: 0.3858 - accuracy: 0.8636 - val_loss: 0.8269 - val_accuracy: 0.7
194 - 107s/epoch - 2s/step
Epoch 26/135
44/44 - 108s - loss: 0.3881 - accuracy: 0.8729 - val_loss: 0.6657 - val_accuracy: 0.7
747 - 108s/epoch - 2s/step
Epoch 27/135
44/44 - 107s - loss: 0.3408 - accuracy: 0.8923 - val_loss: 0.5421 - val_accuracy: 0.8
142 - 107s/epoch - 2s/step
Epoch 28/135
44/44 - 108s - loss: 0.3481 - accuracy: 0.8855 - val_loss: 0.6279 - val_accuracy: 0.7
708 - 108s/epoch - 2s/step
Epoch 29/135
44/44 - 107s - loss: 0.3810 - accuracy: 0.8729 - val_loss: 0.6358 - val_accuracy: 0.7
767 - 107s/epoch - 2s/step
Epoch 30/135
44/44 - 108s - loss: 0.3135 - accuracy: 0.8889 - val_loss: 0.6309 - val_accuracy: 0.7
866 - 108s/epoch - 2s/step
Epoch 31/135
44/44 - 108s - loss: 0.3373 - accuracy: 0.8813 - val_loss: 0.6847 - val_accuracy: 0.7
589 - 108s/epoch - 2s/step
Epoch 32/135
44/44 - 108s - loss: 0.3234 - accuracy: 0.8889 - val_loss: 0.6742 - val_accuracy: 0.8
024 - 108s/epoch - 2s/step
Epoch 33/135
44/44 - 107s - loss: 0.2825 - accuracy: 0.9057 - val_loss: 0.5435 - val_accuracy: 0.8
202 - 107s/epoch - 2s/step
Epoch 34/135
44/44 - 108s - loss: 0.2921 - accuracy: 0.8939 - val_loss: 0.5156 - val_accuracy: 0.8
281 - 108s/epoch - 2s/step
Epoch 35/135
44/44 - 108s - loss: 0.2644 - accuracy: 0.9150 - val_loss: 0.5092 - val_accuracy: 0.8
458 - 108s/epoch - 2s/step
Epoch 36/135
44/44 - 108s - loss: 0.2404 - accuracy: 0.9276 - val_loss: 0.6046 - val_accuracy: 0.8
103 - 108s/epoch - 2s/step
Epoch 37/135
44/44 - 107s - loss: 0.2901 - accuracy: 0.9015 - val_loss: 0.5524 - val_accuracy: 0.8
123 - 107s/epoch - 2s/step
Epoch 38/135
44/44 - 107s - loss: 0.2687 - accuracy: 0.9200 - val_loss: 0.6336 - val_accuracy: 0.7
885 - 107s/epoch - 2s/step
Epoch 39/135
44/44 - 107s - loss: 0.2950 - accuracy: 0.8942 - val_loss: 0.4425 - val_accuracy: 0.8
696 - 107s/epoch - 2s/step
Epoch 40/135
44/44 - 108s - loss: 0.2761 - accuracy: 0.9125 - val_loss: 0.5996 - val_accuracy: 0.8
123 - 108s/epoch - 2s/step
```

```
Epoch 41/135
44/44 - 111s - loss: 0.2496 - accuracy: 0.9251 - val_loss: 0.4219 - val_accuracy: 0.8
617 - 111s/epoch - 3s/step
Epoch 42/135
44/44 - 108s - loss: 0.2307 - accuracy: 0.9184 - val_loss: 0.4835 - val_accuracy: 0.8
557 - 108s/epoch - 2s/step
Epoch 43/135
44/44 - 108s - loss: 0.2390 - accuracy: 0.9234 - val_loss: 0.5204 - val_accuracy: 0.8
103 - 108s/epoch - 2s/step
Epoch 44/135
44/44 - 108s - loss: 0.2521 - accuracy: 0.9150 - val_loss: 0.6555 - val_accuracy: 0.7
964 - 108s/epoch - 2s/step
Epoch 45/135
44/44 - 108s - loss: 0.1942 - accuracy: 0.9377 - val_loss: 0.4519 - val_accuracy: 0.8
538 - 108s/epoch - 2s/step
Epoch 46/135
44/44 - 107s - loss: 0.2081 - accuracy: 0.9268 - val_loss: 0.5088 - val_accuracy: 0.8
300 - 107s/epoch - 2s/step
Epoch 47/135
44/44 - 118s - loss: 0.2159 - accuracy: 0.9276 - val_loss: 0.7104 - val_accuracy: 0.7
905 - 118s/epoch - 3s/step
Epoch 48/135
44/44 - 119s - loss: 0.1960 - accuracy: 0.9276 - val_loss: 0.5685 - val_accuracy: 0.8
182 - 119s/epoch - 3s/step
Epoch 49/135
44/44 - 120s - loss: 0.1844 - accuracy: 0.9369 - val_loss: 0.4434 - val_accuracy: 0.8
478 - 120s/epoch - 3s/step
Epoch 50/135
44/44 - 120s - loss: 0.1892 - accuracy: 0.9419 - val_loss: 0.3575 - val_accuracy: 0.8
913 - 120s/epoch - 3s/step
Epoch 51/135
44/44 - 112s - loss: 0.1755 - accuracy: 0.9436 - val_loss: 0.4692 - val_accuracy: 0.8
577 - 112s/epoch - 3s/step
Epoch 52/135
44/44 - 109s - loss: 0.1546 - accuracy: 0.9478 - val_loss: 0.3678 - val_accuracy: 0.8
814 - 109s/epoch - 2s/step
Epoch 53/135
44/44 - 108s - loss: 0.2003 - accuracy: 0.9411 - val_loss: 0.4305 - val_accuracy: 0.8
735 - 108s/epoch - 2s/step
Epoch 54/135
44/44 - 108s - loss: 0.2079 - accuracy: 0.9335 - val_loss: 0.3648 - val_accuracy: 0.8
854 - 108s/epoch - 2s/step
Epoch 55/135
44/44 - 109s - loss: 0.2055 - accuracy: 0.9386 - val_loss: 0.4005 - val_accuracy: 0.8
696 - 109s/epoch - 2s/step
Epoch 56/135
44/44 - 108s - loss: 0.1652 - accuracy: 0.9450 - val_loss: 0.5542 - val_accuracy: 0.8
340 - 108s/epoch - 2s/step
Epoch 57/135
44/44 - 110s - loss: 0.1681 - accuracy: 0.9478 - val_loss: 0.4799 - val_accuracy: 0.8
399 - 110s/epoch - 2s/step
Epoch 58/135
44/44 - 109s - loss: 0.1565 - accuracy: 0.9478 - val_loss: 0.3892 - val_accuracy: 0.8
794 - 109s/epoch - 2s/step
Epoch 59/135
44/44 - 110s - loss: 0.1290 - accuracy: 0.9554 - val_loss: 0.4611 - val_accuracy: 0.8
755 - 110s/epoch - 3s/step
Epoch 60/135
44/44 - 109s - loss: 0.1326 - accuracy: 0.9554 - val_loss: 0.4140 - val_accuracy: 0.8
676 - 109s/epoch - 2s/step
```

```
Epoch 61/135
44/44 - 110s - loss: 0.1264 - accuracy: 0.9579 - val_loss: 0.4125 - val_accuracy: 0.8
735 - 110s/epoch - 3s/step
Epoch 62/135
44/44 - 110s - loss: 0.1506 - accuracy: 0.9545 - val_loss: 0.4685 - val_accuracy: 0.8
557 - 110s/epoch - 2s/step
Epoch 63/135
44/44 - 111s - loss: 0.1707 - accuracy: 0.9436 - val_loss: 0.5943 - val_accuracy: 0.8
162 - 111s/epoch - 3s/step
Epoch 64/135
44/44 - 109s - loss: 0.1632 - accuracy: 0.9335 - val_loss: 0.4667 - val_accuracy: 0.8
617 - 109s/epoch - 2s/step
Epoch 65/135
44/44 - 110s - loss: 0.1673 - accuracy: 0.9461 - val_loss: 0.3561 - val_accuracy: 0.8
933 - 110s/epoch - 3s/step
Epoch 66/135
44/44 - 111s - loss: 0.1153 - accuracy: 0.9621 - val_loss: 0.3567 - val_accuracy: 0.8
794 - 111s/epoch - 3s/step
Epoch 67/135
44/44 - 111s - loss: 0.1274 - accuracy: 0.9577 - val_loss: 0.4520 - val_accuracy: 0.8
814 - 111s/epoch - 3s/step
Epoch 68/135
44/44 - 110s - loss: 0.1167 - accuracy: 0.9638 - val_loss: 0.4170 - val_accuracy: 0.8
834 - 110s/epoch - 3s/step
Epoch 69/135
44/44 - 110s - loss: 0.1196 - accuracy: 0.9604 - val_loss: 0.4094 - val_accuracy: 0.8
775 - 110s/epoch - 2s/step
Epoch 70/135
44/44 - 111s - loss: 0.1403 - accuracy: 0.9478 - val_loss: 0.4735 - val_accuracy: 0.8
617 - 111s/epoch - 3s/step
Epoch 71/135
44/44 - 111s - loss: 0.1435 - accuracy: 0.9537 - val_loss: 0.5254 - val_accuracy: 0.8
577 - 111s/epoch - 3s/step
Epoch 72/135
44/44 - 111s - loss: 0.1371 - accuracy: 0.9638 - val_loss: 0.4595 - val_accuracy: 0.8
814 - 111s/epoch - 3s/step
Epoch 73/135
44/44 - 112s - loss: 0.1255 - accuracy: 0.9520 - val_loss: 0.3864 - val_accuracy: 0.8
834 - 112s/epoch - 3s/step
Epoch 74/135
44/44 - 118s - loss: 0.0817 - accuracy: 0.9773 - val_loss: 0.4611 - val_accuracy: 0.8
874 - 118s/epoch - 3s/step
Epoch 75/135
44/44 - 112s - loss: 0.1334 - accuracy: 0.9630 - val_loss: 0.5299 - val_accuracy: 0.8
498 - 112s/epoch - 3s/step
Epoch 76/135
44/44 - 114s - loss: 0.1318 - accuracy: 0.9562 - val_loss: 0.4291 - val_accuracy: 0.8
893 - 114s/epoch - 3s/step
Epoch 77/135
44/44 - 113s - loss: 0.1344 - accuracy: 0.9571 - val_loss: 0.5749 - val_accuracy: 0.8
518 - 113s/epoch - 3s/step
Epoch 78/135
44/44 - 112s - loss: 0.1194 - accuracy: 0.9638 - val_loss: 0.5399 - val_accuracy: 0.8
419 - 112s/epoch - 3s/step
Epoch 79/135
44/44 - 112s - loss: 0.1195 - accuracy: 0.9630 - val_loss: 0.5632 - val_accuracy: 0.8
202 - 112s/epoch - 3s/step
Epoch 80/135
44/44 - 111s - loss: 0.1024 - accuracy: 0.9680 - val_loss: 0.4331 - val_accuracy: 0.8
735 - 111s/epoch - 3s/step
```

```
Epoch 81/135
44/44 - 111s - loss: 0.1379 - accuracy: 0.9596 - val_loss: 0.5515 - val_accuracy: 0.8
518 - 111s/epoch - 3s/step
Epoch 82/135
44/44 - 112s - loss: 0.1223 - accuracy: 0.9554 - val_loss: 0.5360 - val_accuracy: 0.8
379 - 112s/epoch - 3s/step
Epoch 83/135
44/44 - 112s - loss: 0.1129 - accuracy: 0.9646 - val_loss: 0.4430 - val_accuracy: 0.8
538 - 112s/epoch - 3s/step
Epoch 84/135
44/44 - 112s - loss: 0.0871 - accuracy: 0.9646 - val_loss: 0.6311 - val_accuracy: 0.8
360 - 112s/epoch - 3s/step
Epoch 85/135
44/44 - 111s - loss: 0.1216 - accuracy: 0.9571 - val_loss: 0.5109 - val_accuracy: 0.8
656 - 111s/epoch - 3s/step
Epoch 86/135
44/44 - 112s - loss: 0.1119 - accuracy: 0.9663 - val_loss: 0.5087 - val_accuracy: 0.8
617 - 112s/epoch - 3s/step
Epoch 87/135
44/44 - 112s - loss: 0.1379 - accuracy: 0.9579 - val_loss: 0.4302 - val_accuracy: 0.8
557 - 112s/epoch - 3s/step
Epoch 88/135
44/44 - 111s - loss: 0.1264 - accuracy: 0.9596 - val_loss: 0.4859 - val_accuracy: 0.8
518 - 111s/epoch - 3s/step
Epoch 89/135
44/44 - 112s - loss: 0.1073 - accuracy: 0.9655 - val_loss: 0.4251 - val_accuracy: 0.8
854 - 112s/epoch - 3s/step
Epoch 90/135
44/44 - 111s - loss: 0.0957 - accuracy: 0.9663 - val_loss: 0.4354 - val_accuracy: 0.8
834 - 111s/epoch - 3s/step
Epoch 91/135
44/44 - 112s - loss: 0.1025 - accuracy: 0.9680 - val_loss: 0.4127 - val_accuracy: 0.8
874 - 112s/epoch - 3s/step
Epoch 92/135
44/44 - 112s - loss: 0.1209 - accuracy: 0.9562 - val_loss: 0.2961 - val_accuracy: 0.9
012 - 112s/epoch - 3s/step
Epoch 93/135
44/44 - 112s - loss: 0.1179 - accuracy: 0.9628 - val_loss: 0.4529 - val_accuracy: 0.8
715 - 112s/epoch - 3s/step
Epoch 94/135
44/44 - 111s - loss: 0.1026 - accuracy: 0.9613 - val_loss: 0.3650 - val_accuracy: 0.9
012 - 111s/epoch - 3s/step
Epoch 95/135
44/44 - 112s - loss: 0.1019 - accuracy: 0.9689 - val_loss: 0.5867 - val_accuracy: 0.8
538 - 112s/epoch - 3s/step
Epoch 96/135
44/44 - 111s - loss: 0.1050 - accuracy: 0.9630 - val_loss: 0.4031 - val_accuracy: 0.8
834 - 111s/epoch - 3s/step
Epoch 97/135
44/44 - 111s - loss: 0.0873 - accuracy: 0.9672 - val_loss: 0.4630 - val_accuracy: 0.8
755 - 111s/epoch - 3s/step
Epoch 98/135
44/44 - 112s - loss: 0.1079 - accuracy: 0.9630 - val_loss: 0.5294 - val_accuracy: 0.8
538 - 112s/epoch - 3s/step
Epoch 99/135
44/44 - 111s - loss: 0.0909 - accuracy: 0.9672 - val_loss: 0.3111 - val_accuracy: 0.9
071 - 111s/epoch - 3s/step
Epoch 100/135
44/44 - 112s - loss: 0.0728 - accuracy: 0.9714 - val_loss: 0.2941 - val_accuracy: 0.9
150 - 112s/epoch - 3s/step
```

```
Epoch 101/135
44/44 - 112s - loss: 0.1050 - accuracy: 0.9638 - val_loss: 0.2619 - val_accuracy: 0.9
170 - 112s/epoch - 3s/step
Epoch 102/135
44/44 - 112s - loss: 0.0692 - accuracy: 0.9747 - val_loss: 0.4539 - val_accuracy: 0.8
775 - 112s/epoch - 3s/step
Epoch 103/135
44/44 - 111s - loss: 0.0854 - accuracy: 0.9680 - val_loss: 0.3114 - val_accuracy: 0.9
091 - 111s/epoch - 3s/step
Epoch 104/135
44/44 - 112s - loss: 0.1130 - accuracy: 0.9672 - val_loss: 0.3574 - val_accuracy: 0.8
676 - 112s/epoch - 3s/step
Epoch 105/135
44/44 - 111s - loss: 0.0872 - accuracy: 0.9722 - val_loss: 0.3758 - val_accuracy: 0.8
992 - 111s/epoch - 3s/step
Epoch 106/135
44/44 - 116s - loss: 0.1195 - accuracy: 0.9638 - val_loss: 0.3113 - val_accuracy: 0.9
170 - 116s/epoch - 3s/step
Epoch 107/135
44/44 - 113s - loss: 0.0794 - accuracy: 0.9739 - val_loss: 0.3893 - val_accuracy: 0.8
913 - 113s/epoch - 3s/step
Epoch 108/135
44/44 - 112s - loss: 0.1129 - accuracy: 0.9596 - val_loss: 0.5391 - val_accuracy: 0.8
498 - 112s/epoch - 3s/step
Epoch 109/135
44/44 - 112s - loss: 0.0812 - accuracy: 0.9714 - val_loss: 0.3582 - val_accuracy: 0.9
071 - 112s/epoch - 3s/step
Epoch 110/135
44/44 - 112s - loss: 0.0669 - accuracy: 0.9756 - val_loss: 0.4542 - val_accuracy: 0.8
676 - 112s/epoch - 3s/step
Epoch 111/135
44/44 - 113s - loss: 0.0676 - accuracy: 0.9798 - val_loss: 0.3915 - val_accuracy: 0.8
953 - 113s/epoch - 3s/step
Epoch 112/135
44/44 - 112s - loss: 0.0642 - accuracy: 0.9790 - val_loss: 0.4212 - val_accuracy: 0.8
933 - 112s/epoch - 3s/step
Epoch 113/135
44/44 - 113s - loss: 0.0754 - accuracy: 0.9764 - val_loss: 0.4139 - val_accuracy: 0.8
893 - 113s/epoch - 3s/step
Epoch 114/135
44/44 - 113s - loss: 0.0658 - accuracy: 0.9815 - val_loss: 0.6727 - val_accuracy: 0.8
162 - 113s/epoch - 3s/step
Epoch 115/135
44/44 - 112s - loss: 0.0819 - accuracy: 0.9773 - val_loss: 0.4391 - val_accuracy: 0.8
972 - 112s/epoch - 3s/step
Epoch 116/135
44/44 - 113s - loss: 0.0788 - accuracy: 0.9731 - val_loss: 0.4211 - val_accuracy: 0.8
794 - 113s/epoch - 3s/step
Epoch 117/135
44/44 - 112s - loss: 0.0697 - accuracy: 0.9773 - val_loss: 0.4373 - val_accuracy: 0.8
972 - 112s/epoch - 3s/step
Epoch 118/135
44/44 - 113s - loss: 0.0687 - accuracy: 0.9731 - val_loss: 0.2872 - val_accuracy: 0.9
229 - 113s/epoch - 3s/step
Epoch 119/135
44/44 - 113s - loss: 0.0631 - accuracy: 0.9798 - val_loss: 0.6382 - val_accuracy: 0.8
439 - 113s/epoch - 3s/step
Epoch 120/135
44/44 - 113s - loss: 0.0755 - accuracy: 0.9731 - val_loss: 0.4946 - val_accuracy: 0.8
676 - 113s/epoch - 3s/step
```

```
Epoch 121/135
44/44 - 112s - loss: 0.0930 - accuracy: 0.9672 - val_loss: 0.4392 - val_accuracy: 0.8
893 - 112s/epoch - 3s/step
Epoch 122/135
44/44 - 113s - loss: 0.1065 - accuracy: 0.9689 - val_loss: 0.3376 - val_accuracy: 0.9
012 - 113s/epoch - 3s/step
Epoch 123/135
44/44 - 114s - loss: 0.0775 - accuracy: 0.9790 - val_loss: 0.5674 - val_accuracy: 0.8
676 - 114s/epoch - 3s/step
Epoch 124/135
44/44 - 112s - loss: 0.0916 - accuracy: 0.9689 - val_loss: 0.4543 - val_accuracy: 0.8
893 - 112s/epoch - 3s/step
Epoch 125/135
44/44 - 114s - loss: 0.0845 - accuracy: 0.9756 - val_loss: 0.3850 - val_accuracy: 0.8
874 - 114s/epoch - 3s/step
Epoch 126/135
44/44 - 114s - loss: 0.0686 - accuracy: 0.9781 - val_loss: 0.4696 - val_accuracy: 0.8
854 - 114s/epoch - 3s/step
Epoch 127/135
44/44 - 112s - loss: 0.0860 - accuracy: 0.9714 - val_loss: 0.3663 - val_accuracy: 0.8
992 - 112s/epoch - 3s/step
Epoch 128/135
44/44 - 113s - loss: 0.0713 - accuracy: 0.9798 - val_loss: 0.4435 - val_accuracy: 0.8
893 - 113s/epoch - 3s/step
Epoch 129/135
44/44 - 113s - loss: 0.0999 - accuracy: 0.9663 - val_loss: 0.3398 - val_accuracy: 0.9
150 - 113s/epoch - 3s/step
Epoch 130/135
44/44 - 112s - loss: 0.0724 - accuracy: 0.9705 - val_loss: 0.5148 - val_accuracy: 0.8
735 - 112s/epoch - 3s/step
Epoch 131/135
44/44 - 114s - loss: 0.0721 - accuracy: 0.9756 - val_loss: 0.4415 - val_accuracy: 0.8
814 - 114s/epoch - 3s/step
Epoch 132/135
44/44 - 112s - loss: 0.0825 - accuracy: 0.9722 - val_loss: 0.5198 - val_accuracy: 0.8
676 - 112s/epoch - 3s/step
Epoch 133/135
44/44 - 112s - loss: 0.1018 - accuracy: 0.9697 - val_loss: 0.3470 - val_accuracy: 0.9
051 - 112s/epoch - 3s/step
Epoch 134/135
44/44 - 113s - loss: 0.1025 - accuracy: 0.9663 - val_loss: 0.5546 - val_accuracy: 0.8
419 - 113s/epoch - 3s/step
Epoch 135/135
44/44 - 113s - loss: 0.0632 - accuracy: 0.9806 - val_loss: 0.2680 - val_accuracy: 0.9
289 - 113s/epoch - 3s/step
Time:  4:09:07.818492
```

In [33]:

```
score = model.evaluate(train_generator, verbose=0)
print("Accuracy: %.2f%%" % (score[1]*100))
```

```
Accuracy: 98.47%
```

In [34]:

```
model.metrics_names
```

Out[34]:

```
['loss', 'accuracy']
```

In [35]:

```
score
```

Out[35]:

```
[0.04696470499038696, 0.9847216010093689]
```

In [36]:
```
loss = history.history['loss']
val_loss = history.history['val_loss']
epochs = range(1, len(loss) + 1)
plt.plot(epochs, loss, 'g', label='Training loss')
plt.plot(epochs, val_loss, 'b', label='Validation loss')
plt.title('Training and validation loss')
plt.xlabel('Epochs')
plt.ylabel('Loss')
plt.legend()
plt.show()

acc =history.history['accuracy']
val_acc = history.history['val_accuracy']
plt.plot(epochs, acc, 'r', label='Training acc')
plt.plot(epochs, val_acc, 'y', label='Validation acc')
plt.title('Training and validation accuracy')
plt.xlabel('Epochs')
plt.ylabel('Accuracy')
plt.legend()
plt.show()
```

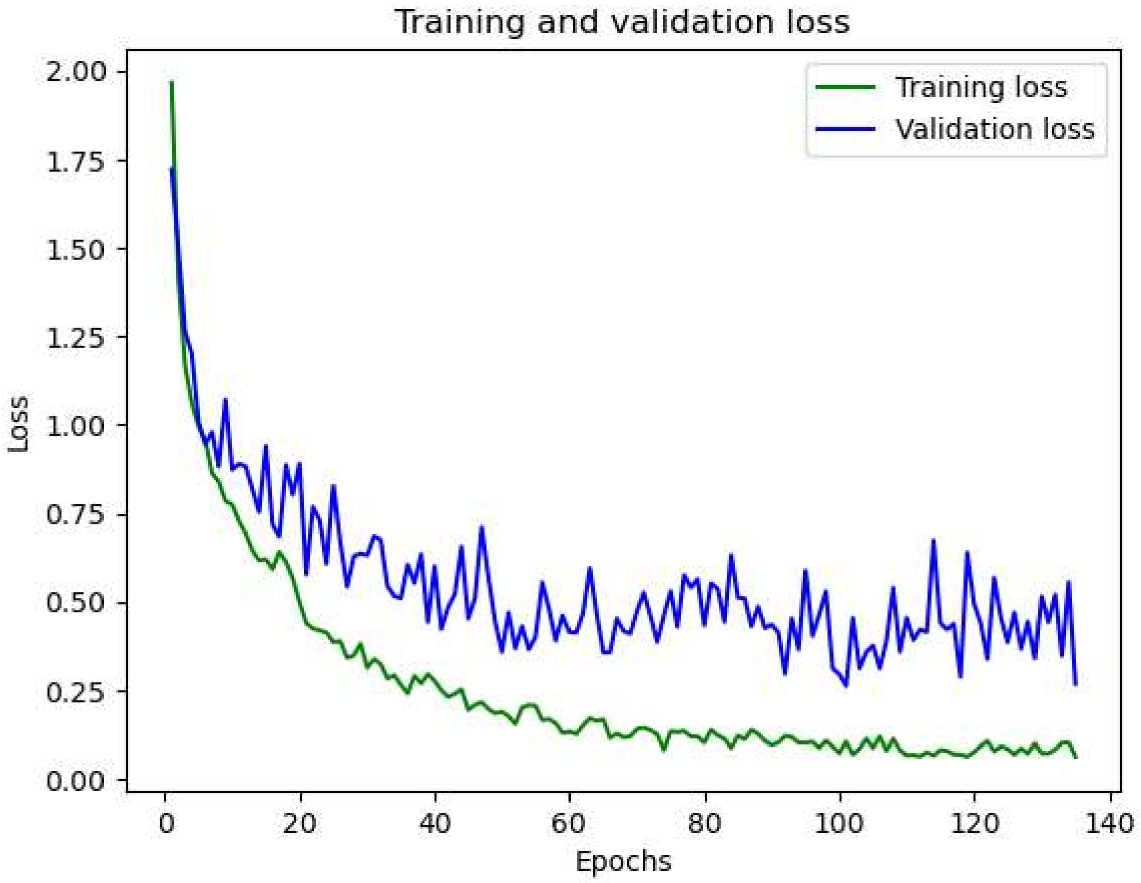

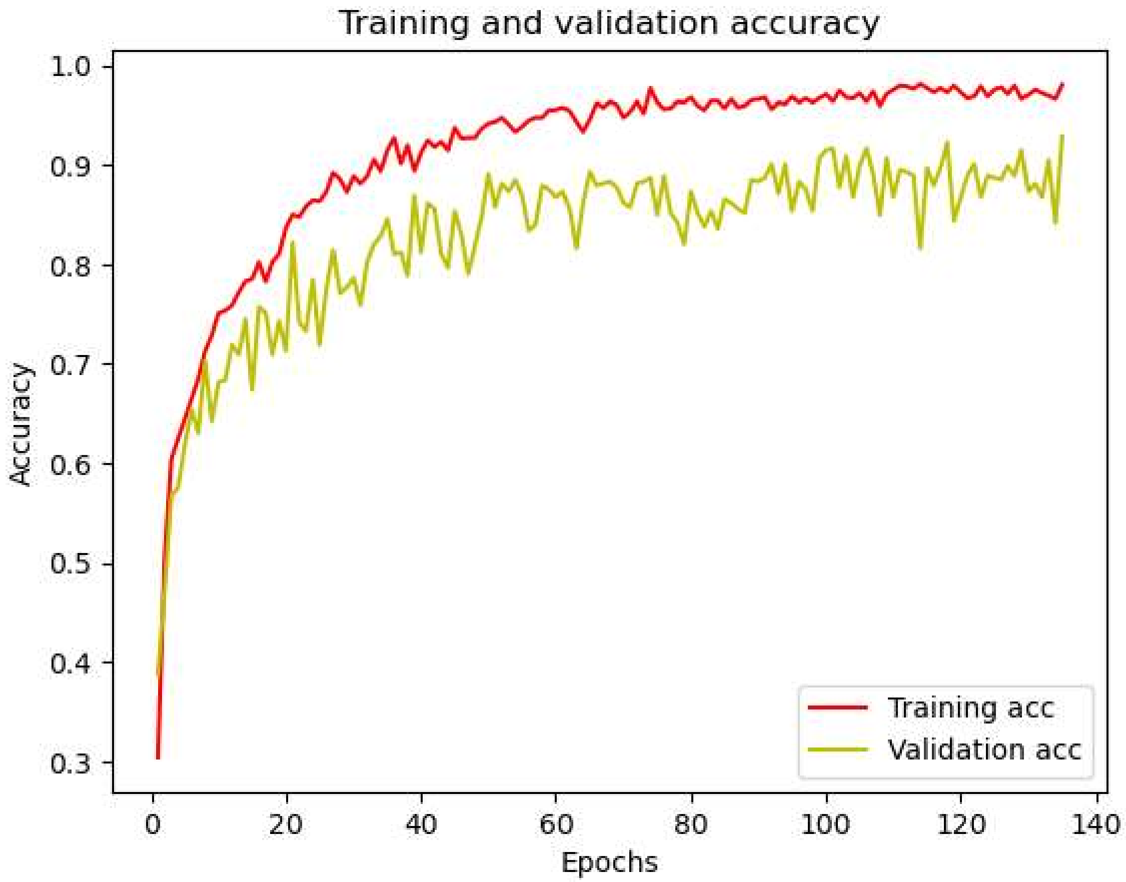

In [37]:
```
class_labels =["AD", "AL", "BL", "FR", "LA", "PM", "RE", "SA", "YE" ]
from sklearn.metrics import classification_report
test_labels = test_batches.classes
predictions = model.predict(test_batches, steps=len(test_batches), verbose=0)
y_pred = np.argmax (predictions, axis= -1)
print(classification_report(test_labels, y_pred, target_names=class_labels))
```

```
              precision    recall  f1-score   support

          AD       1.00      0.97      0.99        40
          AL       0.87      0.82      0.85        40
          BL       0.98      1.00      0.99        40
          FR       0.95      0.93      0.94        40
          LA       0.97      0.85      0.91        40
          PM       0.97      0.93      0.95        40
          RE       0.95      0.93      0.94        40
          SA       0.89      1.00      0.94        40
          YE       0.85      0.97      0.91        40

    accuracy                           0.93       360
   macro avg       0.94      0.93      0.93       360
weighted avg       0.94      0.93      0.93       360
```

In [38]:
```
test_labels = test_batches.classes
```

In [39]:
```
predictions = model.predict(x=test_batches, steps=len(test_batches), verbose=0)
```

In [40]:
```
cm = confusion_matrix(y_true=test_batches.classes, y_pred= np.argmax(predictions,axis=
```

In [41]:

```
#Plot the confusion matrix. Set Normalize = True/False



def plot_confusion_matrix(cm, classes, normalize=True, title='Confusion matrix', cmap=

    """

    This function prints and plots the confusion matrix.

    Normalization can be applied by setting `normalize=True`.

    """

    plt.figure(figsize=(8,6))


    plt.imshow(cm, interpolation='nearest', cmap=cmap)

    plt.title(title)

    plt.colorbar()


    tick_marks = np.arange(len(classes))

    plt.xticks(tick_marks, classes, rotation=45)

    plt.yticks(tick_marks, classes)



    if normalize:

        cm = cm.astype('float') / cm.sum(axis=1)[:, np.newaxis]
        cm = np.around(cm, decimals=2)
        cm[np.isnan(cm)] = 0.0

        print("Normalized confusion matrix")

    else:

        print('Confusion matrix, without normalization')

    thresh = cm.max() / 2.

    for i, j in itertools.product(range(cm.shape[0]), range(cm.shape[1])):

        plt.text(j, i, cm[i, j],

                 horizontalalignment="center",
                 fontsize="15",

                 color="white" if cm[i, j] > thresh else "black")

    plt.tight_layout()

    plt.ylabel('True label')
```

```
    plt.xlabel('Predicted label')
```

In [42]:
```
cm_plot_labels = ["AD", "AL", "BL", "FR", "LA", "PM", "RE", "SA", "YE"]
plot_confusion_matrix(cm=cm, classes=cm_plot_labels, title='Confusion Matrix')
```

Normalized confusion matrix

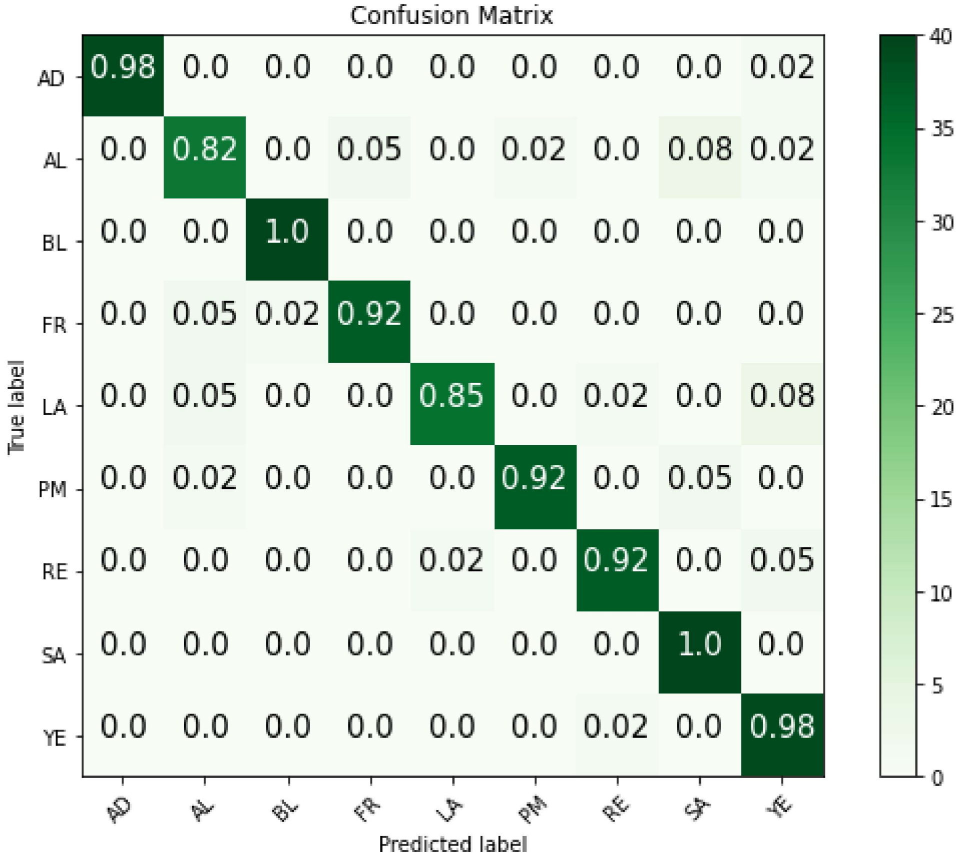

In [43]:
```
testX, testy = test_batches.next()
print('test shape=%s, min=%.3f, max=%.3f' % (testX.shape, testX.min(), testX.max()))
```

```
test shape=(360, 224, 224, 3), min=-1.000, max=1.000
```

In [44]:
```
#making a prediction about our test data

#checking the prediction shape
predictions.shape
```

Out[44]:
```
(360, 9)
```

In [45]:
```
#checking the batch shape
testX.shape
```

Out[45]:
```
(360, 224, 224, 3)
```

In [46]:
```
predictions= model.predict(testX)
```

```
12/12 [==============================] - 8s 644ms/step
```

In [47]:
```
test_loss, test_accuracy = model.evaluate(testX, testy)
```

```
12/12 [==============================] - 8s 689ms/step - loss: 0.7201 - accuracy: 0.8
389
```

In [48]:
```
predictions[0]
```

Out[48]:
```
array([9.9995685e-01, 4.0995194e-05, 3.4780383e-11, 1.6994771e-10,
       4.2968284e-07, 2.1204228e-07, 1.3828451e-06, 2.1488589e-08,
       3.5806341e-08], dtype=float32)
```

In [49]:
```
plt.figure()
plt.imshow(testX[0])
plt.colorbar()
plt.show()
```

```
Clipping input data to the valid range for imshow with RGB data ([0..1] for floats or
[0..255] for integers).
```

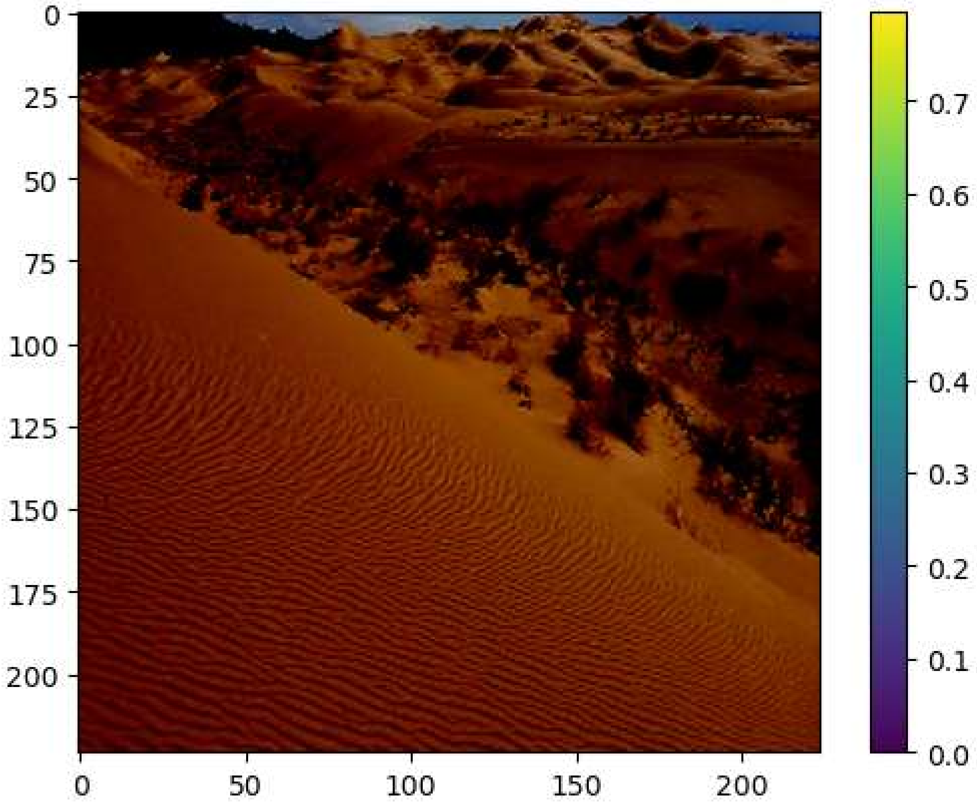

In [50]:
```
# function to plot an image
def plot_image(i, predictions_array, true_label, img):
 predictions_array, true_label, img=predictions_array, true_label[i], img[i]
 plt.grid(False)
 plt.xticks([])
 plt.yticks([])

 plt.imshow(img, cmap=plt.cm.gray)

 predicted_label = np.argmax(predictions_array)
 if predicted_label==true_label:
    color='blue'
    plt.xlabel("{} {:2.0f}% ({})".format(class_names[predicted_label],
                                         100*np.max(predictions_array),
                                         class_names[true_label]),
                                         color=color)
 else:
```

```
    color='red'
    plt.xlabel("{} {:2.0f}% ({})".format(class_names[predicted_label],
                                         100*np.max(predictions_array),
                                         class_names[true_label]),
                                         color=color)

#functions to create bar plot of the predictions
def plot_value_array(i, predictions_array, true_label):
 predictions_array, true_label = predictions_array, true_label[i]
 plt.grid(False)
 plt.xticks(range(9))
 plt.yticks([])
 thisplot = plt.bar(range(9), predictions_array, color="#777777")
 plt.ylim([0, 1])
 predicted_label=np.argmax(predictions_array)

 thisplot[predicted_label].set_color('red')
 thisplot[true_label].set_color('blue')
```

In [51]:

```
class_names = ["Arid", "Alluvial", "Black", "Forest", "Laterite", "Peaty/Marshy", "Re
```

In [52]:

```
for i in range(9):
    plt.figure(figsize=(6,3))
    plt.subplot(1,2,1)
    plot_image(i, predictions[i], test_labels, testX)
    plt.subplot(1,2,2)
    plot_value_array(i, predictions[i], test_labels)
    plt.show()
```

```
Clipping input data to the valid range for imshow with RGB data ([0..1] for floats or
[0..255] for integers).
```

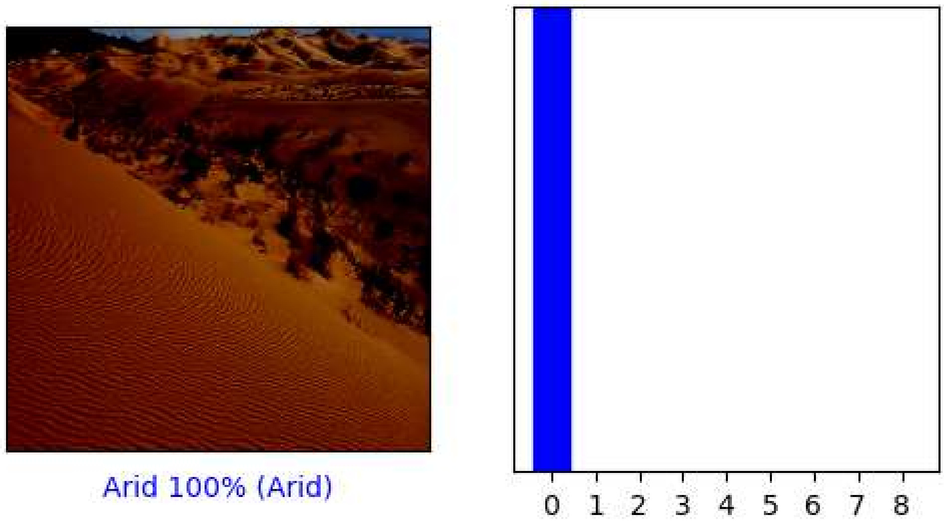


```
Clipping input data to the valid range for imshow with RGB data ([0..1] for floats or
[0..255] for integers).
```

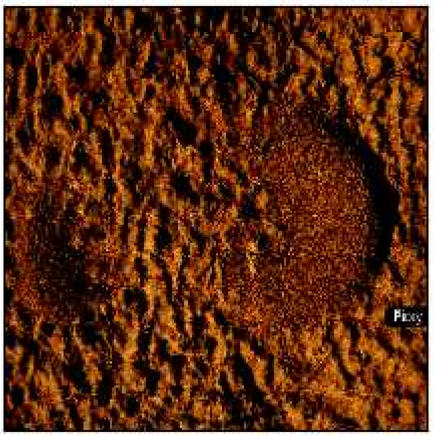

Arid 93% (Arid)

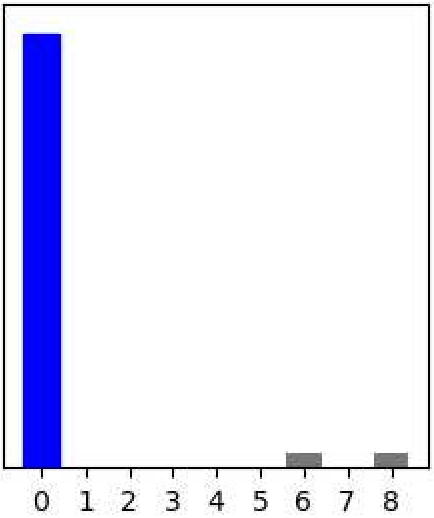


```
Clipping input data to the valid range for imshow with RGB data ([0..1] for floats or
[0..255] for integers).
```

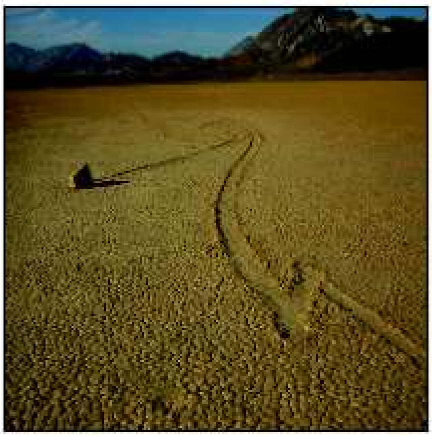

Arid 100% (Arid)

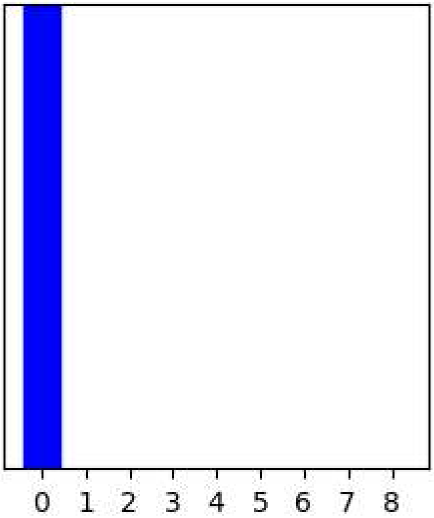


```
Clipping input data to the valid range for imshow with RGB data ([0..1] for floats or
[0..255] for integers).
```

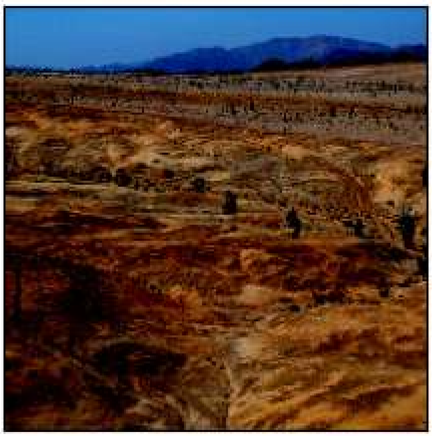

Arid 100% (Arid)

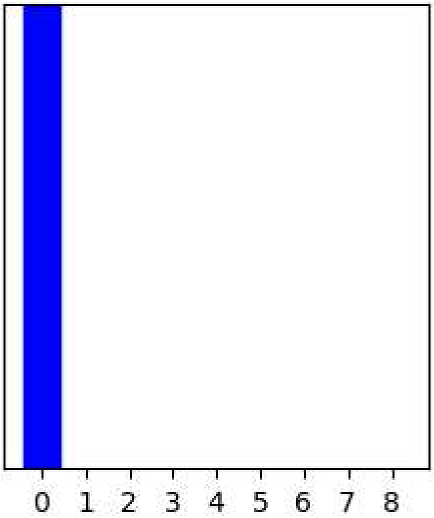


```
Clipping input data to the valid range for imshow with RGB data ([0..1] for floats or
[0..255] for integers).
```

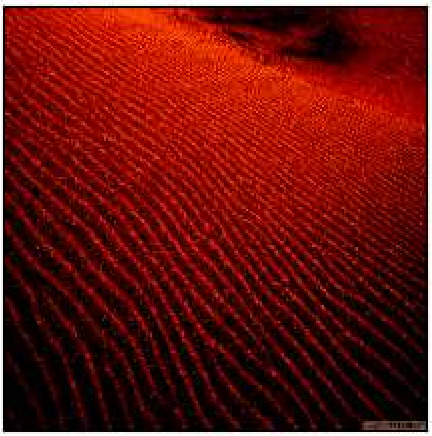

Arid 100% (Arid)

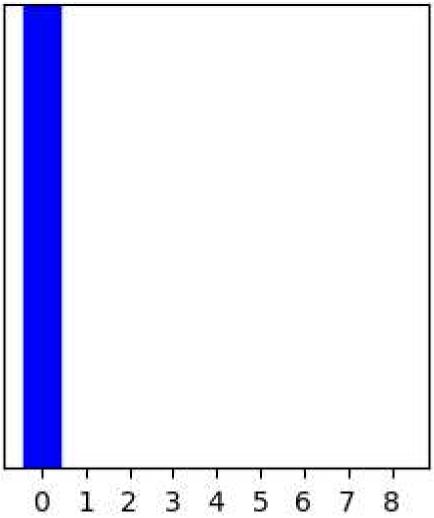


```
Clipping input data to the valid range for imshow with RGB data ([0..1] for floats or
[0..255] for integers).
```

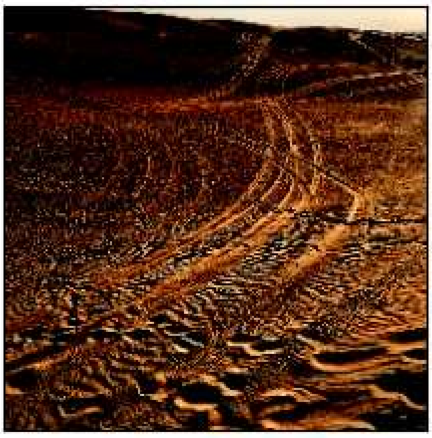

Arid 100% (Arid)

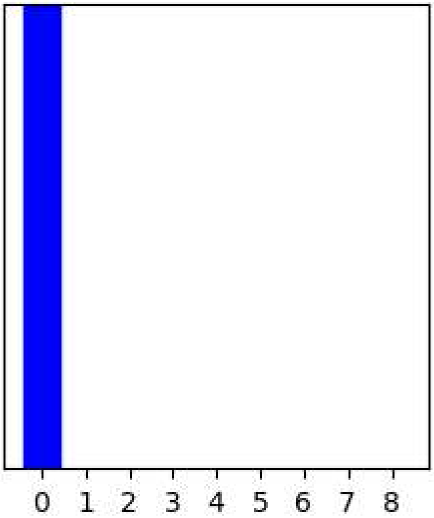


```
Clipping input data to the valid range for imshow with RGB data ([0..1] for floats or
[0..255] for integers).
```

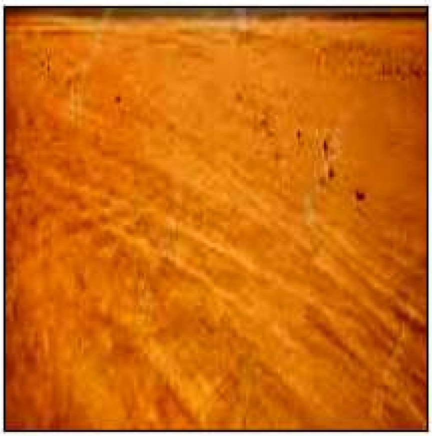

Arid 100% (Arid)

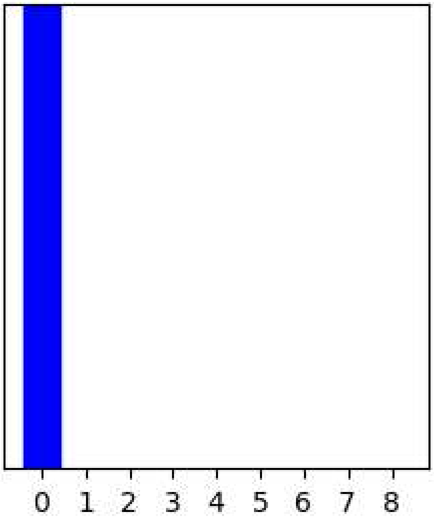


```
Clipping input data to the valid range for imshow with RGB data ([0..1] for floats or
[0..255] for integers).
```

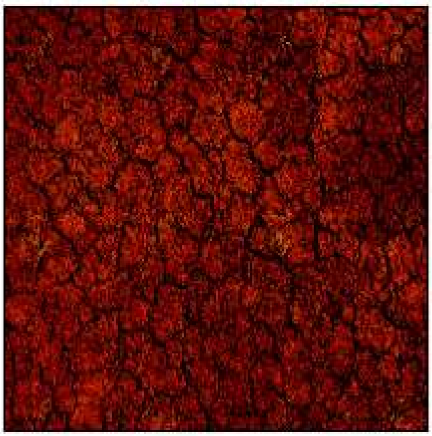

Arid 100% (Arid)

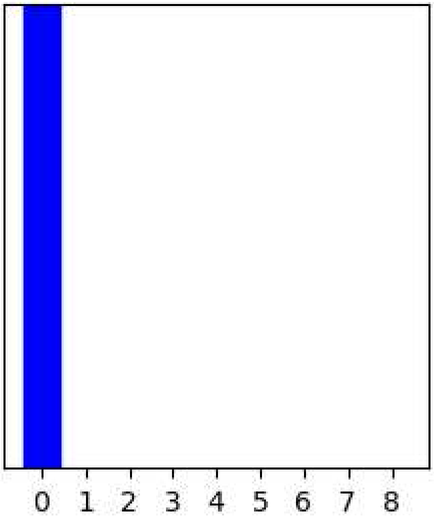


```
Clipping input data to the valid range for imshow with RGB data ([0..1] for floats or
[0..255] for integers).
```

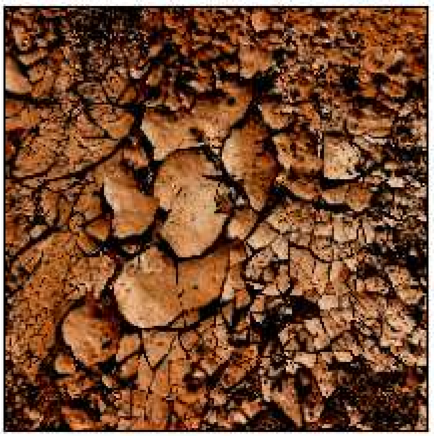

Arid 100% (Arid)

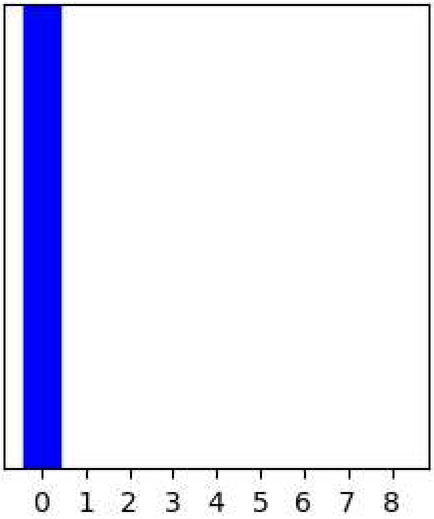

In [53]:
```
i=40
plt.figure(figsize=(6,3))
plt.subplot(1,2,1)
plot_image(i, predictions[i], test_labels, testX)
plt.subplot(1,2,2)
plot_value_array(i, predictions[i], test_labels)
plt.show()
```

```
Clipping input data to the valid range for imshow with RGB data ([0..1] for floats or
[0..255] for integers).
```

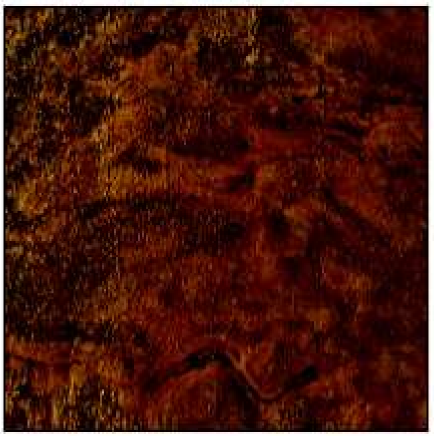

Alluvial 93% (Alluvial)

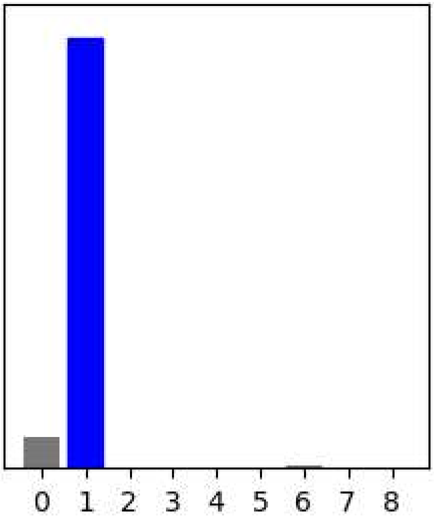

In [54]:
```
i=60
plt.figure(figsize=(6,3))
plt.subplot(1,2,1)
plot_image(i, predictions[i], test_labels, testX)
plt.subplot(1,2,2)
plot_value_array(i, predictions[i], test_labels)
plt.show()
```

```
Clipping input data to the valid range for imshow with RGB data ([0..1] for floats or
[0..255] for integers).
```

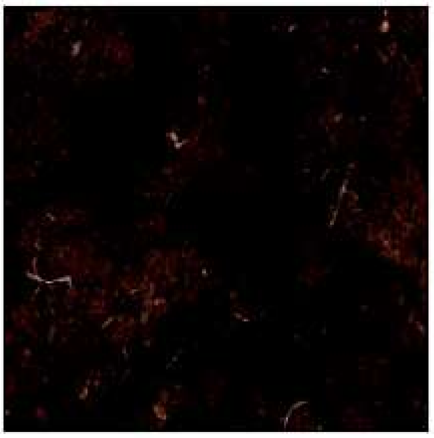

Alluvial 99% (Alluvial)

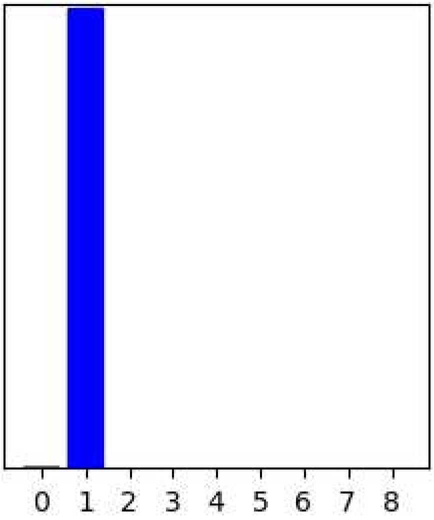

In [55]:
```
predicted_classes = model.predict(x=test_batches, steps=len(test_batches), verbose=0)
predicted_classes = np.argmax(np.round(predicted_classes),axis=1)
predicted_classes.shape, y_pred.shape
#print(type(predictions), predictions.shape)
```

Out[55]:
```
((360,), (360,))
```

In [56]:
```
plt.figure(figsize=(10, 10))
correct = np.where(predicted_classes==y_pred)[0]
print (("Found %d correct labels") % len(correct))
for i, correct in enumerate(correct[:9]):
    plt.subplot(3,3,i+1)
    plt.imshow(testX[correct], cmap='gray', interpolation='none')
```

```
    plt.title("Predicted {}, Class {}".format(predicted_classes[correct], y_pred[corre
    plt.tight_layout()
```

```
Clipping input data to the valid range for imshow with RGB data ([0..1] for floats or
[0..255] for integers).
Clipping input data to the valid range for imshow with RGB data ([0..1] for floats or
[0..255] for integers).
Clipping input data to the valid range for imshow with RGB data ([0..1] for floats or
[0..255] for integers).
Found 356 correct labels
Clipping input data to the valid range for imshow with RGB data ([0..1] for floats or
[0..255] for integers).
Clipping input data to the valid range for imshow with RGB data ([0..1] for floats or
[0..255] for integers).
Clipping input data to the valid range for imshow with RGB data ([0..1] for floats or
[0..255] for integers).
Clipping input data to the valid range for imshow with RGB data ([0..1] for floats or
[0..255] for integers).
Clipping input data to the valid range for imshow with RGB data ([0..1] for floats or
[0..255] for integers).
Clipping input data to the valid range for imshow with RGB data ([0..1] for floats or
[0..255] for integers).
```

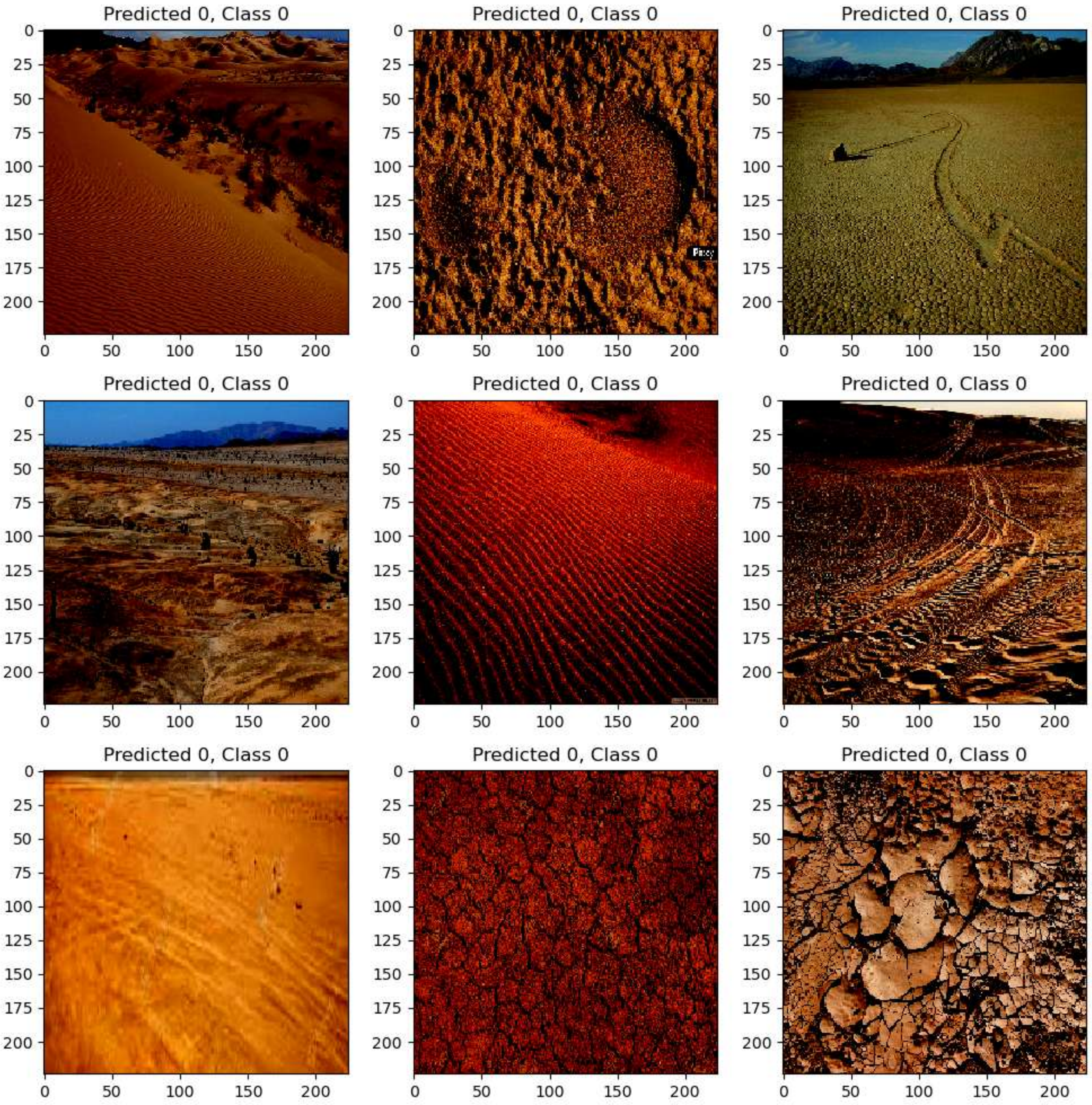

In [57]:

```
#printing the first element from predicted data
pred=model.predict(test_batches)
print(pred[0])
#printing the index of
print('Index:',np.argmax(pred[0]))
```

```
1/1 [==============================] - 8s 8s/step
[9.9995685e-01 4.0995194e-05 3.4780383e-11 1.6994706e-10 4.2968244e-07
 2.1204208e-07 1.3828437e-06 2.1488546e-08 3.5806274e-08]
Index: 0
```

In [58]:

```
predictions = model.predict(test_batches)
for i in range(len(test_batches)):
 print("X=%s, Predicted=%s" % (test_batches[i], predictions[i]))
```

```
1/1 [==============================] - 9s 9s/step
X=(array([[[[-0.5764706 , -0.5372549 , -0.38039213],
         [-0.5294118 , -0.4823529 , -0.32549018],
         [-0.45098037, -0.3960784 , -0.25490195],
         ...,
         [ 0.19215691,  0.34901965,  0.56078434],
         [ 0.19215691,  0.34901965,  0.56078434],
         [ 0.19215691,  0.34901965,  0.56078434]],

        [[-0.62352943, -0.58431375, -0.42745095],
         [-0.6156863 , -0.56078434, -0.41176468],
         [-0.6627451 , -0.60784316, -0.46666664],
         ...,
         [ 0.18431377,  0.3411765 ,  0.5529412 ],
         [ 0.19215691,  0.34901965,  0.56078434],
         [ 0.19215691,  0.34901965,  0.56078434]],

        [[-0.62352943, -0.58431375, -0.4352941 ],
         [-0.654902  , -0.60784316, -0.4588235 ],
         [-0.7019608 , -0.64705884, -0.5058824 ],
         ...,
         [ 0.18431377,  0.3411765 ,  0.5529412 ],
         [ 0.18431377,  0.3411765 ,  0.5529412 ],
         [ 0.19215691,  0.34901965,  0.56078434]],

        ...,

        [[ 0.27843142, -0.05882353, -0.3333333 ],
         [ 0.24705887, -0.09019607, -0.36470586],
         [ 0.21568632, -0.12156862, -0.3960784 ],
         ...,
         [ 0.4666667 ,  0.16078436, -0.08235294],
         [ 0.3803922 ,  0.07450986, -0.16862744],
         [ 0.4039216 ,  0.09019613, -0.14509803]],

        [[ 0.38823533,  0.05098045, -0.2235294 ],
         [ 0.38823533,  0.05098045, -0.2235294 ],
         [ 0.3411765 ,  0.01176476, -0.26274508],
         ...,
         [ 0.28627455, -0.01960784, -0.26274508],
         [ 0.4431373 ,  0.13725495, -0.10588235],
         [ 0.41960788,  0.11372554, -0.12941176]],

        [[ 0.33333337,  0.00392163, -0.27058822],
         [ 0.36470592,  0.02745104, -0.24705881],
         [ 0.37254906,  0.03529418, -0.23921567],
         ...,
         [ 0.427451  ,  0.12156868, -0.12156862],
         [ 0.3803922 ,  0.07450986, -0.16862744],
         [ 0.34901965,  0.04313731, -0.19999999]]],


       [[[ 0.24705887, -0.08235294, -0.38039213],
         [ 0.38823533,  0.05882359, -0.23921567],
         [ 0.35686278,  0.05098045, -0.35686272],
         ...,
         [ 0.48235297,  0.13725495, -0.23137254],
         [ 0.5921569 ,  0.24705887, -0.10588235],
         [ 0.37254906,  0.02745104, -0.32549018]],
```

```
 [[ 0.45098042,  0.12941182, -0.20784312],
  [ 0.26274514, -0.05882353, -0.3960784 ],
  [ 0.6627451 ,  0.3411765 , -0.01960784],
  ...,
  [ 0.47450984,  0.12941182, -0.23921567],
  [ 0.6       ,  0.254902  , -0.09803921],
  [ 0.37254906,  0.02745104, -0.32549018]],

 [[ 0.21568632, -0.09803921, -0.47450978],
  [ 0.2941177 , -0.01960784, -0.40392154],
  [ 0.54509807,  0.21568632, -0.11372548],
  ...,
  [ 0.4666667 ,  0.12156868, -0.24705881],
  [ 0.41960788,  0.07450986, -0.27843136],
  [ 0.56078434,  0.21568632, -0.1372549 ]],

 ...,

 [[ 0.26274514, -0.05882353, -0.38823527],
  [ 0.26274514, -0.06666666, -0.35686272],
  [ 0.28627455,  0.02745104, -0.38823527],
  ...,
  [ 0.4666667 ,  0.12156868, -0.26274508],
  [ 0.654902  ,  0.33333337, -0.02745098],
  [ 0.67058825,  0.34901965, -0.01176471]],

 [[-0.34117645, -0.60784316, -0.827451  ],
  [-0.27058822, -0.5529412 , -0.7254902 ],
  [-0.26274508, -0.4823529 , -0.70980394],
  ...,
  [ 0.69411767,  0.34901965, -0.03529412],
  [ 0.4901961 ,  0.1686275 , -0.19215685],
  [ 0.4666667 ,  0.14509809, -0.21568626]],

 [[-0.3098039 , -0.5137255 , -0.75686276],
  [-0.3490196 , -0.5529412 , -0.75686276],
  [-0.44313723, -0.60784316, -0.77254903],
  ...,
  [ 0.6313726 ,  0.28627455, -0.09803921],
  [ 0.5921569 ,  0.27058828, -0.09019607],
  [ 0.5372549 ,  0.21568632, -0.14509803]]],


[[[ 0.11372554,  0.37254906,  0.5058824 ],
  [ 0.12156868,  0.3803922 ,  0.5294118 ],
  [ 0.14509809,  0.38823533,  0.5529412 ],
  ...,
  [ 0.04313731,  0.38823533,  0.5921569 ],
  [ 0.06666672,  0.35686278,  0.5686275 ],
  [ 0.06666672,  0.36470592,  0.5764706 ]],

 [[ 0.12941182,  0.3803922 ,  0.52156866],
  [ 0.15294123,  0.38823533,  0.5294118 ],
  [ 0.1686275 ,  0.3803922 ,  0.5294118 ],
  ...,
  [ 0.05882359,  0.4039216 ,  0.6       ],
  [ 0.07450986,  0.38823533,  0.5921569 ],
  [ 0.06666672,  0.3803922 ,  0.58431375]],

 [[ 0.16078436,  0.3803922 ,  0.52156866],
```

```
   [ 0.18431377,  0.3803922 ,  0.5137255 ],
   [ 0.21568632,  0.3803922 ,  0.52156866],
   ...,
   [ 0.082353  ,  0.4039216 ,  0.6       ],
   [ 0.07450986,  0.38823533,  0.58431375],
   [ 0.082353  ,  0.39607847,  0.5921569 ]],

  ...,

  [[ 0.41960788,  0.3803922 ,  0.06666672],
   [ 0.04313731, -0.00392157, -0.29411763],
   [ 0.10588241,  0.082353  , -0.17647058],
   ...,
   [-0.01176471, -0.04313725, -0.16862744],
   [ 0.07450986,  0.00392163, -0.2235294 ],
   [ 0.30980396,  0.21568632, -0.09019607]],

  [[ 0.4039216 ,  0.36470592,  0.06666672],
   [-0.1372549 , -0.18431371, -0.45098037],
   [ 0.23921573,  0.20784318, -0.01176471],
   ...,
   [ 0.06666672, -0.01960784, -0.21568626],
   [ 0.16078436,  0.082353  , -0.19999999],
   [ 0.32549024,  0.22352946, -0.11372548]],

  [[ 0.24705887,  0.17647064, -0.1607843 ],
   [ 0.2313726 ,  0.17647064, -0.04313725],
   [ 0.082353  ,  0.082353  , -0.25490195],
   ...,
   [ 0.04313731, -0.01960784, -0.19999999],
   [ 0.32549024,  0.26274514, -0.12156862],
   [ 0.3803922 ,  0.2941177 , -0.0745098 ]]],


 ...,


 [[[ 0.5294118 ,  0.1686275 , -0.5058824 ],
   [ 0.5058824 ,  0.14509809, -0.5294118 ],
   [ 0.45882356,  0.09803927, -0.5764706 ],
   ...,
   [ 0.5294118 ,  0.22352946, -0.21568626],
   [ 0.47450984,  0.14509809, -0.3333333 ],
   [ 0.38823533,  0.03529418, -0.49019605]],

  [[ 0.41176474,  0.05098045, -0.62352943],
   [ 0.43529415,  0.07450986, -0.5921569 ],
   [ 0.45098042,  0.09019613, -0.5764706 ],
   ...,
   [ 0.4666667 ,  0.16078436, -0.2862745 ],
   [ 0.45098042,  0.12156868, -0.36470586],
   [ 0.37254906,  0.0196079 , -0.5058824 ]],

  [[ 0.22352946, -0.1372549 , -0.8039216 ],
   [ 0.33333337, -0.02745098, -0.69411767],
   [ 0.43529415,  0.07450986, -0.58431375],
   ...,
   [ 0.36470592,  0.05098045, -0.3960784 ],
   [ 0.41960788,  0.082353  , -0.41176468],
   [ 0.34901965, -0.00392157, -0.5372549 ]],
```

```
 ...,

 [[ 0.23921573, -0.27843136, -0.9372549 ],
  [ 0.254902  , -0.26274508, -0.92156863],
  [ 0.33333337, -0.18431371, -0.84313726],
  ...,
  [ 0.6313726 ,  0.12156868, -0.6       ],
  [ 0.6392157 ,  0.12941182, -0.6       ],
  [ 0.64705884,  0.12941182, -0.60784316]],

 [[ 0.2313726 , -0.2862745 , -0.94509804],
  [ 0.254902  , -0.26274508, -0.92156863],
  [ 0.3411765 , -0.17647058, -0.84313726],
  ...,
  [ 0.62352943,  0.11372554, -0.60784316],
  [ 0.62352943,  0.11372554, -0.6156863 ],
  [ 0.62352943,  0.10588241, -0.6313726 ]],

 [[ 0.22352946, -0.29411763, -0.9529412 ],
  [ 0.26274514, -0.26274508, -0.92156863],
  [ 0.3411765 , -0.17647058, -0.84313726],
  ...,
  [ 0.6156863 ,  0.10588241, -0.6156863 ],
  [ 0.6156863 ,  0.09803927, -0.62352943],
  [ 0.6156863 ,  0.09803927, -0.6392157 ]]],


[[[ 0.52156866,  0.827451  ,  0.94509804],
  [ 0.52156866,  0.827451  ,  0.94509804],
  [ 0.52156866,  0.827451  ,  0.94509804],
  ...,
  [ 0.6862745 ,  0.92156863,  0.92156863],
  [ 0.6392157 ,  0.92156863,  0.90588236],
  [ 0.6392157 ,  0.92156863,  0.90588236]],

 [[ 0.5137255 ,  0.827451  ,  0.94509804],
  [ 0.5137255 ,  0.827451  ,  0.94509804],
  [ 0.5137255 ,  0.827451  ,  0.94509804],
  ...,
  [ 0.6784314 ,  0.9137255 ,  0.9137255 ],
  [ 0.6392157 ,  0.92156863,  0.90588236],
  [ 0.6392157 ,  0.92156863,  0.90588236]],

 [[ 0.49803925,  0.81960785,  0.9372549 ],
  [ 0.49803925,  0.81960785,  0.9372549 ],
  [ 0.49803925,  0.81960785,  0.9372549 ],
  ...,
  [ 0.6627451 ,  0.90588236,  0.90588236],
  [ 0.6313726 ,  0.9137255 ,  0.8980392 ],
  [ 0.6313726 ,  0.9137255 ,  0.8980392 ]],

 ...,

 [[-0.38823527, -0.7019608 , -0.8352941 ],
  [ 0.082353  , -0.20784312, -0.5058824 ],
  [-0.1372549 , -0.40392154, -0.7176471 ],
  ...,
  [ 0.5921569 ,  0.254902  , -0.3333333 ],
  [ 0.41176474,  0.12156868, -0.42745095],
```

```
  [ 0.35686278,  0.05882359, -0.49019605]],

 [[-0.14509803, -0.4588235 , -0.69411767],
  [ 0.13725495, -0.1607843 , -0.45098037],
  [-0.12941176, -0.40392154, -0.70980394],
  ...,
  [ 0.654902  ,  0.3176471 , -0.26274508],
  [ 0.47450984,  0.18431377, -0.36470586],
  [ 0.41960788,  0.12941182, -0.41960782]],

 [[-0.04313725, -0.3490196 , -0.6392157 ],
  [ 0.16078436, -0.1372549 , -0.42745095],
  [-0.12941176, -0.40392154, -0.70980394],
  ...,
  [ 0.6862745 ,  0.34901965, -0.23137254],
  [ 0.49803925,  0.20784318, -0.34117645],
  [ 0.4431373 ,  0.15294123, -0.3960784 ]]],


[[[ 0.69411767,  0.05098045, -1.        ],
  [ 0.8666667 ,  0.22352946, -0.85882354],
  [ 0.7176471 ,  0.082353  , -1.        ],
  ...,
  [ 0.8980392 ,  0.27843142, -0.7176471 ],
  [ 0.7882353 ,  0.1686275 , -0.827451  ],
  [ 0.6784314 ,  0.05098045, -0.81960785]],

 [[ 0.7882353 ,  0.13725495, -0.8666667 ],
  [ 0.827451  ,  0.17647064, -0.85882354],
  [ 0.92156863,  0.27843142, -0.77254903],
  ...,
  [ 0.7490196 ,  0.12941182, -0.8980392 ],
  [ 0.85882354,  0.23921573, -0.77254903],
  [ 0.6156863 , -0.01176471, -0.8980392 ]],

 [[ 0.7176471 ,  0.05098045, -0.8666667 ],
  [ 0.79607844,  0.12941182, -0.8039216 ],
  [ 0.6784314 ,  0.0196079 , -0.9607843 ],
  ...,
  [ 0.6862745 ,  0.07450986, -0.99215686],
  [ 0.8745098 ,  0.26274514, -0.8039216 ],
  [ 0.7254902 ,  0.09019613, -0.8352941 ]],

 ...,

 [[ 0.69411767,  0.0196079 , -0.9137255 ],
  [ 0.5921569 , -0.08235294, -1.        ],
  [ 0.79607844,  0.12156868, -0.8117647 ],
  ...,
  [ 0.54509807, -0.1607843 , -0.75686276],
  [ 0.56078434, -0.15294117, -0.7254902 ],
  [ 0.41960788, -0.3333333 , -0.8117647 ]],

 [[ 0.54509807, -0.1372549 , -1.        ],
  [ 0.38823533, -0.29411763, -1.        ],
  [ 0.64705884, -0.03529412, -0.94509804],
  ...,
  [ 0.27058828, -0.45098037, -0.9764706 ],
  [ 0.15294123, -0.5686275 , -1.        ],
  [ 0.20000005, -0.5529412 , -1.        ]],
```

```
        [[ 0.5058824 , -0.1607843 , -0.8666667 ],
         [ 0.654902  , -0.00392157, -0.77254903],
         [ 0.67058825,  0.00392163, -0.8509804 ],
         ...,
         [ 0.5058824 , -0.18431371, -0.8901961 ],
         [ 0.5686275 , -0.12156862, -0.8039216 ],
         [ 0.39607847, -0.27058822, -0.90588236]]]], dtype=float32), array([[1., 0.,
0., ..., 0., 0., 0.],
       [1., 0., 0., ..., 0., 0., 0.],
       [1., 0., 0., ..., 0., 0., 0.],
       ...,
       [0., 0., 0., ..., 0., 0., 1.],
       [0., 0., 0., ..., 0., 0., 1.],
       [0., 0., 0., ..., 0., 0., 1.]], dtype=float32)), Predicted=[9.9995685e-01 4.09
95194e-05 3.4780383e-11 1.6994706e-10 4.2968244e-07
 2.1204208e-07 1.3828437e-06 2.1488546e-08 3.5806274e-08]
```

In [59]:

```python
y_classes = [np.argmax(element) for element in pred]
print('Predicted_values:',pred[:360])
print('Actual_values:',y_pred[:360])
```

```
Predicted_values: [[9.9995685e-01 4.0995194e-05 3.4780383e-11 ... 1.3828437e-06
  2.1488546e-08 3.5806274e-08]
 [9.3462968e-01 1.4534888e-03 6.3823759e-06 ... 3.1929113e-02
  5.8518199e-05 3.1826448e-02]
 [1.0000000e+00 9.4442525e-09 1.2910436e-12 ... 3.3188505e-09
  4.8667821e-09 3.0076450e-09]
 ...
 [5.3048034e-08 1.3856518e-09 1.2628858e-12 ... 2.6713727e-07
  1.6258477e-09 9.9999952e-01]
 [2.7019971e-03 1.4588927e-01 2.4901814e-07 ... 1.5996138e-02
  3.0035975e-05 3.3177006e-01]
 [1.4236607e-06 2.0642032e-07 2.6244651e-08 ... 1.3151682e-05
  5.8849089e-08 9.9998403e-01]]
Actual_values: [0 0 0 0 0 0 0 0 0 0 0 0 0 0 0 0 0 0 0 0 0 0 0 0 0 0 0 0 6 0 0 0 0 0 0
0 0
 0 0 1 1 1 1 1 1 1 5 1 1 2 1 1 1 5 2 2 1 0 3 1 1 1 1 5 7 0 5 1 1 1 1 1 1 1
 1 1 1 1 1 1 2 2 2 2 2 2 2 2 2 2 2 2 2 2 2 2 2 2 2 2 6 2 2 2 2 2 2 2 2
 2 2 2 2 2 2 2 2 2 3 3 3 3 3 3 3 3 3 3 3 3 3 3 3 3 3 3 4 3 5 3 5 5 7 2 2
 5 2 3 3 3 5 3 3 3 3 3 3 4 4 4 4 4 6 4 4 4 4 4 7 6 0 4 4 4 4 4 4 4 6 4 4 4
 4 6 4 4 6 4 4 4 1 4 4 6 4 4 4 5 5 5 5 5 5 5 5 5 5 5 5 5 5 5 8 0 0 5 7 3 5
 5 5 5 5 5 7 5 5 0 5 0 5 7 5 8 6 5 5 4 6 6 6 6 6 6 6 6 6 6 6 6 6 6 6 6 6 6
 6 6 6 6 6 6 6 6 6 6 6 6 0 6 6 6 6 6 6 6 6 7 7 7 7 7 7 7 7 7 7 7 7 7 7 7 7
 7 7 7 7 7 7 7 7 5 1 7 7 5 7 5 6 7 7 7 7 7 7 5 7 8 8 8 8 8 8 8 8 6 8 8 8 8
 8 0 0 8 8 8 8 8 8 8 8 8 8 8 8 0 8 8 8 8 8 8 8 6 8 4 8]
```

In [60]:

```python
predicted_classes = model.predict(x=test_batches, steps=len(test_batches), verbose=0)
predicted_classes = np.argmax(np.round(predicted_classes),axis=1)
predicted_classes.shape, y_pred.shape
#print(type(predictions), predictions.shape)
```

Out[60]:

```
((360,), (360,))
```

In [61]:

```python
plt.figure(figsize=(10, 10))
incorrect = np.where(predicted_classes!=y_pred)[0]
print (("Found %d incorrect labels") % len(incorrect))
for i, incorrect in enumerate(incorrect[:0]):
    plt.subplot(3,3,i+1)
    plt.imshow(testX[incorrect], cmap='gray', interpolation='none')
```

```
    plt.title("Predicted {}, Class {}".format(predicted_classes[incorrect], y_pred[inc
    plt.tight_layout()
```

```
Found 4 incorrect labels
<Figure size 1000x1000 with 0 Axes>
```

In [62]:
```
y_classes = [np.argmax(element) for element in pred]
print('Predicted_values:',pred[:50])
print('Actual_values:',y_pred[:50])
```

```
Predicted_values: [[9.99956846e-01 4.09951936e-05 3.47803834e-11 1.69947056e-10
  4.29682444e-07 2.12042082e-07 1.38284372e-06 2.14885461e-08
  3.58062735e-08]
 [9.34629679e-01 1.45348883e-03 6.38237589e-06 9.53435233e-07
  6.35011602e-05 3.17888007e-05 3.19291130e-02 5.85181988e-05
  3.18264477e-02]
 [1.00000000e+00 9.44425249e-09 1.29104355e-12 4.92525779e-13
  3.70910369e-09 6.60354271e-10 3.31885053e-09 4.86678209e-09
  3.00764502e-09]
 [9.99995232e-01 3.14334739e-06 5.70018546e-11 1.29813771e-10
  4.57651446e-08 8.39929157e-07 9.38477118e-09 3.37945238e-07
  5.29958584e-07]
 [9.99933958e-01 2.00049999e-06 2.72260392e-09 2.03705373e-08
  3.16066391e-08 8.73086847e-08 6.36146069e-05 8.96800358e-08
  2.59178762e-07]
 [9.99991059e-01 2.61138644e-06 2.33291747e-10 1.70506631e-08
  3.93569508e-07 2.79137424e-07 5.51875974e-06 1.48729873e-08
  7.45667705e-08]
 [9.99999762e-01 1.86872184e-09 1.05960232e-11 2.12134185e-10
  4.07364666e-08 4.08446471e-10 1.09717121e-07 2.23961170e-08
  6.96811497e-08]
 [9.99982595e-01 2.11797619e-08 1.64629625e-06 1.09406592e-07
  5.39684919e-08 3.92586870e-08 9.12795258e-06 6.11808036e-06
  3.38062819e-07]
 [9.99963999e-01 6.26305609e-06 3.49671136e-09 4.31736822e-07
  2.23750035e-07 1.47858532e-06 1.40970651e-05 6.89102762e-07
  1.28602987e-05]
 [9.99996662e-01 3.67172914e-09 2.86886118e-12 1.90576199e-09
  2.34464270e-08 1.89300997e-09 6.47214620e-07 2.39902676e-08
  2.78392076e-06]
 [9.99968410e-01 7.49243418e-06 7.56571623e-08 1.41531435e-08
  1.47540140e-06 1.03658713e-05 1.59304363e-06 1.02927388e-05
  3.43919453e-07]
 [9.97479618e-01 9.61884798e-05 4.50889318e-04 1.10151457e-04
  2.19028170e-05 4.18388314e-04 3.39262566e-04 8.41597328e-04
  2.42019232e-04]
 [9.99954581e-01 5.17232411e-06 7.34832507e-12 1.48643778e-10
  7.35090634e-06 2.65077290e-07 2.96197741e-05 2.89277978e-06
  1.54670289e-07]
 [9.99908686e-01 3.68831650e-08 5.53356874e-11 1.63019223e-10
  1.92566176e-08 2.73252496e-08 4.52347166e-07 8.92868920e-05
  1.44075705e-06]
 [9.86257792e-01 1.16822928e-04 1.33363316e-02 3.80997585e-06
  1.39306969e-04 1.19440345e-04 1.84739911e-05 5.57936210e-06
  2.46803779e-06]
 [1.00000000e+00 6.85753815e-11 5.69274987e-14 8.92487148e-13
  2.12353156e-11 9.42594058e-11 1.88664706e-09 5.37641737e-11
  2.80650156e-11]
 [9.97898102e-01 1.62461679e-03 6.01992767e-09 9.22280492e-08
  3.08843970e-04 1.61243606e-05 1.51552580e-04 4.46829688e-07
  2.94001580e-07]
 [9.98316407e-01 1.34566616e-08 5.53239721e-09 1.28837359e-08
  7.47221378e-08 2.44530147e-05 1.61583330e-06 1.39029312e-03
  2.67035444e-04]
 [9.99992847e-01 1.39876576e-07 1.45789392e-09 2.07683715e-09
  8.30873930e-08 3.53382347e-07 6.26216732e-08 4.16445118e-06
  2.31096442e-06]
 [1.00000000e+00 8.27745961e-09 3.54237798e-13 2.74894517e-11
  3.29584277e-10 3.58751535e-08 1.69435632e-09 4.66548977e-09
  3.57579077e-09]
```

```
[1.00000000e+00 3.96965620e-11 2.68847271e-12 9.33517066e-12
 1.56665514e-10 3.25350025e-11 2.39189344e-08 5.74279735e-10
 2.09660134e-09]
[9.99984860e-01 1.55514560e-10 4.41499224e-12 1.07859534e-10
 1.33860718e-08 5.06907973e-08 2.70634558e-07 6.40802682e-08
 1.47835171e-05]
[9.99995708e-01 9.20406720e-12 1.72629207e-12 1.08696446e-11
 7.27253413e-09 2.59515909e-10 4.34432968e-06 1.57756154e-12
 2.86395185e-10]
[9.99351203e-01 3.37724690e-04 3.62091157e-07 2.20261626e-07
 3.21319760e-07 3.21736508e-07 1.69146751e-05 2.25149142e-05
 2.70427641e-04]
[9.94664073e-01 7.03027690e-05 3.47296492e-07 5.02449438e-07
 4.95631248e-03 1.72515847e-05 2.31657905e-04 4.75885172e-05
 1.18273974e-05]
[9.97442722e-01 7.11952162e-05 1.20589484e-05 3.13745386e-06
 1.11170239e-05 8.19152956e-06 1.04830635e-03 5.32229722e-04
 8.71059601e-04]
[9.05163586e-01 5.36845298e-04 6.12017029e-05 6.38434649e-05
 7.84907315e-04 4.26206789e-05 3.12178694e-02 2.49669584e-03
 5.96324615e-02]
[9.99999523e-01 5.22579369e-10 2.07656756e-11 7.16812200e-12
 2.33751720e-07 4.73847050e-09 3.06670644e-09 1.52424050e-07
 1.35779260e-07]
[4.96408865e-02 3.09819416e-06 2.31210997e-08 2.46599302e-06
 2.49069097e-04 6.04166075e-07 5.88236034e-01 6.74992989e-05
 3.61800343e-01]
[7.00945973e-01 1.50751206e-04 1.17035692e-04 5.17393164e-02
 1.60490497e-04 1.52967721e-01 1.02653685e-04 9.37152505e-02
 1.00852878e-04]
[9.99999762e-01 8.86260954e-10 1.12496824e-13 5.90179964e-14
 4.36423733e-08 5.15098186e-10 1.58605729e-07 3.66007585e-10
 1.57823251e-07]
[9.99999285e-01 5.57694051e-08 4.49224075e-10 4.95399295e-12
 7.58669998e-08 8.88712606e-08 5.16936964e-08 6.14247995e-08
 4.12636950e-07]
[7.90441811e-01 3.69166606e-04 1.11532779e-06 4.00363263e-07
 2.08869874e-01 3.42257681e-06 2.82808760e-04 1.90309868e-06
 2.94868041e-05]
[1.00000000e+00 2.47724535e-10 3.23366279e-13 7.83502013e-11
 5.92870752e-11 5.66228897e-10 7.66518760e-09 4.54537341e-09
 5.49694423e-09]
[9.99983072e-01 4.89739378e-08 7.78593075e-12 1.71179841e-11
 1.05758080e-09 3.75662211e-07 1.62515530e-08 1.38871828e-05
 2.61805826e-06]
[9.99999762e-01 5.39254752e-12 6.06633233e-13 7.50499662e-12
 8.98663810e-09 2.07808199e-07 2.05511963e-09 4.16375698e-08
 3.65687458e-09]
[9.99996662e-01 9.66695893e-07 1.12464246e-10 7.86097992e-11
 9.27976629e-09 2.00589443e-06 6.14021101e-09 2.51598266e-07
 6.68605935e-08]
[9.99982357e-01 1.76816840e-07 8.16678170e-10 4.37545083e-10
 7.70549548e-07 1.53061937e-05 1.42835759e-07 3.38317847e-07
 9.82092388e-07]
[9.99992728e-01 1.85070803e-08 6.41278142e-09 5.25295263e-07
 1.82661125e-07 4.61368609e-06 4.23797161e-07 1.32387436e-06
 1.39789321e-07]
[1.77145272e-01 8.18032920e-01 1.34696253e-04 2.56346411e-05
 5.48509306e-05 4.09874320e-03 2.78097723e-05 4.74880682e-04
 5.14588510e-06]
```

```
 [6.66871294e-02 9.26446319e-01 3.92270420e-04 2.63626771e-05
  7.54206048e-05 2.90110765e-04 4.88340249e-03 7.76289671e-05
  1.12133729e-03]
 [3.44083505e-03 9.85637248e-01 3.65189742e-03 9.34631430e-07
  6.67651111e-05 7.14050094e-03 2.19395170e-05 3.69493755e-05
  2.90340108e-06]
 [8.69206906e-06 9.99294043e-01 1.08752101e-04 3.17839124e-08
  8.50591732e-07 5.69906377e-04 3.93862911e-06 1.36493727e-05
  1.29451323e-07]
 [4.12538611e-05 9.97145116e-01 1.87437426e-08 3.40535564e-08
  6.89039226e-08 2.36324011e-03 2.71567643e-07 4.49660060e-04
  3.83317030e-07]
 [1.03244602e-05 9.96215761e-01 3.05517897e-05 5.17586770e-04
  6.83426606e-07 2.64041126e-03 9.69401299e-06 4.55627305e-05
  5.29258745e-04]
 [9.08612634e-08 9.98117208e-01 7.58005946e-04 6.67725590e-06
  8.97464124e-05 7.30774540e-04 2.57731085e-06 5.62083699e-07
  2.94413127e-04]
 [4.39170326e-05 2.14408174e-05 5.40949600e-07 2.56306157e-06
  8.11446853e-07 9.99928951e-01 2.80172998e-07 1.04484184e-06
  4.49740128e-07]
 [1.85514909e-06 9.85593259e-01 1.12292901e-06 1.33397942e-02
  3.40696033e-07 9.25984641e-04 1.74513293e-08 1.37136012e-04
  5.83979670e-07]
 [3.44000146e-04 9.95288372e-01 4.76585910e-07 6.48846708e-06
  1.82410087e-08 4.37941460e-04 1.11072227e-08 3.92078795e-03
  1.90023934e-06]
 [4.27321240e-04 4.38786119e-01 4.97592360e-01 1.23960851e-03
  3.11726399e-05 4.46583815e-02 8.77089678e-06 1.71660967e-02
  9.02614338e-05]]
Actual_values: [0 0 0 0 0 0 0 0 0 0 0 0 0 0 0 0 0 0 0 0 0 0 0 0 0 0 0 0 6 0 0 0 0 0
0 0
 0 0 1 1 1 1 1 1 1 5 1 1 2]
```

In [63]:
```python
plt.figure()
plt.imshow(testX[60])
plt.colorbar()
plt.show()
```

```
Clipping input data to the valid range for imshow with RGB data ([0..1] for floats or
[0..255] for integers).
```

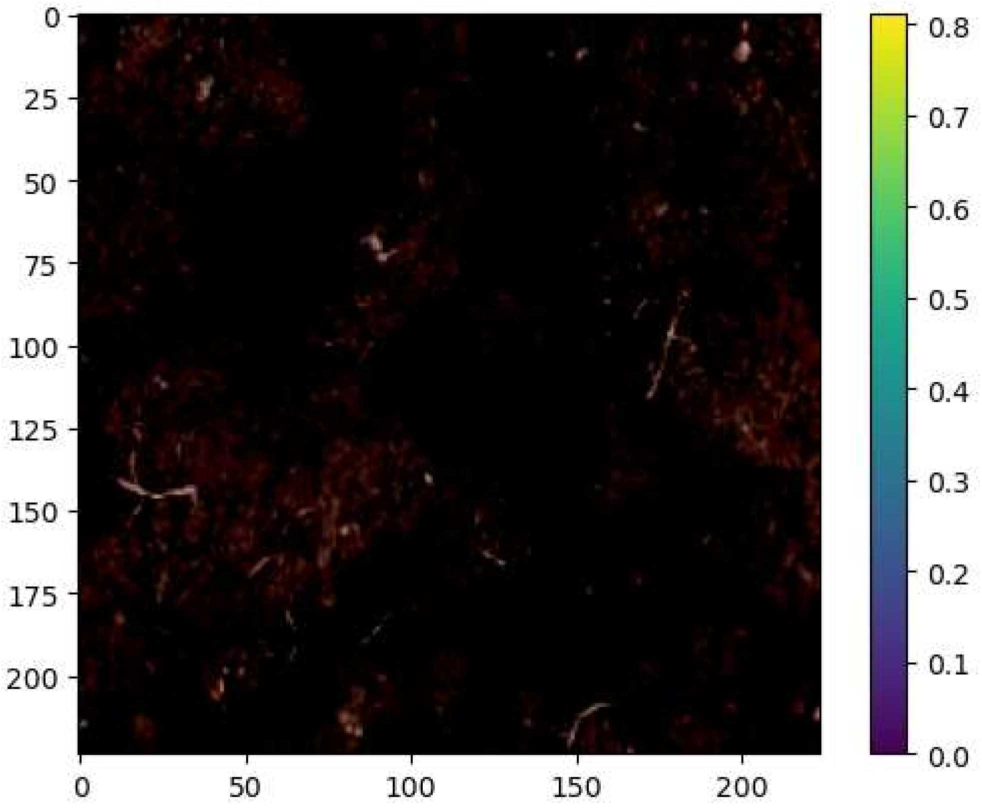

In [64]:
```
#printing the first element from predicted data
pred=model.predict(test_batches)
print(pred[60])
#printing the index of
print('Index:',np.argmax(pred[60]))
```

```
1/1 [==============================] - 9s 9s/step
[3.9867871e-03 9.9434328e-01 1.0524897e-04 1.8053751e-04 4.4848872e-05
 1.0270147e-03 1.4145350e-05 2.9336289e-04 4.8246934e-06]
Index: 1
```

In [65]:
```
i=60
plt.figure(figsize=(6,3))
plt.subplot(1,2,1)
plot_image(i, predictions[i], test_labels, testX)
plt.subplot(1,2,2)
plot_value_array(i, predictions[i], test_labels)
plt.show()
```

```
Clipping input data to the valid range for imshow with RGB data ([0..1] for floats or
[0..255] for integers).
```

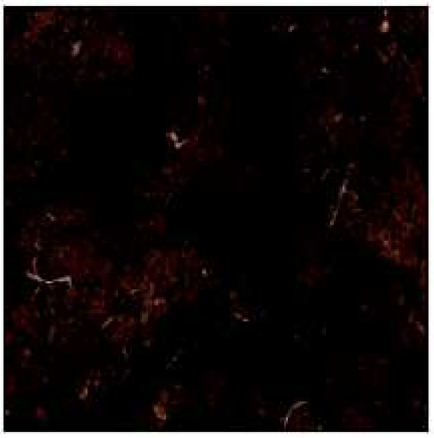

Alluvial 99% (Alluvial)

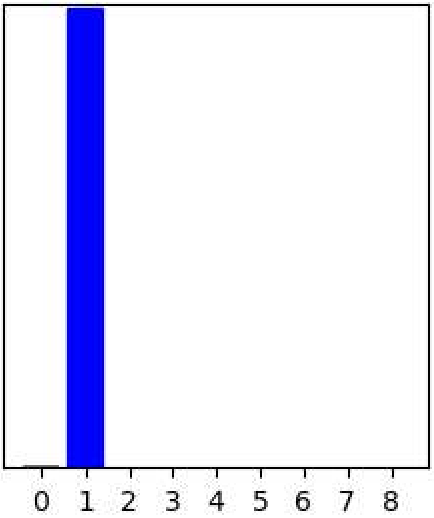

In [67]:
```
i=1
plt.figure(figsize=(6,3))
plt.subplot(1,2,1)
plot_image(i, predictions[i], test_labels, testX)
plt.subplot(1,2,2)
plot_value_array(i, predictions[i], test_labels)
plt.show()
```

```
Clipping input data to the valid range for imshow with RGB data ([0..1] for floats or
[0..255] for integers).
```

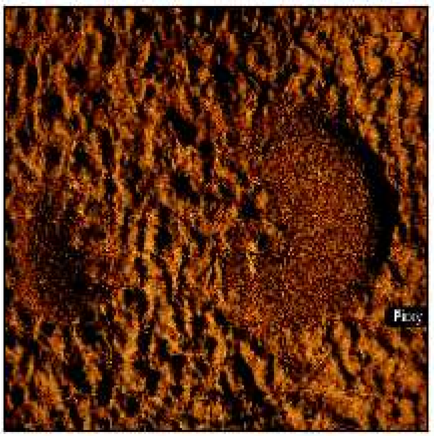

Arid 93% (Arid)

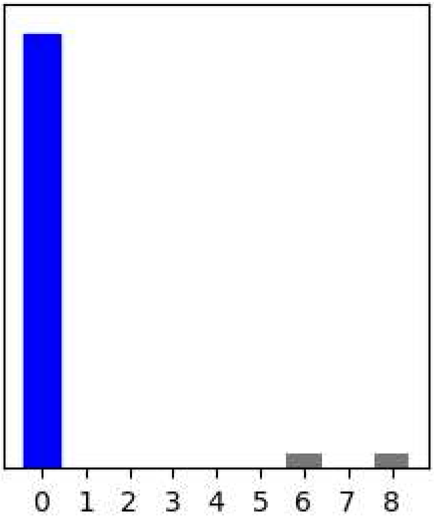

In [68]:
```
i=11
plt.figure(figsize=(6,3))
plt.subplot(1,2,1)
plot_image(i, predictions[i], test_labels, testX)
plt.subplot(1,2,2)
plot_value_array(i, predictions[i], test_labels)
plt.show()
```

```
Clipping input data to the valid range for imshow with RGB data ([0..1] for floats or
[0..255] for integers).
```

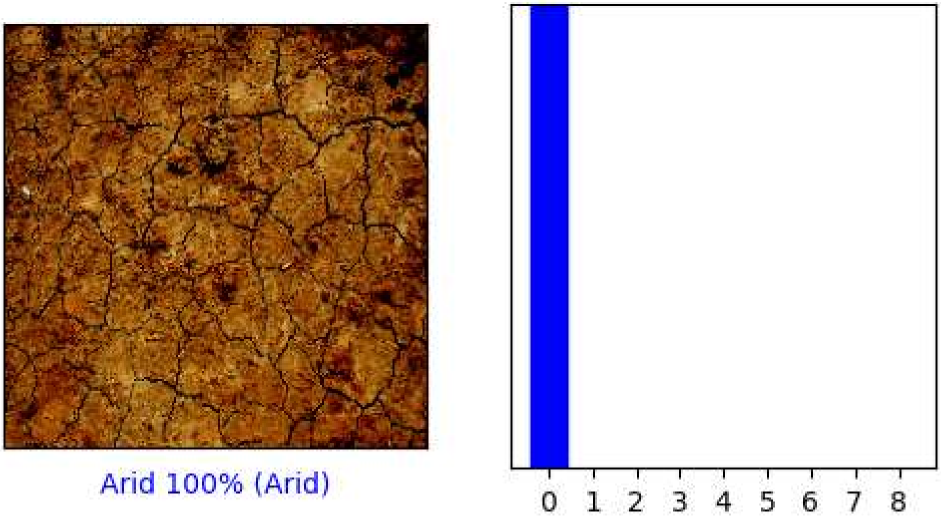

In [69]:
```
i=26
plt.figure(figsize=(6,3))
plt.subplot(1,2,1)
plot_image(i, predictions[i], test_labels, testX)
plt.subplot(1,2,2)
plot_value_array(i, predictions[i], test_labels)
plt.show()
```

```
Clipping input data to the valid range for imshow with RGB data ([0..1] for floats or
[0..255] for integers).
```

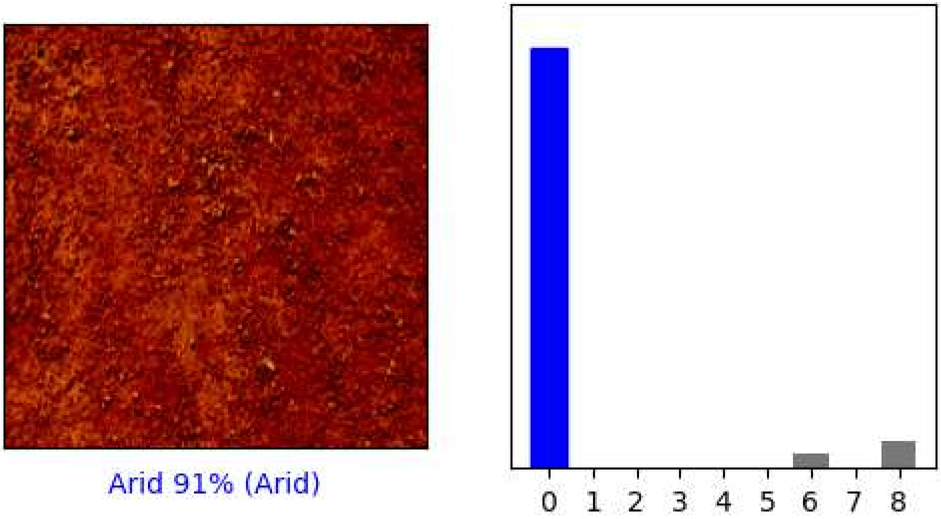

In [70]:
```
i=32
plt.figure(figsize=(6,3))
plt.subplot(1,2,1)
plot_image(i, predictions[i], test_labels, testX)
plt.subplot(1,2,2)
plot_value_array(i, predictions[i], test_labels)
plt.show()
```

```
Clipping input data to the valid range for imshow with RGB data ([0..1] for floats or
[0..255] for integers).
```

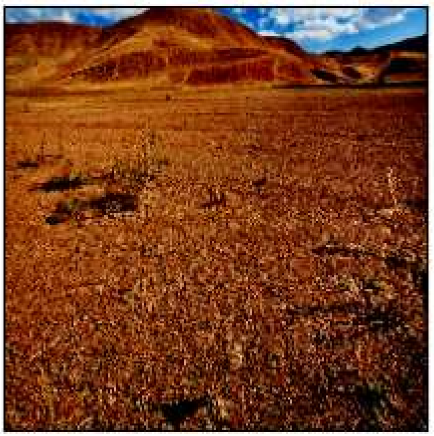

Arid 79% (Arid)

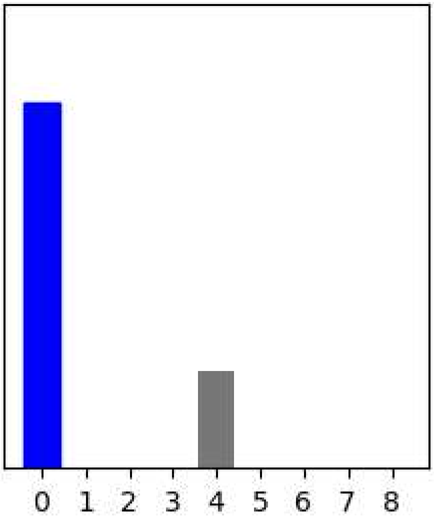

In [71]:
```
i=39
plt.figure(figsize=(6,3))
plt.subplot(1,2,1)
plot_image(i, predictions[i], test_labels, testX)
plt.subplot(1,2,2)
plot_value_array(i, predictions[i], test_labels)
plt.show()
```

```
Clipping input data to the valid range for imshow with RGB data ([0..1] for floats or [0..255] for integers).
```

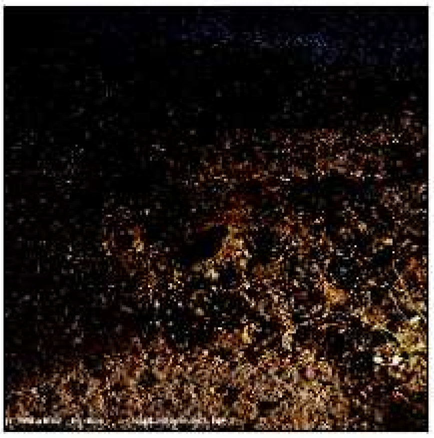

Alluvial 82% (Arid)

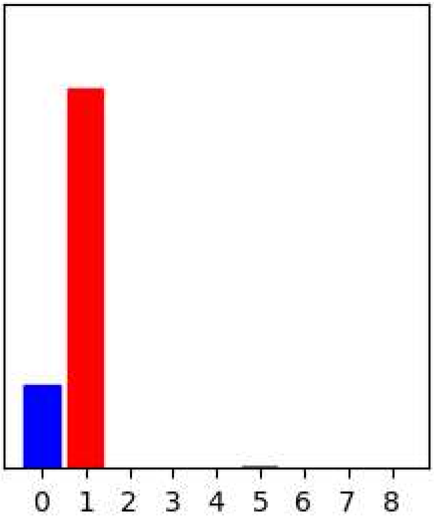

In [72]:
```
i=41
plt.figure(figsize=(6,3))
plt.subplot(1,2,1)
plot_image(i, predictions[i], test_labels, testX)
plt.subplot(1,2,2)
plot_value_array(i, predictions[i], test_labels)
plt.show()
```

```
Clipping input data to the valid range for imshow with RGB data ([0..1] for floats or [0..255] for integers).
```

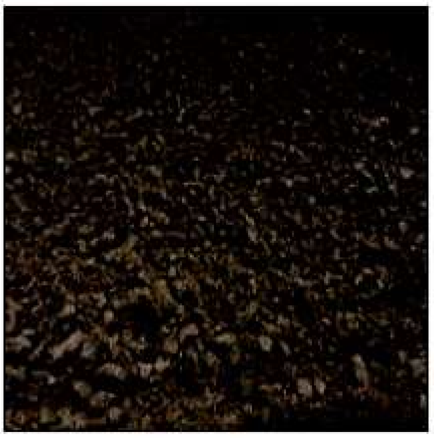

Alluvial 99% (Alluvial)

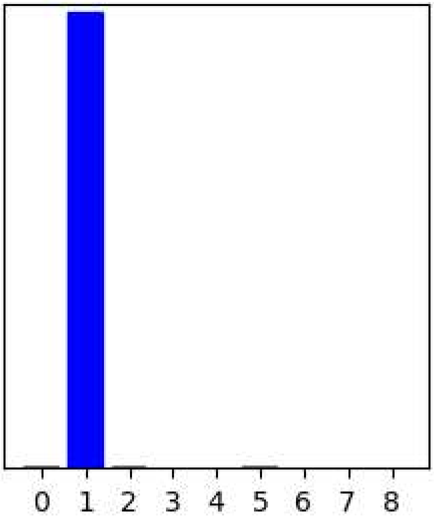

In [73]:

```
i=42
plt.figure(figsize=(6,3))
plt.subplot(1,2,1)
plot_image(i, predictions[i], test_labels, testX)
plt.subplot(1,2,2)
plot_value_array(i, predictions[i], test_labels)
plt.show()
```

```
Clipping input data to the valid range for imshow with RGB data ([0..1] for floats or [0..255] for integers).
```

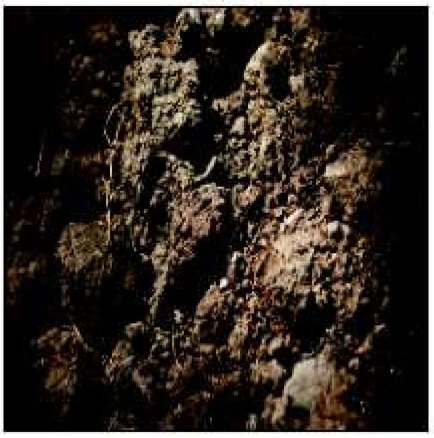

Alluvial 100% (Alluvial)

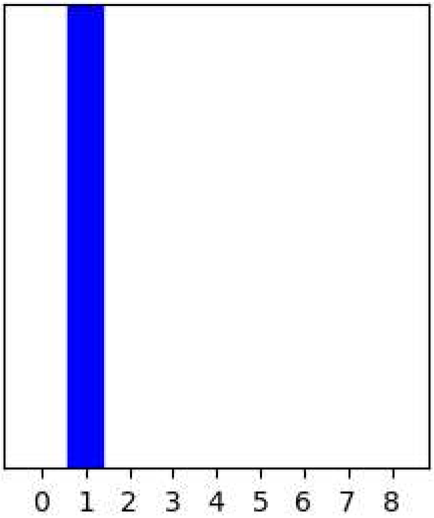

In [74]:

```
i=43
plt.figure(figsize=(6,3))
plt.subplot(1,2,1)
plot_image(i, predictions[i], test_labels, testX)
plt.subplot(1,2,2)
plot_value_array(i, predictions[i], test_labels)
plt.show()
```

```
Clipping input data to the valid range for imshow with RGB data ([0..1] for floats or [0..255] for integers).
```

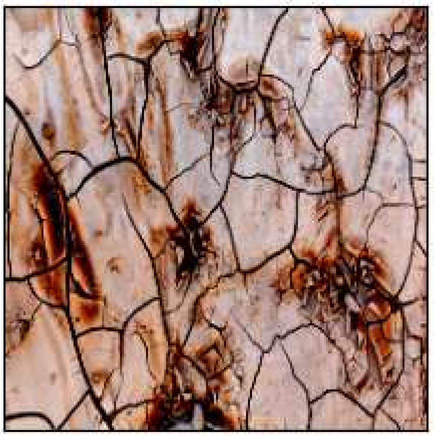

Alluvial 100% (Alluvial)

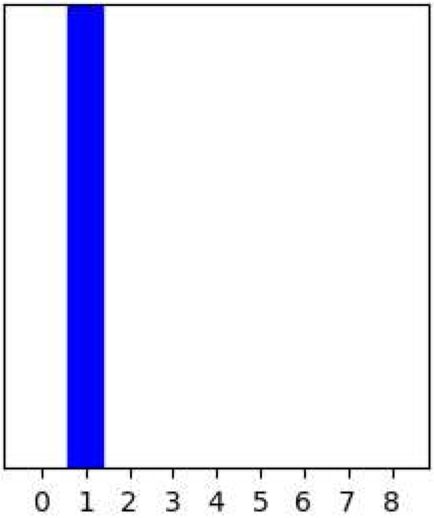

In [75]:
```
# plot feature map of first conv layer for given image
from tensorflow.keras.preprocessing.image import load_img
from tensorflow.keras.preprocessing.image import img_to_array
from matplotlib import pyplot
from numpy import expand_dims
# load the model
model = Model(inputs=model.inputs,outputs=model.layers[1].output)
model.summary()
#model.summary()
# load the image with the required shape
#img = load_img('C:/Users/GyasiEmmanuelKwabena/Desktop/SOILNET/train/RE/RE_372.png',ta
#img = load_img('C:/Users/GyasiEmmanuelKwabena/Desktop/SOILNET/train/AD/AD_309.png',ta
#img = load_img('C:/Users/GyasiEmmanuelKwabena/Desktop/SOILNET/train/AL/AL_328.png',ta
#img = load_img('C:/Users/GyasiEmmanuelKwabena/Desktop/SOILNET/train/BL/BL_577.png',ta
img = load_img('C:/Users/GyasiEmmanuelKwabena/Desktop/SOILNET/train/YE/YE_292.png',tar
# convert the image to an array
img = img_to_array(img)
# expand dimensions so that it represents a single 'sample'
img = expand_dims(img, axis=0)
# prepare the image (e.g. scale pixel values for the vgg)
img = preprocess_input(img)
# get feature map for first hidden layer
feature_maps = model.predict(img)
# plot all 64 maps in an 8x8 squares
square = 4
ix = 1
pyplot.figure(figsize=(224,224))
for _ in range(square):
    for _ in range(square):
 # specify subplot and turn of axis
        ax = pyplot.subplot(square, square, ix)
    ax.set_xticks([])
    ax.set_yticks([])
 # plot filter channel in grayscale
    pyplot.imshow(feature_maps[0, :, :, ix-1], cmap='Set3')
    ix +=1
# show the figure
pyplot.show()
```

```
Model: "model_1"
_________________________________________________________________
 Layer (type)                Output Shape              Param #   
=================================================================
 input_2 (InputLayer)        [(None, None, None, 3)]   0         
                                                                 
 conv1 (Conv2D)              (None, None, None, 32)    864       
                                                                 
=================================================================
Total params: 864
Trainable params: 0
Non-trainable params: 864
_________________________________________________________________
1/1 [==============================] - 0s 37ms/step
```

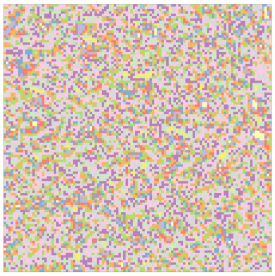
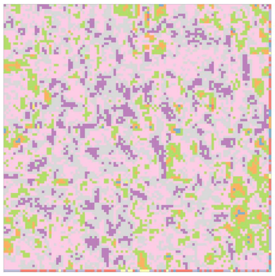
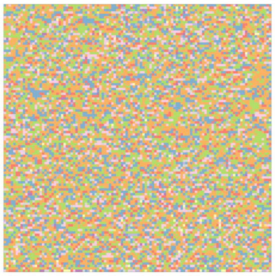
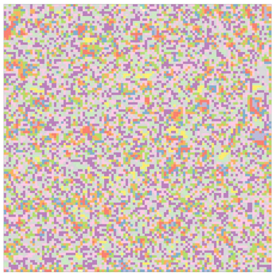

In [76]:
```python
i=10
plt.figure(figsize=(6,3))
plt.subplot(1,2,1)
plot_image(i, predictions[i], test_labels, testX)
plt.subplot(1,2,2)
plot_value_array(i, predictions[i], test_labels)
plt.show()
```

```
Clipping input data to the valid range for imshow with RGB data ([0..1] for floats or [0..255] for integers).
```

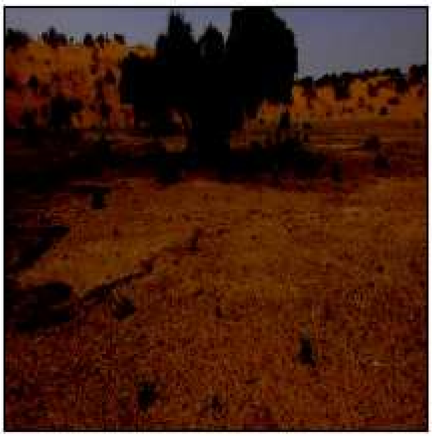

Arid 100% (Arid)

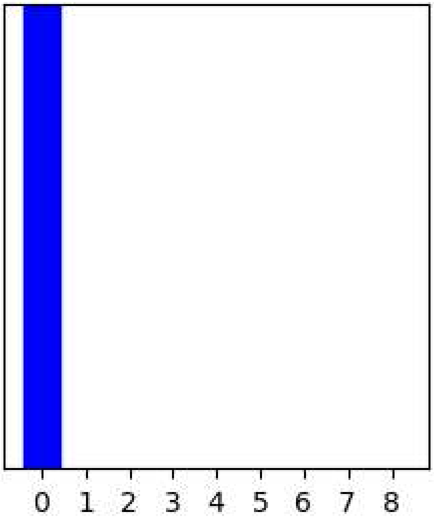

In [77]:
```python
i=84
plt.figure(figsize=(6,3))
plt.subplot(1,2,1)
plot_image(i, predictions[i], test_labels, testX)
plt.subplot(1,2,2)
plot_value_array(i, predictions[i], test_labels)
plt.show()
```

```
Clipping input data to the valid range for imshow with RGB data ([0..1] for floats or
[0..255] for integers).
```

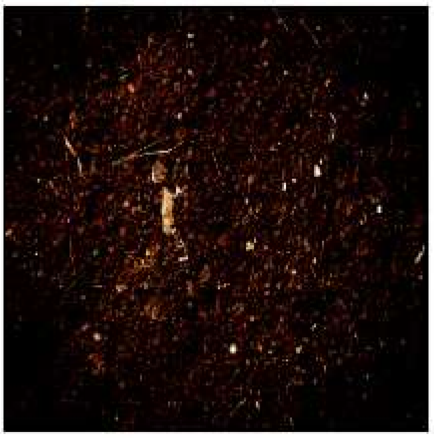

Black 100% (Black)

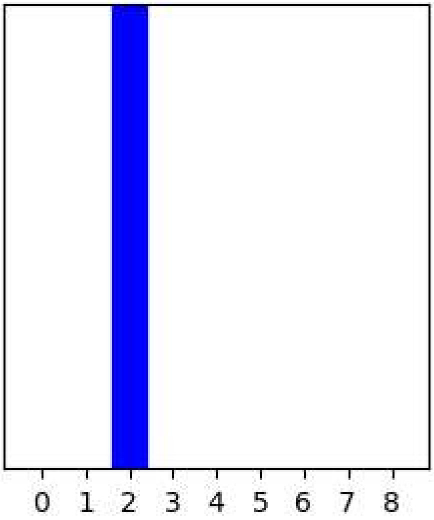

In [78]:

```python
i=123
plt.figure(figsize=(6,3))
plt.subplot(1,2,1)
plot_image(i, predictions[i], test_labels, testX)
plt.subplot(1,2,2)
plot_value_array(i, predictions[i], test_labels)
plt.show()
```

```
Clipping input data to the valid range for imshow with RGB data ([0..1] for floats or
[0..255] for integers).
```

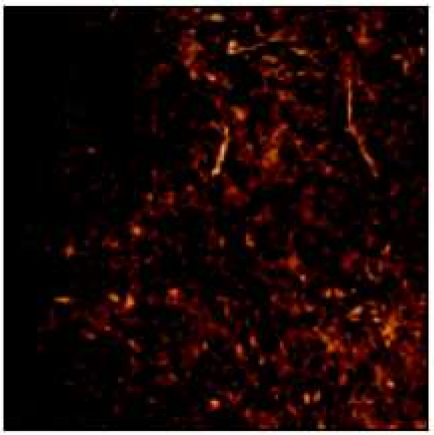

Forest 100% (Forest)

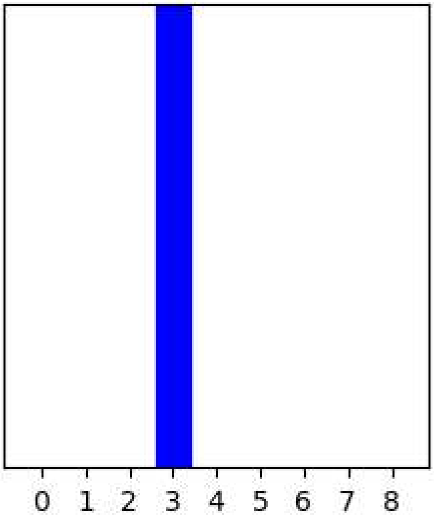

In [79]:

```python
i=125
plt.figure(figsize=(6,3))
plt.subplot(1,2,1)
plot_image(i, predictions[i], test_labels, testX)
plt.subplot(1,2,2)
plot_value_array(i, predictions[i], test_labels)
plt.show()
```

```
Clipping input data to the valid range for imshow with RGB data ([0..1] for floats or
[0..255] for integers).
```

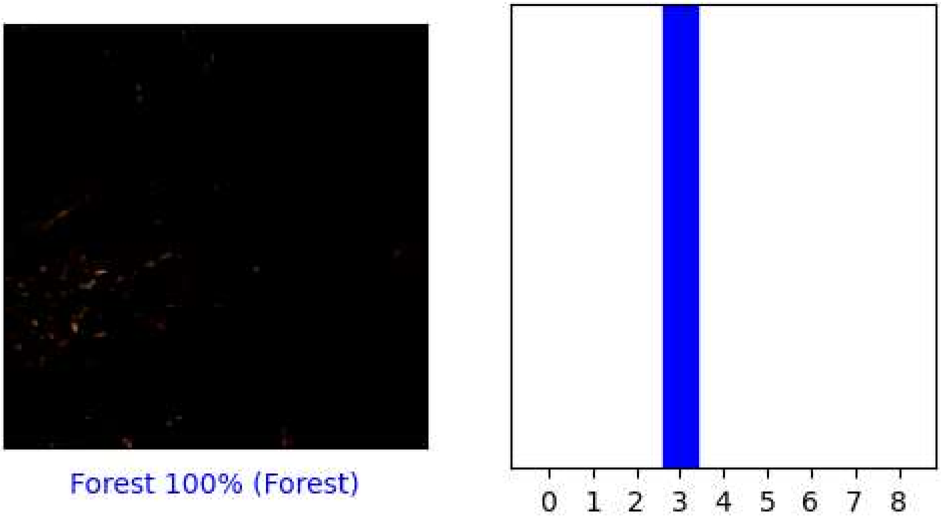

In [80]:
```
i=164
plt.figure(figsize=(6,3))
plt.subplot(1,2,1)
plot_image(i, predictions[i], test_labels, testX)
plt.subplot(1,2,2)
plot_value_array(i, predictions[i], test_labels)
plt.show()
```

```
Clipping input data to the valid range for imshow with RGB data ([0..1] for floats or [0..255] for integers).
```

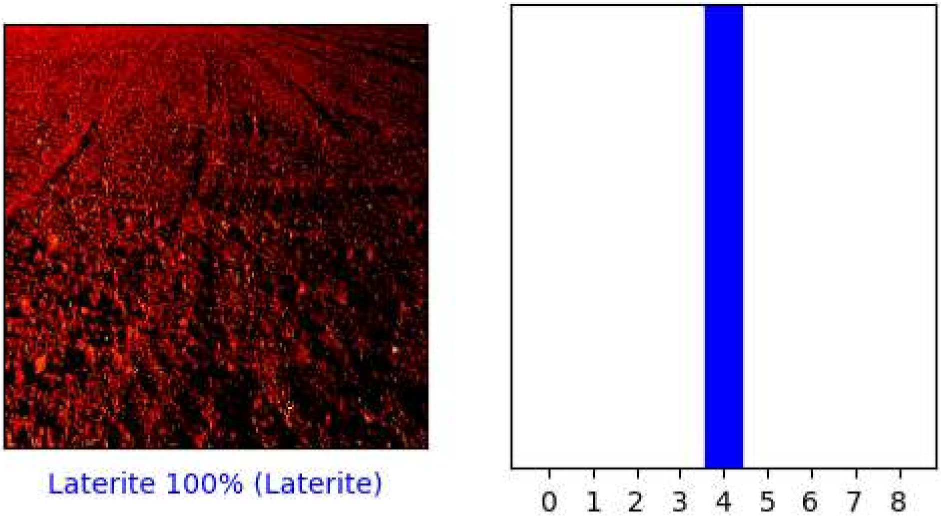

In [81]:
```
i=166
plt.figure(figsize=(6,3))
plt.subplot(1,2,1)
plot_image(i, predictions[i], test_labels, testX)
plt.subplot(1,2,2)
plot_value_array(i, predictions[i], test_labels)
plt.show()
```

```
Clipping input data to the valid range for imshow with RGB data ([0..1] for floats or [0..255] for integers).
```

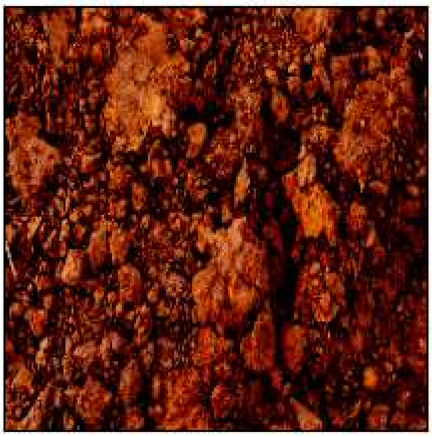

Laterite 100% (Laterite)

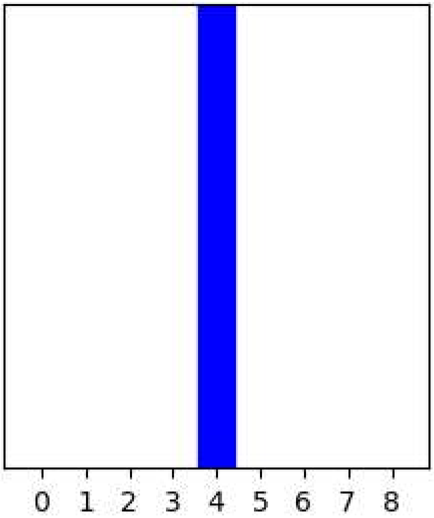

In [82]:

```
i=200
plt.figure(figsize=(6,3))
plt.subplot(1,2,1)
plot_image(i, predictions[i], test_labels, testX)
plt.subplot(1,2,2)
plot_value_array(i, predictions[i], test_labels)
plt.show()
```

```
Clipping input data to the valid range for imshow with RGB data ([0..1] for floats or
[0..255] for integers).
```

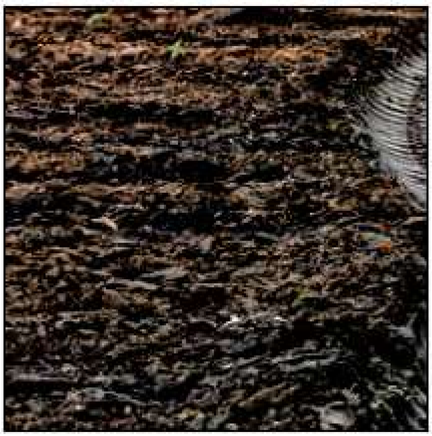

Peaty/Marshy 98% (Peaty/Marshy)

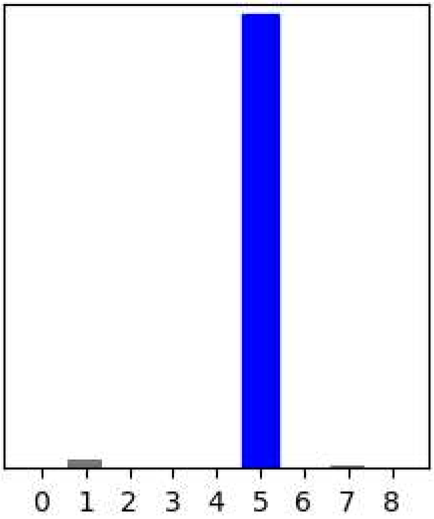

In [83]:

```
i=45
plt.figure(figsize=(6,3))
plt.subplot(1,2,1)
plot_image(i, predictions[i], test_labels, testX)
plt.subplot(1,2,2)
plot_value_array(i, predictions[i], test_labels)
plt.show()
```

```
Clipping input data to the valid range for imshow with RGB data ([0..1] for floats or
[0..255] for integers).
```

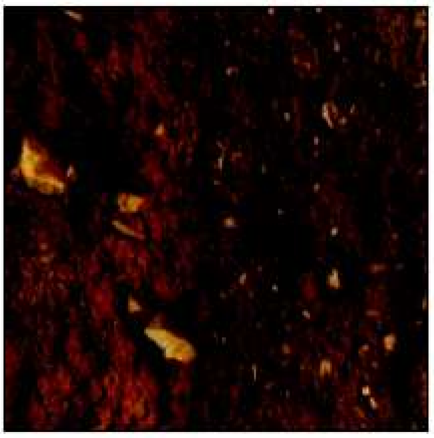

Alluvial 100% (Alluvial)

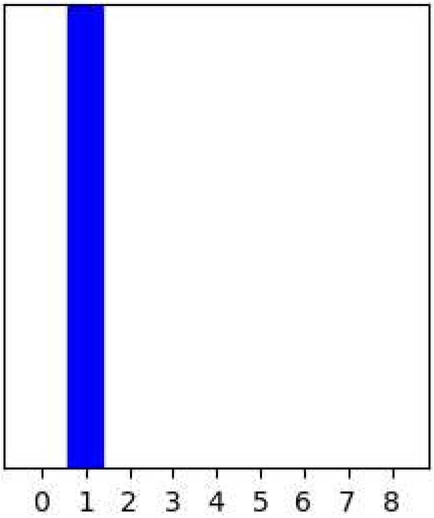

In [85]:
```
i=334
plt.figure(figsize=(6,3))
plt.subplot(1,2,1)
plot_image(i, predictions[i], test_labels, testX)
plt.subplot(1,2,2)
plot_value_array(i, predictions[i], test_labels)
plt.show()
```

```
Clipping input data to the valid range for imshow with RGB data ([0..1] for floats or
[0..255] for integers).
```

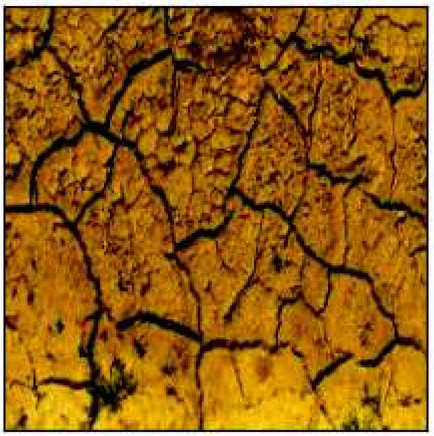

Arid 100% (Yellow)

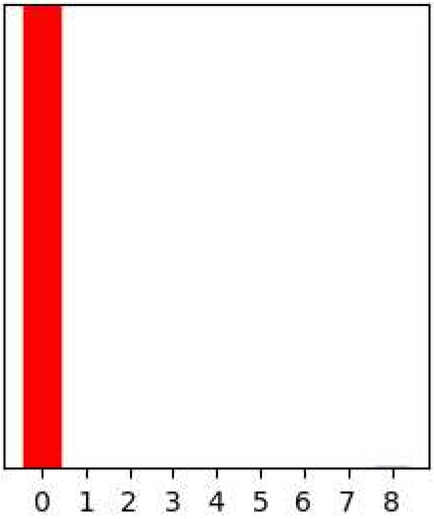

Supplement: Supplementary file 1 [file sensors-23-06709-s001.zip › sensors-2466617-supplementary.pdf]
